# The Conservation and Management of Tunas and Their Relatives: Setting Life History Research Priorities

**DOI:** 10.1371/journal.pone.0070405

**Published:** 2013-08-08

**Authors:** Maria José Juan-Jordá, Iago Mosqueira, Juan Freire, Nicholas K. Dulvy

**Affiliations:** 1 Grupo de Recursos Marinos y Pesquerías, Facultad de Ciencias, Universidad de A Coruña, A Coruña, Spain; 2 Earth to Ocean Research Group, Department of Biological Sciences, Simon Fraser University, Burnaby, British Columbia, Canada; 3 AZTI-Tecnalia, Pasaia, Spain; 4 European Commission, Joint Research Center, IPSC/Maritime Affairs Unit, FISHREG, Ispra VA, Italy; 5 Barrabés Next, Madrid, Spain; Aristotle University of Thessaloniki, Greece

## Abstract

Scombrids (tunas, bonitos, Spanish mackerels and mackerels) support important fisheries in tropical, subtropical and temperate waters around the world, being one of the most economically- and socially-important marine species globally. Their sustainable exploitation, management and conservation depend on accurate life history information for the development of quantitative fisheries stock assessments, and in the fishery data-poor situations for the identification of vulnerable species. Here, we assemble life history traits (maximum size, growth, longevity, maturity, fecundity, spawning duration and spawning interval) for the 51 species of scombrids globally. We identify major biological gaps in knowledge and prioritize life history research needs in scombrids based on their biological gaps in knowledge, the importance of their fisheries and their current conservation status according to the International Union for Conservation of Nature Red List. We find that the growth and reproductive biology of tunas and mackerel species have been more extensively studied than for Spanish mackerels and bonitos, although there are notable exceptions in all groups. We also reveal that reproductive biology of species, particular fecundity, is the least studied biological aspect in scombrids. We identify two priority groups, including 32 species of scombrids, and several populations of principal market tunas, for which life history research should be prioritized following the species-specific life history gaps identified in this study in the coming decades. By highlighting the important gaps in biological knowledge and providing a priority setting for life history research in scombrid species this study provides guidance for management and conservation and serves as a guide for biologists and resource managers interested in the biology, ecology, and management of scombrid species.

## Introduction

**Figure 1:**
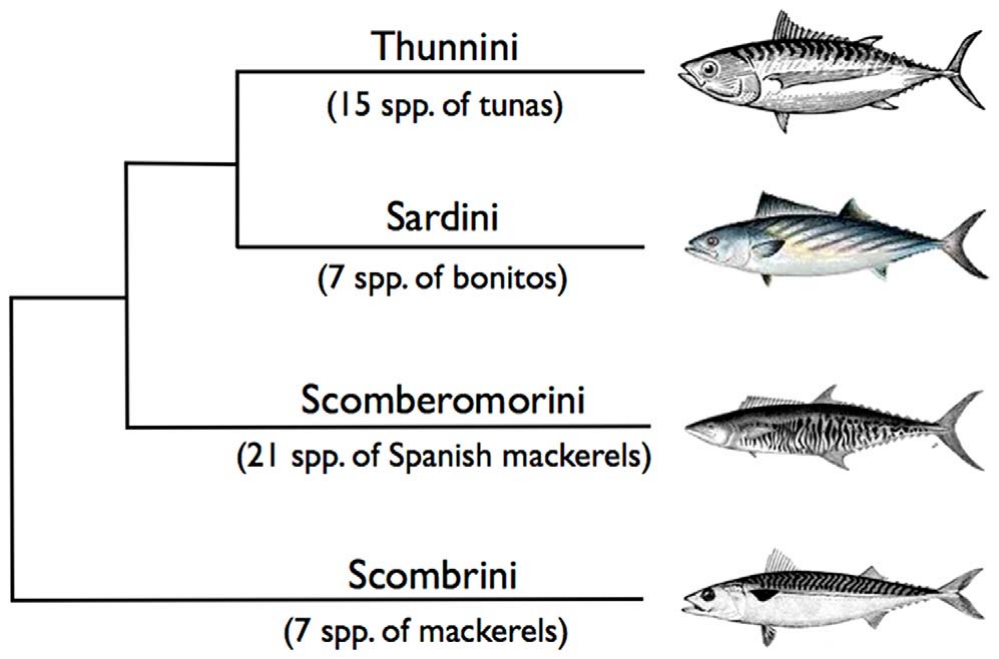
Phylogeny of the family Scombridae showing the four tribes of the subfamily Scombrinae [58]. The subfamily Gasterochismatinae, which has only one species, butterfly kingfish *Gasterochisma melampus*, is not shown.

Life history information such as growth, age and maturity are fundamental determinants of the population dynamics of fishes and underpin the sustainable exploitation and management of species [1]–[3]. As a result, in the last fifty years there has been considerable effort devoted to the analysis of fish life histories. However, even in the era of powerful databases, e.g. FishBase, this information often remains scattered, incomplete and not readily accessible [4], [5]. Here, we compile life history studies for the 51 species of the family Scombridae, commonly known as tunas, bonitos, Spanish mackerels and mackerels (Table 1 and Figure 1). We aim to promote the best use of the existing life history information, synthesize the current knowledge on life history traits across species and identify priority biological research needs in an effort to inform management and conservation of this important group of species in the coming decades.

**Table 1:** List of scombrid species including their taxonomic classification, maximum body size (L_max_), climate, environment and geographic distributions.

Taxonomic group	Latin name	Common name	L_max_	Climate	Environment	Geographical distribution
**Tribe Thunnini (tunas)**	* *Thunnus alalunga*	Albacore tuna	135	Subtropical	Oceanic	Atlantic, Pacific and Indian oceans, including the Mediterranean Sea
	* *Thunnus albacares*	Yellowfin tuna	231	Tropical	Oceanic	Atlantic, Pacific and Indian oceans
	*Thunnus atlanticus*	Blackfin tuna	104	Tropical	Neritic	Western Atlantic Ocean
	* *Thunnus maccoyii*	Southern bluefin tuna	245	Temperate	Oceanic	Southern waters of the Atlantic, Pacific and Indian oceans
	* *Thunnus obesus*	Bigeye tuna	236	Subtropical	Oceanic	Atlantic, Pacific and Indian oceans
	* *Thunnus thynnus*	Atlantic bluefin tuna	427	Temperate	Oceanic	Atlantic Ocean
	* *Thunnus orientalis*	Pacific bluefin tuna	300	Temperate	Oceanic	Pacific Ocean
	*Thunnus tonggol*	Longtail tuna	145	Tropical	Neritic	Northern Indian Ocean, Indo-Pacific region, Western Pacific Ocean
	* *Katsuwonus pelamis*	Skipjack tuna	111	Tropical	Oceanic	Atlantic, Pacific and Indian oceans
	*Euthynnus affinis*	Kawakawa	100	Tropical	Neritic	Indian Ocean, Indo-Pacific region
	*Euthynnus alleteratus*	Little tunny	108	Tropical	Neritic	Atlantic Ocean, including the Mediterranean and Black seas
	*Euthynnus lineatus*	Black skipjack	86	Tropical	Neritic	Eastern Pacific Ocean
	*Auxis rochei*	Bullet tuna	48	Tropical	Neritic	Atlantic, Pacific and Indian oceans, including the Mediterranean Sea
	*Auxis thazard*	Frigate tuna	62	Tropical	Neritic	Atlantic, Pacific and Indian oceans
	*Allothunnus fallai*	Slender tuna	105	Temperate	Oceanic	Southern waters of the Atlantic, Pacific and Indian oceans
**Tribe Sardini (bonitos)**	*Cybiosarda elegans*	Leaping bonito	45	Tropical	Neritic, associated with coral reefs	Western Pacific Ocean restricted to the southern coast of Papua Guinea and northern Australia
	*Gymnosarda unicolor*	Dogtooth tuna	186	Tropical	Neritic	Disjoint distribution in the Indian Ocean and Indo-Pacific region
	*Orcynopsis unicolor*	Plain bonito	130	Subtropical	Neritic	Eastern Atlantic Ocean including Mediterranean Sea
	*Sarda australis*	Australian bonito	108	Subtropical	Neritic	Southwest Pacific Ocean in south western Australia and northern New Zealand
	*Sarda chiliensis*	Eastern Pacific bonito	101	Subtropical	Neritic	Eastern Pacific Ocean
	*Sarda orientalis*	Indo-Pacific bonito	102	Subtropical	Neritic	Indian and Pacific Oceans
	*Sarda sarda*	Atlantic bonito	97	Subtropical	Neritic	Atlantic Ocean including Mediterranean Sea
**Trine Scomberomorini (Spanish mackerels)**	*Acanthocybium solandri*	Wahoo	238	Tropical	Oceanic, associated with coral reefs	Atlantic, Pacific and Indian oceans, including the Mediterranean Sea
	*Scomberomorus brasiliensis*	Serra Spanish mackerel	125	Tropical	Neritic	Western Atlantic Ocean
	*Scomberomorus cavalla*	King mackerel	159	Tropical	Neritic	Western Atlantic Ocean
	*Scomberomorus commerson*	Narrow-barred king mackerel	240	Tropical	Neritic	Indian Ocean and Western Pacific Ocean. Recently found in the Mediterranean Sea along the northern African countries.
	*Scomberomorus concolor*	Monterey Spanish mackerel	77	Subtropical	Neritic	Eastern Central Pacific Ocean. Current distribution is restricted to the northern part of the Gulf of California.
	*Scomberomorus guttatus*	Indo-Pacific king mackerel	87	Tropical	Neritic, associated with estuaries	Northern Indian Ocean and Indo-Pacific region
	*Scomberomorus koreanus*	Korean seerfish	150	Tropical	Neritic	Northern Indian Ocean and northwestern Pacific Ocean
	*Scomberomorus lineolatus*	Streaked seerfish	98	Tropical	Neritic	Northern Indian Ocean and Indo-Pacific region
	*Scomberomorus maculatus*	Atlantic Spanish mackerel	80	Subtropical	Neritic	Northwestern Atlantic Ocean
	*Scomberomorus multiradiatus*	Papuan seerfish	35	Tropical	Neritic, associated with estuaries	Restricted to the Gulf of Papua and Timor Sea in the Indo-Pacific
	*Scomberomorus munroi*	Australian spotted mackerel	103	Tropical	Neritic	Indo-Pacific region restricted to northern Australia and Papua New Guinea
	*Scomberomorus niphonius*	Japanese Spanish mackerel	103	Temperate	Neritic	Northwest Pacific Ocean
	*Scomberomorus plurilineatus*	Kanadi kingfish	120	Subtropical	Neritic	Western Indian Ocean along the Eastern African Coast
	*Scomberomorus queenslandicus*	Queensland school mackerel	100	Tropical	Neritic, associated with estuaries	Indo-Pacific region restricted to northern Australia and Papua New Guinea
	*Scomberomorus regalis*	Cero	94	Tropical	Neritic	Western Atlantic Ocean
	*Scomberomorus semifasciatus*	Broad-barred king mackerel	120	Tropical	Neritic	Indo-Pacific region restricted to northern Australia and Papua New Guinea
	*Scomberomorus sierra*	Pacific sierra	99	Tropical	Neritic	Eastern Pacific Ocean
	*Scomberomorus sinensis*	Chinese seerfish	248	Subtropical	Neritic, associated with estuaries	Northwestern Pacific Ocean
	*Scomberomorus tritor*	West African Spanish mackerel	97.5	Tropical	Neritic, associated with estuaries	Eastern Atlantic Ocean including the Mediterranean Sea
	*Grammatorcynus bicarinatus*	Shark mackerel	122	Subtropical	Neritic, associated with coral reefs	Southwestern Pacific restricted to the northern coast of Australia
	*Grammatorcynus bilineatus*	Double-lined mackerel	100	Subtropical	Neritic, associated with coral reefs	Northern Indian Ocean and Indo-Pacific region with a disjoint distribution
**Tribe Scombrini (mackerels)**	*Rastrelliger brachysoma*	Short mackerel	34.5	Tropical	Neritic, associated with estuaries	Indo-Pacific region
	*Rastrelliger faughni*	Island mackerel	30.9	Tropical	Neritic	Indo-Pacific region
	*Rastrelliger kanagurta*	Indian mackerel	39.6	Tropical		Indian Ocean, Indo-Pacific region and Western Pacific Ocean
	*Scomber australasicus*	Spotted chub mackerel	55	Subtropical	Neritic	Western Pacific Ocean and northwestern Indian Ocean
	*Scomber japonicus*	Chub mackerel	63	Subtropical	Neritic	Northwest Pacific Ocean and Eastern Pacific Ocean
	*Scomber scombrus*	Atlantic mackerel	60	Temperate	Neritic	Northwest Atlantic Ocean and Northeast Atlantic Ocean including the Mediterranean Sea
	*Scomber colias*	Atlantic chub mackerel	63	Subtropical	Neritic	Eastern Atlantic Ocean including Mediterranean Sea
***Subfamily Gasterochismatinae***	*Gasterochisma melampus*	Butterfly kingfish	195	Temperate	Oceanic	Southern waters of the Atlantic, Pacific and Indian oceans

* Commonly known as principal market tuna species.

Scombrid species sustain some of the most important fisheries in the world. They support diverse commercial fisheries throughout their distributions, ranging from large-scale industrial to small-scale artisanal fisheries, and many species are caught in recreational fisheries worldwide (Table S1). Annual catches of scombrids have risen continuously since the 1950s, reaching 9.6 million tonnes in 2010 [6]. Together, all scombrid catches contribute up to 15% of the annual total marine fish catch and are worth in excess of US$ 5 billion each year [7], [8]. Scombrids are epipelagic predator and prey species and are widely distributed in coastal and oceanic waters throughout the tropical, subtropical and temperate waters of the world's oceans. The majority of the species are found in marine open waters and some are associated with estuarine and riverine habitats and coral reefs [9]. Among the fifteen species of tunas (Thunnini), seven are known as the principal market tunas due to their economic importance in the global markets (see list of species in Table 1). The principal market tunas have widespread oceanic distributions, are highly-migratory, sustain highly-industrialized fisheries worldwide and are a highly-valued international trade commodity for canning and sashimi [7], [9], [10]. The rest of scombrid species, the small tunas, bonitos, Spanish mackerels, and mackerels have in general more coastal distributions and are associated with continental shelves or oceanic islands (Table 1). While the economic value of coastal scombrids is lower in the global markets, they can reach high values locally supporting a diversity of fisheries. These are largely small-scale artisanal fisheries but also semi-industrial and industrial fisheries, in both developed and developing countries (Table S1). Hence, they are an important source of wealth and food security to local fishing communities [7], [9], [11], [12]. Given the global scale and magnitude of scombrid fisheries and their economic and social importance for many coastal countries, a global review of the life history studies of scombrids seems essential to evaluate the biological knowledge of this important group of species and set the research agenda for the coming decades.

Two recent global evaluations have provided a global picture of the current exploitation and conservation status of scombrid species. One evaluation quantified the global fishing impacts on fishery-assessed populations of scombrids showing the adult biomass of scombrids (including 26 populations of 11 of the 51 species) have decreased on average by 60% over the past fifty years [13]. It also revealed that the fisheries for the majority of these scombrid populations, mostly principal market tunas and mackerels, are currently fully exploited worldwide, suggesting that the further expansion of sustainable catches from these fisheries in the short term are limited. This study also exposed that the large majority of scombrid populations and species lack reliable and up-to-date formal quantitative stock assessments of the long-term impacts of fishing on population biomasses. Consequently, the current exploitation status remains unknown or highly uncertain for the majority of scombrid species worldwide. The other global evaluation summarized the conservation status for scombrids species using the International Union for Conservation of Nature (IUCN) Red List criteria, hence, ranking species in terms of their relative risk of global extinction [10]. Of the 51 species of scombrids, 68% (35 of 51 spp.) were listed under the Least Concern IUCN Red List category, having a relatively low risk of global extinction. Sixteen percent (8 spp.) had declined sufficiently in biomass to trigger listing under the Threatened or Near Threatened categories having relatively higher risk of global extinction. Lastly, 16% (8 spp.) of scombrids were listed under the Data Deficient category, meaning these species have insufficient information to evaluate their global conservation status. These two global evaluations together revealed that the impacts of fishing and the exploitation status for the majority of scombrid populations and species remains unknown or is highly uncertain globally and highlighted which species are in need of further protection and management. Consequently, the global life history dataset assembled and synthesized in this study will become particularly useful for those scombrid populations and species for which their exploitation and conservation status is unknown. In an era where stock assessments are expensive and data intensive and where it is unlikely that there will ever be sufficient information to develop long-term quantitative stock assessments for all exploited species, the knowledge of life history parameters can provide a starting framework in support of management [2].

In this study we first compile a data set of life history traits (maximum size, growth, longevity, maturity, spawning season and fecundity) for the 51 species of scombrids on a global scale. Second, we synthesize this life history information and critically review it to identify gaps and priorities in biological knowledge across the species. Third, we recommend and prioritize life history research needs in scombrid species based on their biological gaps in knowledge, the importance of their fisheries and their current conservation status according to the IUCN Red List of Threatened Species.

## Methods

### Data collection, search criteria and data standardization

We assembled life-history data for the 51 species of scombrids on a global scale. We specifically focused on reviewing the available growth and reproductive studies for the adult stages of the species, which are the essential information that generally feeds quantitative fisheries stock assessment models and forms the basis of their management and conservation. We assembled the life history data from a wide range of published literature including: scientific journals, reports and theses published in English, Spanish, French, Portuguese, Italian and any other language that provided an English summary.

To begin, we conducted a systematic literature review in the ISI Web of Science for each scombrid species, searching for their names (both Latin and common names) in combination with any of the following terms: growth, reproductive biology, maturity, fecundity, life history. In the search, we included all the studies up to November of 2012. We also located additional studies through the references of relevant papers obtained in the literature search; this strategy allowed us specially to locate relevant studies published as fisheries reports and theses. Additionally, we also searched for relevant literature in the web pages of several international organizations including the Food and Agricultural Organization of the United Nations, and of several Regional Fisheries Management Organizations, which regularly publish research reports on the life histories and biology of scombrid species. The Regional Fisheries Management Organizations revised included the International Commission for the Conservation of Atlantic Tunas, the Inter-American Tropical Tuna Commission, the Western and Central Pacific Fisheries Commission, the Indian Ocean Tuna Commission, the Commission for the Conservation of Southern bluefin tuna and the Caribbean Regional Fisheries Mechanism.

We reviewed all the studies identified (879 studies). We included in the data set only those original life history studies that described fully the methods employed to estimate the life history traits and excluded review and synthesis articles. By reviewing only original information, we avoided propagating widely-used but poorly-supported or erroneous parameter estimates. We ended up with 684 original life history research studies (Appendix S1) from which we extracted the following life history information:

1. Maximum length (*L_max_*, cm) of the fish observed from each life history study.
2. Growth information derived from the von Bertalanffy growth function, *L_t_ = L_∞_* (1-e^−*k*(*t*-*to*)^), where *L_t_* is the length at age *t* in years, *L_∞_* is asymptotic length in cm - the mean size the individuals in the population would reach if they were to grow indefinitely; growth coefficient *k* (year^−1^) expresses the rate at which the asymptotic length is approached and *t_o_* is defined as the hypothetical age in years that fish would have at zero length.
3. Empirical longevity or maximum observed age (*T_max_*, *years*) extracted from growth and aging studies. We distinguished between empirical longevities (*T_max_*), which are commonly estimated by authors with direct and indirect aging techniques, from theoretical longevities (*T_∞_*) which are commonly calculated using Taylor's relationship based on the von Bertalanffy growth rate parameter k, as *T_∞_* = 3/k [14]. The Taylor's longevity estimate is the age that a fish population would reach at L*_∞_*. In this study, we only considered empirical longevities (*T_max_*).
4. Length and age maturity estimates where we distinguished between length and age at first maturity (L_m_, cm; T_m_, years; which is the length and age at maturity first reached by an individual in a sample) and length and age at 50% maturity (L_m50_, cm; T_m50_, years; which is the maturity at which 50% of the individuals are matured in the sample).
5. Duration of the spawning season (Spw_season_, months).
6. Fecundity metrics including estimates of batch fecundity (absolute average batch fecundity F_average_ as the average number of oocytes across all sampled females, and relative batch fecundity, F_rel_, as the average number of oocytes per gram across all sampled females) and spawning intervals (Spw*_int_*, the average number of days between spawning events). We discuss later how we filtered fecundity studies based on the accuracy of various methodologies to estimate fecundity.

From each life history study, we extracted the trait estimates reported for females, males, and both sexes combined along with the sample sizes, the method used to estimate each of the life history traits, and the geographic extent of the study. We transformed standard lengths or total lengths into fork lengths using published length conversion equations.

### Data analysis

In this study, we first synthesize and critically review the biological knowledge on growth and reproductive biology for the 51 species of scombrids in order to identify gaps in knowledge. Then, we develop a criteria to identify and propose priorities for life history research for scombrid species.

#### Data synthesis and identification of data gaps

We synthesized and critically reviewed the biological knowledge on growth, longevity and reproductive traits including length and age at 50% maturity, fecundity, spawning duration and spawning intervals for the 51 species of scombrids. Additionally, we also reviewed the life history information for the seven species of principal market tunas at the population level (see list of species in Table 1). The principal market tunas are oceanic species with worldwide distributions, and some species are composed of various populations, with one or two populations in each ocean. Due to their widespread distributions and economic importance, the principal market tunas are managed as 23 independent management units or tuna stocks, here referred as populations, by five Regional Fisheries Management Organizations. Therefore, we reviewed the life history information and identified gaps and priorities for the 23 populations in the seven species of principal market tunas, a distinction we deemed relevant given the scale of their management. We used standard plots for basic descriptive statistics to synthesize the life history information assembled. In all the figures and analyses, we preferentially used the female estimates whenever the traits were reported separately for sexes in the studies.

We divided the synthesis of life history traits into two main sections: (1) growth and longevity and (2) reproductive biology. In the first section, we synthesized the growth and longevity estimates available by counting the number of von Bertalanffy growth curves available for each scombrid species, and by examining the von Bertalanffy growth parameters and empirical longevity estimates within and across all scombrid species. We also described the growth patterns across scombrids and compared them to the rest of marine fishes. To do this we extracted all the von Bertalanffy growth curves available for all marine fishes from FishBase [5]. While the von Bertalanffy growth parameters, *L_∞_* and *k*, are fundamental to describe the growth trajectories of individual species, it is not straight-forward to use *L_∞_*, which represents size, and *k*, which has time dimensions (y^−1^), by themselves to compare multiple growth curves and growth rates across multiple species [15]. Instead, a metric linking change in size or weight of a species with time is needed to describe growth patterns across multiple species [16]. Therefore, we used two complementary approaches to describe the growth patterns in scombrid species. First, we used the von Bertalanffy *k* parameter, which conveys how fast a species reaches its maximum body size to differentiate between “fast growing” and “slow-growing” species given a maximum body size. Second, we used the growth performance index, initially developed by Pauly 1979, and defined as Ø′ = log_10_ *k*+2log_10_ *L_∞_*, which is a metric with dimensions of size and time, to differentiate between species that have “high growth performances” from species having “low growth performances” regardless of their maximum body size [17]. A species with a high index of growth performance would rapidly reach a large maximum body size in a short time span and therefore would have both relatively high *k* and *L_∞_* values compared to species with low growth performances. However, because the growth performance index is the product of combining information from two parameters, *L_∞_* and *k*, a high index of growth performance could also be the result of having only a high *L_∞_*, thus, it does not necessarily imply fast growth rates (a high *k*) to reach *L_∞_*. Yet, the species with the highest growth performances will have both relatively high *L_∞_* and *k*. Moreover, we also used the growth performance index Ø′ to highlight potentially inaccurate growth curves. Given that the Ø′ values for a given species, or a taxonomically related group of species, is expected to be normally distributed around the mean Ø′ of the taxonomic unit, values further away from the mean of the distribution must be interpreted with increasing caution [17]. In addition, we also used an auximetric plot, which is a double logarithmic plot of the parameters *k* and *L_∞_* [15], [18] to portray and visualize “fast vs slow growing” scombrid species given a maximum body size and species with “high vs low growth performances”, and visualize how the growth space of scombrid species compare with the rest of marine fishes. By plotting *k* vs *L_∞_*, which are inversely related, in the auximetric plot, the growth space utilized by fishes can be represented [15], [16]. Different population of a same species will tend to form a cluster of points, describing the “growth space” of the species, and the cluster of points will grow in size as higher taxonomic levels (e.g. genera and families) are included in the plot.

In the second section, we synthesized all the reproductive studies by counting all the maturity and fecundity studies available for each scombrid species, and by examining the maturity and fecundity estimates within and across species. We first present an overview of the maturity studies in scombrids by examining the available estimates of length and age at 50% maturity and relative ratios (length and age at maturity divided by maximum size) within species and across species. These ratios describe the differences among species in somatic and reproductive investments and they can also be used to highlight potential inaccurate estimates within each species. Then, we provide an overview of the fecundity studies. Understanding fecundity in scombrid fishes is challenging because they are batch spawners, spawning multiple times during the spawning season and have indeterminate fecundity. Indeterminate fecundity refers to species whose annual potential fecundity is not fixed before the spawning season, since unyolked oocytes continue to be produced, matured and spawned during the spawning season. In contrast, determinate fecundity refers to species for which annual potential fecundity is fixed before the spawning season [19]. In order to estimate the potential annual fecundities of scombrids, three measurements are required: batch fecundity (number of eggs released per spawning), spawning frequency (number of days between spawning events), and the duration of the spawning season [19]–[21]. In addition, the ovaries of all scombrid species are considered asynchronous, meaning that oocytes of all stages of development are present in the ovary simultaneously without a distinctive oocyte size class [19], [21]. This is characteristic of species with protracted spawning season, where oocyte development depends on the food available in the environment [19]. Therefore, histological analysis of ovarian tissue is needed to accurately measure batch fecundity in scombrids since there is a critical moment along all the stages of oocyte maturation when batch fecundity can be estimated [21]. At the final stages of oocyte maturation, beginning with migratory-nucleus phase and followed by hydration, which results in a clear hiatus or size break along the distribution of ooyctes, batch fecundity can be derived by counting the number of hydrated oocytes in ovaries. While a more detailed description on the methods to derive accurate batch fecundities in scombrid species can be found in Schaefer *et* al. (2001) and Murua and Saborido-Rey (2003), what we need to know here is that only ripe, pre-spawning females, with hydrated oocytes in their ovaries can be used to estimate batch fecundity accurately by means of histological analysis. Therefore, we reviewed all fecundity studies (134 studies) and selected only those studies using accurate methodologies that clearly stated that the species studied was identified as batch spawner, had indeterminate fecundity, reported asynchronous development of oocytes in the ovaries, used histological analysis, and estimated batch fecundity based on the count of the number of migratory-nucleus or hydrated oocytes [21]. Our criteria led to a selection of 33 fecundity studies, which we used to examine batch fecundities and spawning frequency within and across scombrid species. Unfortunately most of the fecundity studies of scombrid species conducted in the last 50 years used inaccurate methodologies, for example, by wrongly assuming determinate fecundity or overestimating fecundity by counting oocytes before the hydration stage. This concern was already raised by Schaefer *et* al. (2001), which reviewed the reproductive biology studies of tunas, but those concerns can be further extended to all scombrid species. Furthermore, we had to exclude from our analysis the majority of fecundity studies for Atlantic mackerel (*Scomber scombrus*) for which total annual fecundity, instead of batch fecundity, is routinely estimated, given that this species is managed in the Northeast Atlantic Ocean under the assumption of a determinate fecundity pattern. The accuracy of this assumption is, however, being revised [22], [23]. Finally, we also examined the extent of the spawning season in scombrid species within major climates (tropical, subtropical and temperate) (see species climate region in Table 1).

#### Criteria for setting life history research priorities

We identified and proposed a set of priorities for life history research for the 51 species of scombrids based on the following criteria: (1) their biological life history data gaps, (2) the importance of their fisheries throughout their distributions and (3) their current conservation status according to the IUCN Red List of Threatened species. Using our life history synthesis, we differentiated between life history data-rich species, species having life history studies on growth, maturity and fecundity, from life history data-poor species, those lacking information on either growth, maturity or fecundity. We also summarized the main fisheries of each scombrid species (Table S1) and used this information to differentiate between species that are commercially targeted and non-targeted by fisheries. Finally, we differentiated between species listed as Threatened, Near Threatened and Data Deficient from those listed as Least Concern in the IUCN Red List [10]. Threatened species are those listed as Critically Endangered, Endangered, and Vulnerable in the IUCN Red List [24]. Species in the Data Deficient category are species for which there is insufficient information to evaluate their risk of extinction, and they may or may not be Threatened. We constructed a Venn Diagram to illustrate all possible logical relations between our three main criteria. We assigned the highest priority rank for life history research to those species that: (1) are life history-data poor, (2) are targeted by commercial fisheries, and (3) are listed as Threatened, or Near Threatened or Data Deficient on the IUCN Red list. Because of the risk associated with the uncertainty in their status, we treated Data Deficient species together with species in the Near Threatened and Threatened categories as high priority in life history research. Similarly, we assigned the second highest priority to those species that are life history data-poor and are also targeted by commercial fisheries throughout their ranges.

All data management, manipulation and plots were done using the R statistical software, v.2.14.2 [25] and the packages ggplot2 [26] and VennDiagram [27]. The life history data set is available upon request from the corresponding author.

## Results and Discussion

Below we first present the global synthesis and review on the biological knowledge on growth and reproductive traits for the 51 species of scombrids and 23 populations of principal market tunas. Then, we propose priorities for life history research for scombrid species based on their biological gaps in knowledge, the importance of their fisheries throughout their distributions and their current conservation status according to the IUCN Red List of Threatened Species.

### Biology of scombrids: Current knowledge, data gaps and data concerns

#### Growth and longevity

**Figure 2:**
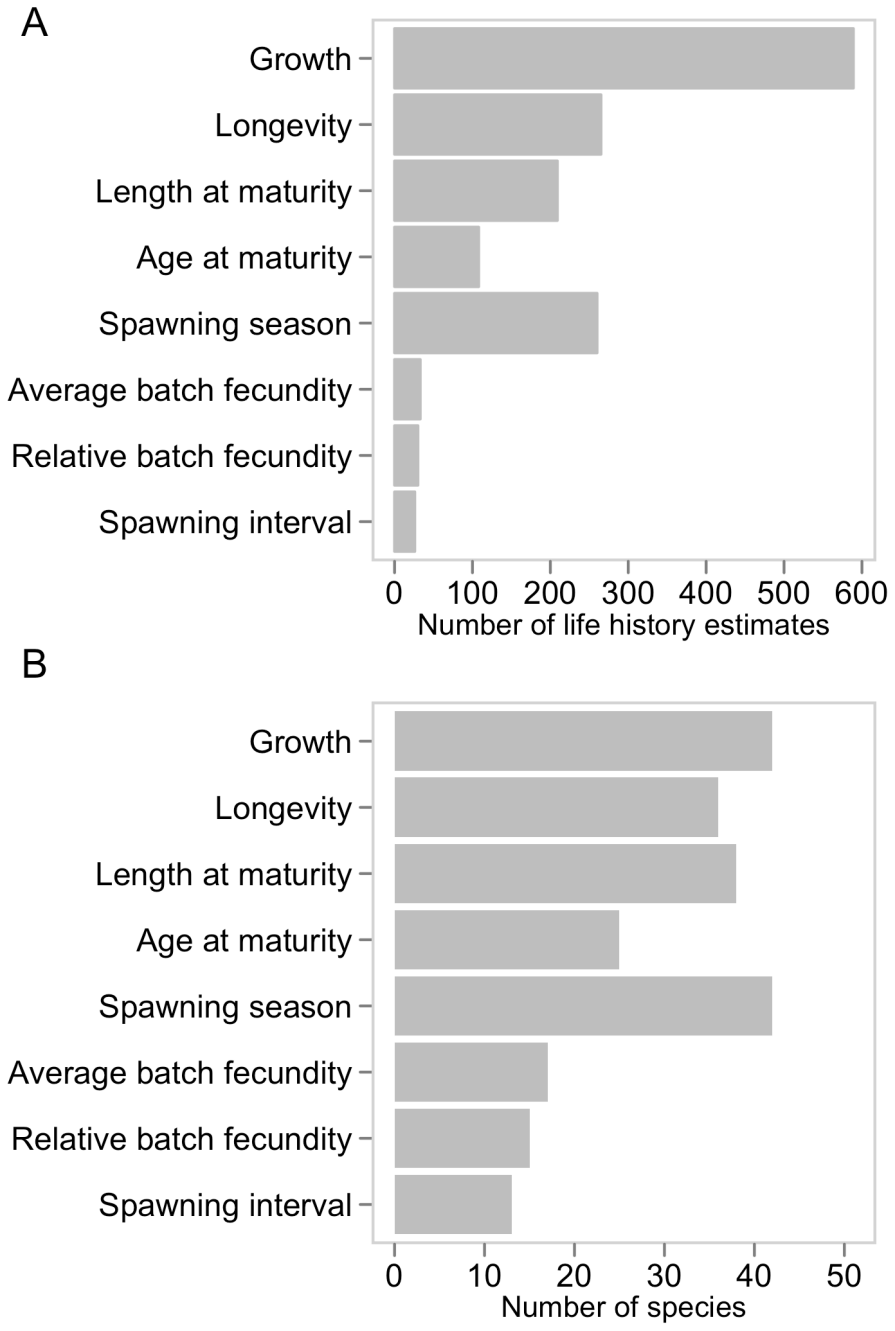
Life history information in scombrid species. Information includes life history estimates of von Bertalanfy growth parameters, longevity, length and age at 50% maturity, duration of spawning season, average batch fecundity, relative batch fecundity and spawning interval. (A) Number of life history trait estimates in the dataset for all the species combined. (B) Number of scombrid species with at least one life history trait estimate. There are 51 species in the family Scombridae.

**Figure 3:**
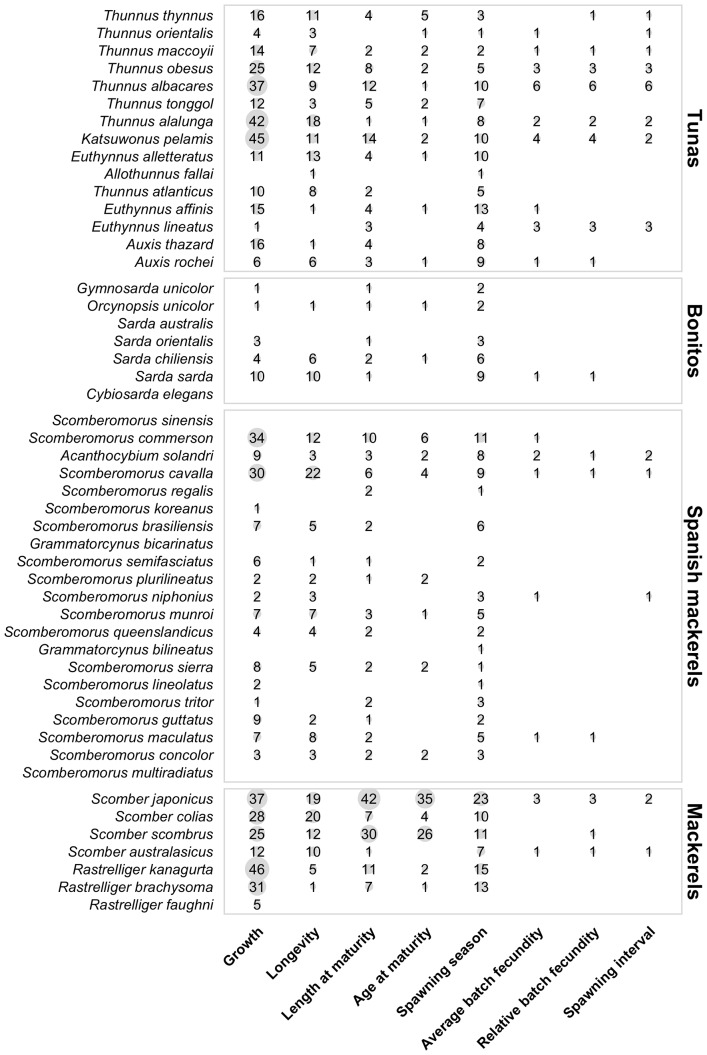
Synthesis of life history information in scombrid species. Number of estimates for each life history trait within the main four taxonomic groups of scombrids (tunas, bonitos, Spanish mackerels and mackerels). Within each taxonomic group, the species are plotted in ascending rank order of body size, with the smallest species at the bottom (See Table 1 for maximum body size). The Butterfly kingfish (*Gasterochisma melampus*), the only species in the subfamily Gasterochismatinae, is not included. The only life history trait recorded for this species is maximum length, being 195 cm [59]. The area of the grey circles is proportional to the number of estimates available for each trait.

**Figure 4:**
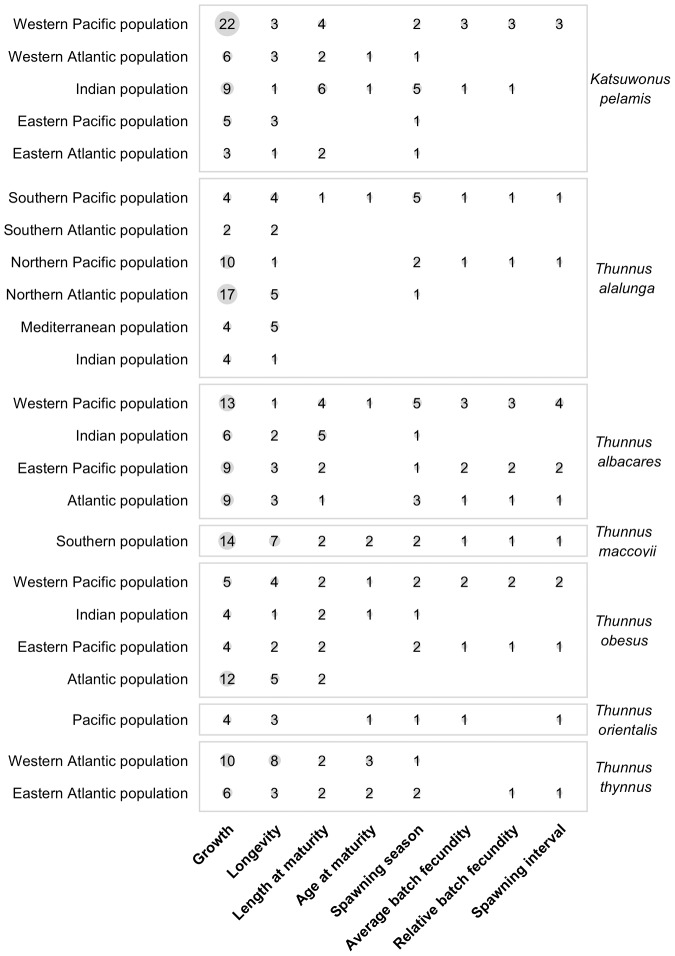
Synthesis of life history information in principal market tuna species. Number of estimates for each life history trait in the 23 populations of seven principal market tuna species. The area of the grey circles is proportional to the number of estimates available for each trait.

**Figure 5:**
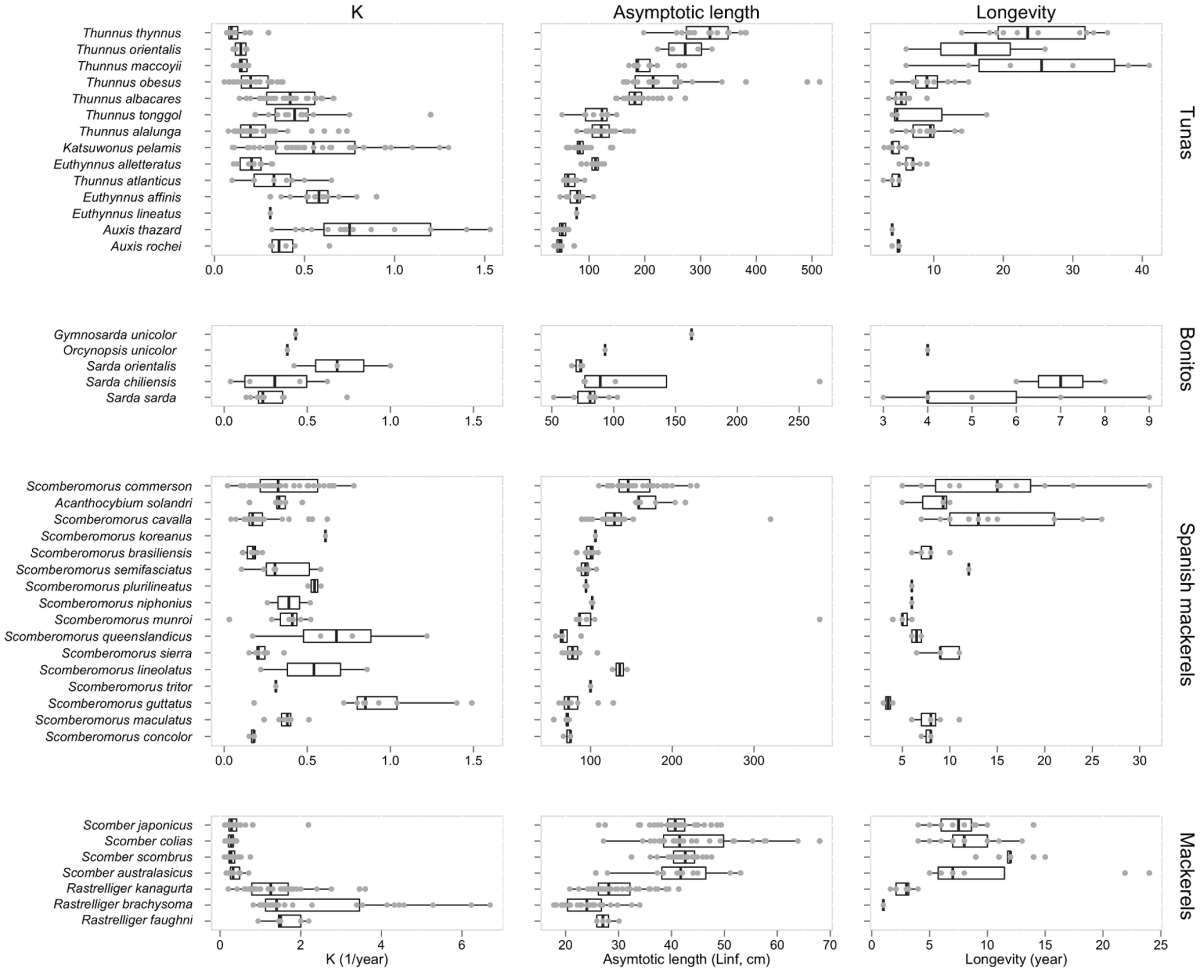
Von Bertalanffy growth parameters – growth rate *k* (y^−1^) and asymptotic length *L_∞_* (cm), and longevity estimates in scombrid species. Within each taxonomic group, the species are plotted in ascending rank order of body size, with the smallest species at the bottom (See Table 1 for maximum body size).

**Figure 6:**
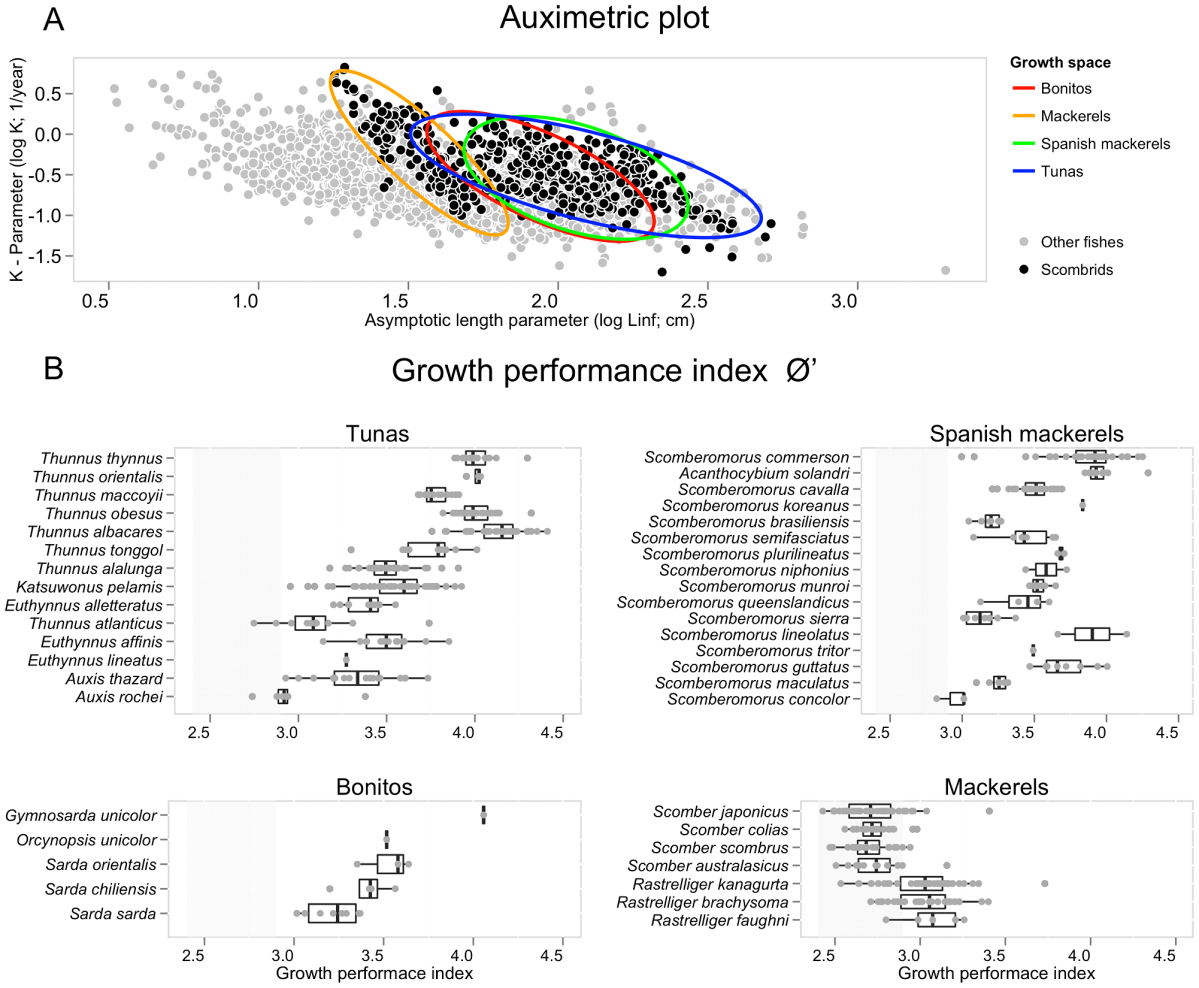
Growth performances in scombrid species including all fishes in FishBase, illustrating the high growth performances of scombrids. (A) Auximetric plot comparing the growth performance of scombrid species (black circles) with that of fishes in general (grey circles). Fish data extracted from FishBase as August 2012. The growth space for the main four taxonomic groups of scombrids, tunas, bonitos, Spanish mackerels and mackerels, are also illustrated (colored ellipse curves). (B) The growth performance index Ø′ (defined as Ø′ = log_10_ *k*+2log_10_ *L_∞_*) of scombrid fishes compared with the average growth performances in fishes in FishBase (average growth performance is 2.7±0.3, grey band area).

There are a total of 547 von Bertalanffy growth curves in the data set and growth has been studied in 42 of 51 species of scombrids (Figure 2 and 3) and in all 23 populations of principal market tunas (Figure 4). The *L_∞_* and *k* coefficients vary greatly between scombrid species ranging from 24.4 cm and 2.3 y^−1^, respectively, in the short mackerel (*Rastrelliger brachysoma*) to 309.7 cm and 0.12 y^−1^, respectively, in the Atlantic bluefin tuna (*Thunnus thynnus*) (Figure 5). Scombrids are among the fastest growing species of all fishes with relatively high *k* values (a mean *k* of 0.48 y^−1^) given their maximum size when compared to the rest of fish species, exhibiting rapid growth toward their maximum body size (Figure 6A). Among all scombrid species, the fastest growing species (*k* values>0.7 y^−1^) given their maximum body size are the three tropical Indian mackerels, Indian mackerel (*Rastrelliger brachysoma*), Island mackerel (*R. faughni*) and short mackerel (*R. kanagurta*), the Indo-Pacific king mackerel (*Scomberomorus guttatus*) and frigate tuna (*Auxis thazard*) (Figure 5). Moreover, the three bluefin tuna species (*Thunnus thynnus*, *T orientalis*, and *T. maccoyii*) and two Spanish mackerels (Monterey Spanish mackerel *Scomberomorus concolor* and Serra Spanish mackerel *S. brasiliensis*) are the slowest growing species (*k*<0.2 y^−1^) among scombrids.

The growth performance index Ø′ in scombrids is among the highest in fish species, indicating not only that scombrids have relatively high *k* values given their maximum body size, but they also have both relatively high *k* and *L_∞_* values, being able to grow very fast to large body sizes compared to the rest of fish species (Figure 6). Note how the growth space of scombrids is located towards the top right quarter of the auximetric plot, although there are some exceptions (Figure 6A). Four tuna species, yellowfin, Atlantic bluefin, Pacific bluefin and bigeye tuna (*Thunnus albacares*, *T. thynnus*, *T. orientalis* and *T. obesus*, respectively) have the largest growth performances indices (Ø′>4) among scombrids, followed by dogtooth tuna (*Gymnosarda unicolor*), wahoo (*Acanthocybium solandri*) and the narrow-barred Spanish mackerel (*Scomberomorus commerson*) (Figure 6B). On the other hand, the four temperate mackerel species, chub mackerel (*Scomber japonicus*), Atlantic mackerel (*S. scombrus*), Atlantic chub mackerel (*S. colia*s) and blue mackerel (*S. australasicus*) have the lowest growth performances among scombrid species (Ø′<2.7). Yet, scombrid species have among the highest growth performances of all fishes with an average Ø′ values of 3.4, while the average Ø′ for the rest of marine fishes is 2.7 (Figure 6B). What explains the high growth rates and high performances of scombrid species? Pauly's theory of growth in fishes states that the oxygen supply, and therefore the gill surface area, is the limiting factor of growth in fishes [15], [16], [28]. The gill structure of scombrids is among the most advanced in fishes. All scombrid species have disproportionally large gill surface areas relative to their body weights, and tuna species have the largest gill surface areas among all scombrids, permitting high rates of the oxygen acquisition to maintain those high rates of growth [29]. Pauly's work on growth in fishes starting in the 1980s already noticed that tuna species had relatively high growth rates and large gill sizes compared with the rest of teleost fishes, directing him to investigate the positive relationship between that gill surface area of fishes, hence supply of oxygen, and their maximum growth rates [15], [28].

Longevity is a difficult parameter to estimate in fishes, as it depends on the accuracy of the growth-determination methods and the age-validation techniques, but it is an essential parameter to consider when managing exploited populations [30], [31]. Longevity estimates were available for 36 of the 51 species of scombrids (Figure 2 and 3) and in all 23 populations of principal market tunas (Figure 4). We find that the average longevity across all scombrid species is 12.2 years, making scombrids medium-lived species when compared to the rest of fishes, according to the life history productivity classification of the American Fisheries Society [32]. However, longevity estimates vary greatly within scombrid species (Figure 5). On one extreme, the shortest-lived tropical mackerels (short mackerel *Rastrelliger brachysoma* and Indian mackerel *R. kanagurta*) have longevities of 1 and 4 years, respectively. On the other extreme, the southern bluefin tuna (*Thunnus maccoyii*) with a maximum estimated longevity of 41 years, Atlantic bluefin tuna (*T. thynnus*) (35 years) and narrow-barred Spanish mackerel (*Scomberomorus commerson*) (31 years) are the longest-lived species of scombrids.

Growth and longevity have not been studied at all in nine and fifteen scombrid species, respectively. In addition, we find that the estimates of the von Bertalanffy growth parameters, *L_∞_* and *k*, the growth performance index Ø′, and longevity vary substantially within some scombrid species (Figure 5 and 6). This variation can be attributed mainly to two factors: (1) the life histories of species may vary with average temperature and seasonality at different latitudes within their distributions [33], and (2) the accuracy of the aging and growth approaches used, and the power of the validation methods employed, if any [30]. The von Bertalanffy growth curves of scombrids were estimated using a variety of aging methods including direct methods such as calcified structures (vertebrae, spines, scales and otoliths) and indirect methods such as modal analysis of length frequencies and tagging studies, or by various combinations of several of these methods (Figure S1). While it is not the objective of this study to quantify how much variation in growth might be due to environmentally-driven intraspecific variability within species and how much by differences in aging techniques, we compared estimates of *k*, the growth performance index Ø′ and longevity between several aging techniques, and observed that some of the differences can be attributed to the ageing approaches employed (Figure S2, S3 and S4). This type of analysis should ideally be carried out at the species level, to better determine the effect of different aging techniques on growth estimates and to identify what methods are more consistent leading to more accurate age and growth estimates for each species. Moreover, while we cannot disentangle easily the effect of aging techniques on growth and age estimations, we can easily use the growth performance index Ø′ to identify potential inaccurate growth curves for each individual species. The Ø′ values for a given species or taxonomically related group of species is expected to be normally distributed around the mean Ø′ of the taxonomic unit [17]. Therefore, we advise to interpret with increasing caution the growth curves with Ø′ values the further away from the mean of their distribution. We consider the scombrid growth curves depicted as outliers in the boxplots in figure 6B potentially unreliable and we advise against their use.

#### Reproductive biology

**Figure 7:**
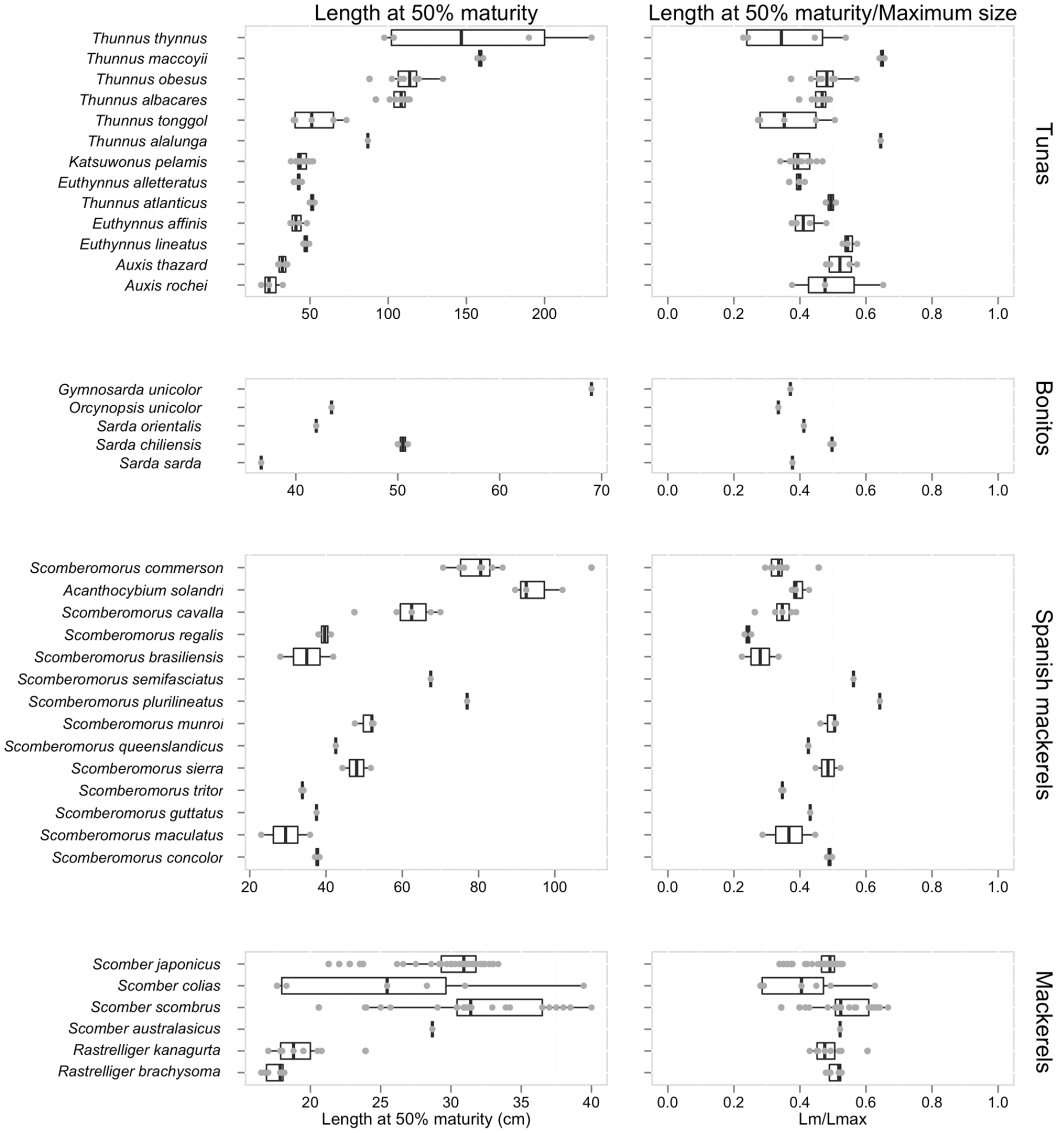
Length at 50% maturity estimates and the ratio length at 50% maturity/maximum body size for scombrid species. Within each taxonomic group, the species are plotted in ascending rank order of body size, with the smallest species at the bottom (See Table 1 for maximum body size).

**Figure 8:**
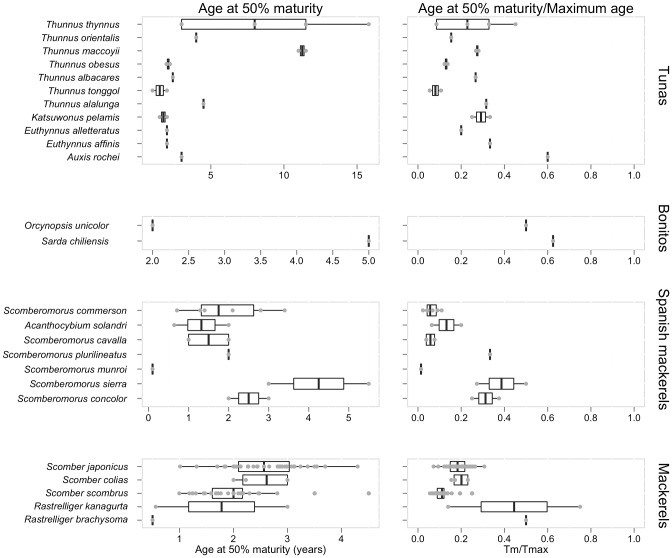
Age at 50% maturity estimates and the ratio age at 50% maturity/maximum body size for scombrid species. Within each taxonomic group, the species are plotted in ascending rank order of body size, with the smallest species at the bottom (See Table 1 for maximum body size).

We first present an overview of the maturity studies in scombrids followed by an overview of the fecundity studies. The length at which 50% of the sampled individuals have matured, were available for 38 of the 51 species (Figure 2 and 3) and 16 of the 23 principal market tuna populations (Figure 4). While at first we observe that small scombrid species tend to mature at smaller sizes than larger-bodied scombrids, we also find that scombrids reach maturity at similar proportional sizes, at around half of their maximum length, typically at 44.7% of the maximum length (Figure 7). Multiple studies have documented the relative constancy of the ratio L_m50_/L_max_ within most families of fish and other taxonomic groups [1], [34]. Yet, it has also been documented that smaller species tend to reach maturity at larger sizes relative to their maximum body sizes while larger species tend to mature at relatively smaller sizes. This pattern can also be discerned in Figure 7 where, for example, the smallest scombrid for which maturity information exists, the short mackerel (*Rastrelliger brachysoma*), matures at 16.7 cm (at 50% of its maximum body size) and the largest scombrid, the Atlantic bluefin tuna (*Thunnus thynnus*), matures at 155.2 cm (at 36% of its maximum body size, combining information for both eastern and western population). Moreover, we find that estimations of age at first maturity are scarcer in scombrid species (Figure 2 and 3). Reproductive studies only estimated age at 50% maturity or reported age at 50% maturity by converting length at maturity to age using a Von Bertalanffy growth equation for 25 species of scombrids (Figure 8). With the limited information available, we find that scombrids appear to mature early in life compared to their maximum life span, at around one quarter of their lifespan (at 25.4% of the maximum age across all the species) (Figure 8). Extreme values in the distribution are provided by Australian spotted mackerel (*Scomberomorus munroi*), which matures at 0.3 years (at 5% of the maximum age), while southern bluefin tuna (*Thunnus maccoyii*) reaches maturity at 11 years old (at 27% of the maximum age).

**Figure 9:**
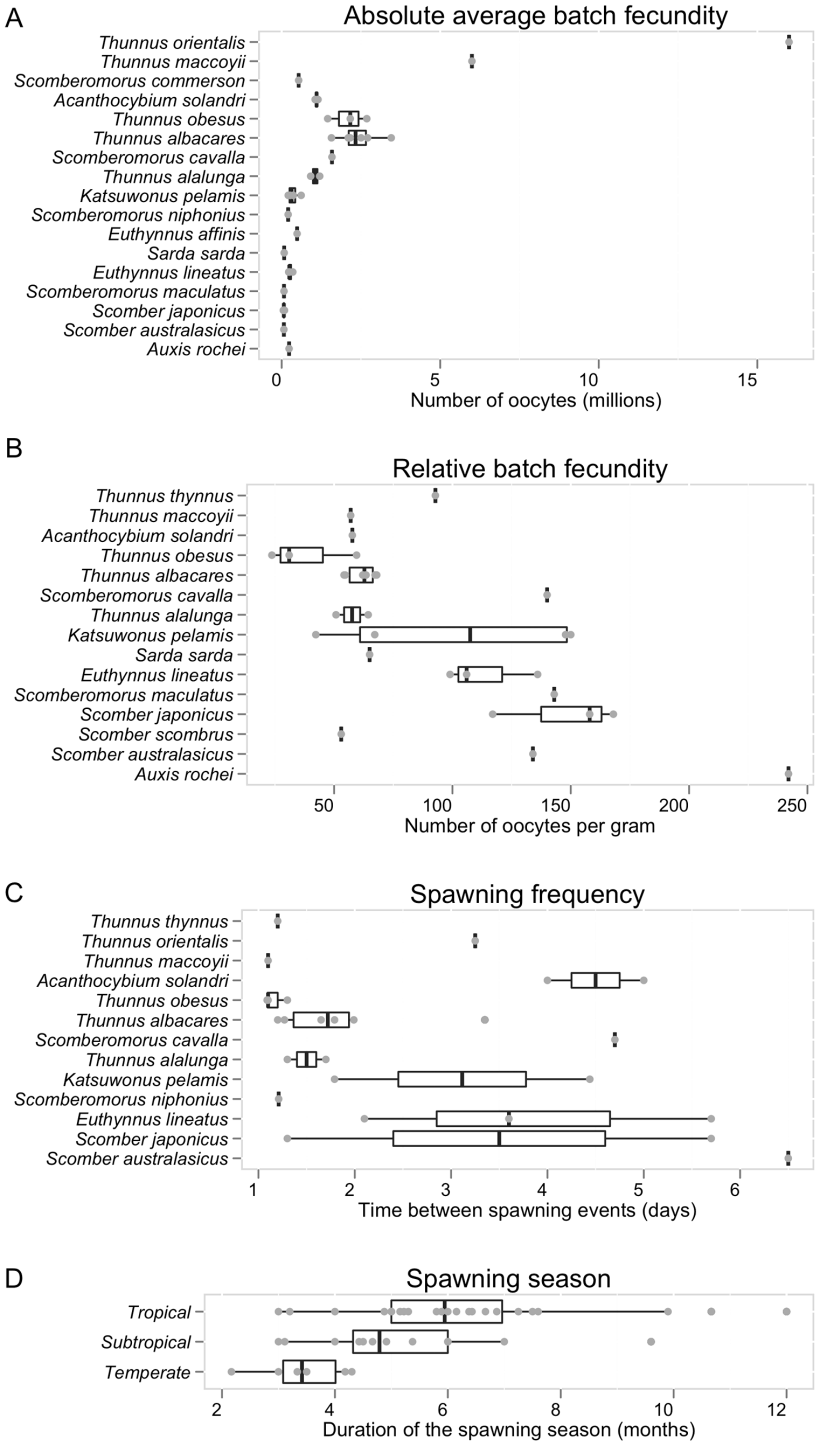
Batch fecundity estimates for scombrid species. (A) Absolute average batch fecundity. (B) Relative average batch fecundity. (C) Duration of spawning season of scombrid species by type of climate (find species climate in Table 1). In all the figures, the species are plotted in ascending rank order of body size, with the smallest species at the bottom (See Table 1 for maximum body size).

Absolute average batch fecundities, relative batch fecundities and spawning frequencies were available for 17, 15 and 13 species of scombrids, respectively (Figure 2 and 3). Estimates of average absolute batch fecundities vary greatly across scombrid species, ranging from 69,000 oocytes in blue mackerel (*Scomber australasicus*) to 16 million eggs in Pacific bluefin tuna (*Thunnus orientalis*), which is mainly driven by the different body sizes of the species (Figure 9A). The average relative batch fecundity (number of oocytes per gram) is a better metric to compare fecundity among species of different sizes. The number of oocytes per gram in scombrids ranges from 38 in bigeye tuna (*Thunnus obesus*) to 242 in bullet tuna (*Auxis rochei). S*maller scombrids tend to have higher mass-specific fecundities, spawning a greater number of oocytes per gram of body mass than bigger scombrid species (Figure 9B). The time between successive spawning events in scombrid species varies between every 1.1 days in southern bluefin tuna (*Thunnus maccoyii*) to every 6.5 days in blue mackerel (*Scomber australasicus*), and smaller scombrids tend to have greater intervals between spawning events than larger body scombrids, although there are more some exceptions (Figure 9C). Finally, tropical species have generally longer spawning seasons (an average of 6 months), than their subtropical (5 months) and temperate (3.5 months) relatives (Figure 9D), suggesting an association between spawning duration and the type of environment that species inhabit.

Information on the full reproductive biology, including length and age at 50% maturity, batch fecundities, spawning duration and frequency, is incomplete for most scombrid species (Figure 2 and 3), and around half of the populations of the principal market tunas (Figure 4). The length at 50% maturity and spawning season is unknown for 13 and 9 species of scombrids, respectively. More worrying, accurate fecundity studies are lacking for 34 of the 51 species of scombrids. We also find that estimates of length at 50% maturity are less variable than growth estimates, suggesting that there is more uniformity among the methods (Figure 7). However, some species show large variability among studies, calling for some detailed examinations. Given the relative constancy of the ratio L_m50_/L_max_ within scombrid species, we find this ratio particularly useful to identify those species and studies that need further examination. For example, the estimates of length at 50% maturity and the ratio L_m50_/L_max_ vary greatly among studies for the species Atlantic bluefin tuna (*Thunnus thynnus*), longtail tuna (*T. tonggol*) and Atlantic chub mackerel (*Scomber colias*). In the case of Atlantic bluefin tuna (*T. thynnus*), the different lengths at maturity of the eastern and western Atlantic populations might be driving some of the observed variation. It has been hypothesized that the different histories of exploitation for the two populations might explain some of the differences (ICCAT, 2009). The large differences in length at 50% maturity for longtail tuna (*Thunnus tonggol*) and Atlantic chub mackerel (*Scomber colias*) could be driven by the different methodologies employed in the studies or perhaps be an environmental-driven response of the species within its distribution. Finally, we also see some discrepancies in the estimates of relative batch fecundity within some species, for example the relative fecundities of skipjack tuna (*Katsuwonus pelamis*) differ greatly among studies.

### Setting priorities in life history research and future directions

**Figure 10:**
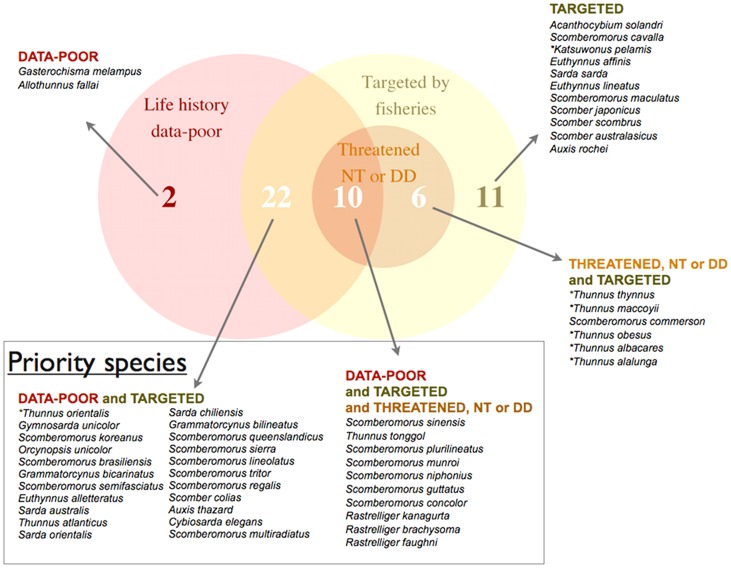
Venn Diagram of life history research priorities in scombrid species. We differentiated between life history data-poor and data-rich species (see definition in main text), between species targeted and non-targeted by commercial fisheries (see Table S1), and between species listed as Threatened, Near Threatened (NT) and Data Deficient (DD) from those listed as Least Concern in the IUCN Red List (See Table S2). Threatened species are those listed as Critically Endangered, Endangered, and Vulnerable in the IUCN Red List. Enclosed box illustrates scombrid species with the highest priorities for life history research. (*) Highlights the principal market tuna species.

Although we would ideally encourage a large range of biological studies to fill all life history data gaps of scombrid species identified in this study (Figure 3 and 4), we propose instead a set of priorities for research based on the following criteria: (1) their biological life history data gaps distinguishing between data-rich and data-poor species, (2) the importance of their fisheries distinguishing between commercially target and non-target species, and (3) their current conservation status according to the IUCN Red List, distinguishing between species listed as Threatened, Near Threatened and Data Deficient from those listed as Least Concern (Figure 10). We find one-third of scombrid species (17 spp.) are life history data-rich species, as they have reasonable information on growth, maturity and fecundity (Figure 3, Table S2). Half of the species (26 spp.) lack information on either growth, maturity or fecundity, and eight species have no information at all on growth, maturity or fecundity, for which we know little more than their maximum body sizes and their overall distributions; we refer to them as data-poor species (Figure 3, Table S2). We also find that all scombrid species are targeted by commercial fisheries, except only for two of them: Butterfly kingfish (*Gasterochisma melampus*) and slender tuna (*Allothunnus fallai*) (Table S1). Finally, 35 of the 51 species of scombrids are listed under the Least Concern IUCN Red List category, 8 species under the Threatened or Near Threatened categories and 8 species of scombrids are listed under the Data Deficient category [10].

Based on our criteria we identified two groups of species for which life history research should be prioritized in the coming decades (Figure 10). The first priority group is made up of ten scombrid species for which we identified large life history-data gaps, are currently targeted by commercial fisheries throughout their distributions and are listed as Threatened, Near Threatened or Data Deficient in the IUCN Red List (Figure 10). These species include six Spanish mackerels (*Scomberomorus sinensis*, *S. plurilineatus*, *S. munroi*, *S. niphonius*, *S. guttatus*, *S. concolor*), one tuna (*Thunnus tonggol*) and the three tropical mackerels (*Rastrelliger kanagurta*, *R. brachysoma and R. faughni*). The full reproductive biology of these species is unknown or very poorly known. This is particularly relevant for Chinese seerfish (*Scomberomorus sinensis*) and Japanese Spanish mackerel (*S. niphonius*), two important commercial species off the coast of Japan, Korea and China [35]–[38]. While for Japanese Spanish mackerel we lack any length or age at maturity and fecundity estimates, for Chinese seerfish there is no data on maturity, fecundity or growth. Given the large maximum size reported for these species (Chinese seerfish ∼240 cm and Japanese Spanish mackerel ∼103 cm), it is likely they might be vulnerable to fishing pressure throughout its range. Moreover, all the species in our top priority list were categorized as Data Deficient, with the exceptions of Monterey Spanish mackerel (*Scomberomorus concolor*) and Australian spotted mackerel (*S. munroi*) which were listed as Vulnerable [10]. For these Data Deficient species there is insufficient data on their biology, population status, and current threats to even conduct the IUCN assessments, yet, they sustain diverse commercial fisheries in many countries throughout their ranges (Table S1). Exacerbating the effect of poor biological knowledge of these species and unknown exploitation and conservation status, the landings of these species have increased greatly in the last decade, but are usually misclassified and highly underreported throughout their ranges [6], [7], [10].

The second group of species for which life history research should be prioritized is made up of twenty-two data-poor and commercially-targeted species of scombrids (Figure 10). For five of them (shark mackerel *Grammatorcynus bicarinatus*, double-lined mackerel *G. bilineatus*, *Australian bonito Sarda australis*, leaping bonito *Cybiosarda elegans*, Papuan seerfish *Scomberomorus multiradiatus*), we know little more than their maximum size and their overall distributions. All those twenty-two species are currently supporting diverse commercial fisheries throughout their distributions (Table S1), yet most of them lack either proper quantitative fisheries stock assessments, or those available are outdated, and therefore their exploitation status is unknown or poorly known throughout their distributions [10], [13]. Similar to the species in the first priority group, landings for these scombrid species have been increasing greatly in the last decades, but they are often misclassified and underreported in the different fisheries statistics [6]. For all these reasons, we stress life history research should be prioritized on these two groups of species following the species-specific life history gaps identified in this study (see life history data gaps in Figure 3) in the coming decades. Basic life history knowledge on growth, maturity and fecundity schedules has proven to be very valuable in fishery data poor situations. Several methods have been developed with the aim to support the management of species with a lack of long term fisheries statistics based on basic life history information of the species, which have proved useful to rank species according to their intrinsic sensitivities to threats such as fishing [39]–[42]. These methods are now commonly used to identify and select sensitive species to prioritize management and efforts to protect and recover most threatened species. Finally, we also observe that most of the species in the two priority groups are endemic in the Indian Ocean and Indo-Pacific region, which we identified as the region with the highest diversity of scombrid species, and the region with the largest number of data-poor scombrid species.

Only one species of principal market tunas, the Pacific bluefin tuna (*Thunnus orientalis*), was included in the priority species list. However, the life history review for the 23 principal market tuna populations revealed that there are multiple tuna populations for which the reproductive biology, including length and age at maturity and fecundity schedules, is still poorly known (Figure 4). Particularly, the reproductive biology of albacore tuna (*Thunnus alalunga*) and Pacific bluefin tuna (*T. orientalis*) appear to be poorly known when compared with other principal market tunas. This is remarkable given the economic importance of these species globally [7], [13]. It is noteworthy that only recently the first complete studies on the reproductive biology of north Pacific albacore tuna, south Pacific albacore tuna and Pacific bluefin tuna were published [43]–[45]. Although several old studies reporting estimates of length at first maturity instead of length at 50% maturity exists for these species, we believe these estimates must be used with caution, and we do not report them here, because they are highly variable and might not represent length at maturity for the populations as a whole [21]. Therefore, we recommend prioritizing research on the reproductive biology including maturity and fecundity studies for Pacific bluefin tuna, and populations of Albacore tuna, other than the northern and southern Pacific populations.

Up to now, we have focused on identifying life history research priorities for specific species of scombrids. Yet, the determination of longevity and validation studies of age is one area of life history research that we believe should also be given high priority in the coming decades. Biological timings and rates, such as maximum age, age at maturation and growth rate are one of the primary axes of life history variation in vertebrates and especially scombrids [46]. The ability to accurately estimate and validate age in fishes is important for the subsequent estimation of demographic parameters of growth, mortality, longevity and age at maturity [30], [31]. In light of the within-species variation observed, longevity estimates in scombrids should be used with caution and we recommend prioritizing age validation studies particularly for long-lived scombrids. To date, age validation techniques have only been applied recently to some populations and species of the genera *Thunnus*, *Scomber*, and *Scomberomorus* [43], [47]–[51]. The ages and longevities of large-bodied, and potentially long-lived, species have often been underestimated in fishes, potentially causing fisheries management plans to be less successful [31]. Scombrid species include some of the most valuable exploited species in the world and the sustainability benefit of valid demographic estimates would seem worth the comparatively modest outlay involved in age validation.

Finally, we highlight some of the caveats of this study and suggest future directions to address them. First, by synthesizing and identifying life history research priorities at the taxonomic unit of species, we overlooked scombrid species that have widespread distributions and therefore the possibility of multiple locally-adapted populations throughout their geographical range. While it was not the scope of the present study to review and prioritize life history research in scombrids at the population level, in part due to the large volume of work and time constraints, the population structure for the large majority of scombrids species is unknown or poorly known throughout their distributions, with very few exceptions. Second, by focusing this study on the taxonomic unit of species we also overlooked the potential spatial and temporal patterns of life history variation within each scombrid species or populations. Life history traits for a given species might vary spatially in response to environmental effects and latitudinal clines [33], [52] and in addition vary temporally in response to fishing-induced effects [53]. To our knowledge, very few studies have quantified how growth and reproductive life history traits vary spatially within the species distributions [45], [54], [55] or vary temporally perhaps induced by fisheries exploitation [56], [57]. We therefore further encourage two broad lines of research. An immediate line of research with relatively low cost could make use of the life history data set assembled here to test the intraspecific variation in the multiple life history traits across large-scale environmental effects (e.g latitude, temperature, habitat types) in scombrid species, and at the same time focus on identifying and prioritizing regional life history data-gaps for each individual species. As a second line of research, we encourage future studies to continue determining the population structure of scombrid species using multiple approaches from genetic techniques to the use of biological markers such as otolith microstructure and electronic tagging methods, in order to define geographic boundaries of populations at scales relevant for fisheries management.

## Conclusions

We reviewed and synthesized the life history information on growth and reproductive biology for the 51 species of scombrids, including a population-level review for the principal market tuna species, identified major biological gaps in knowledge and prioritized life history research needs for scombrid species and principal market tuna populations given the life history gaps identified, importance of their fisheries and current conservation status according to the IUCN Red List. We revealed that one third of species (17 spp.) have reasonable information on growth, maturity and fecundity. Half of the species (26 spp.) lack information on either growth, maturity or fecundity, and eight species have no information at all on growth, maturity or fecundity, for which we know little more than their maximum body sizes and their overall distributions. Additionally, we found that the biology of tunas and mackerel species have been more extensively studied than for Spanish mackerels and bonitos, although there are notable exceptions in all the taxonomic groups. Moreover, we also revealed that reproductive biology of species, particular fecundity, is the least-studied biological aspect when compared with growth and maturity.

Given their economic and social importance and the increase in global catches and demand, scombrid species will continue to be central in future fisheries and ecological research. Globally the majority of life history research has focused, and still is focused, on the principal market tuna species and a few temperate mackerel species, giving less priority to the life history research of the rest of coastal scombrid species such as the small tunas, Spanish mackerels and bonitos. In this study, we hope to have raised attention to the urgent need for work on the life history of the smaller coastal scombrid species. Although lower in economic value in the global markets, coastal scombrid species support diverse fisheries throughout their distributions and are an important source of wealth and food security to the local fishing communities in many countries [7], [9], [11], [12]. However, we also emphasize the need to continue field studies, employing proper experimental design and methodologies on the life history data-rich principal market tuna and mackerel species as needed, and especially we advise to focus research to investigate the spatial variation in growth and reproductive traits. Furthermore, we encourage future studies to use the assembled life history data set presented here to develop comparative analyses to make use of the biological knowledge on data-rich scombrid species to data-poor scombrid species with potential similar biology which could potentially have a positive effect in the quality of management advice. Last, by highlighting the important gaps in biological knowledge and providing a priority setting for life history research in scombrid species, we hope this study can serve as a guide for fish biologists and resource managers interested in the biology, ecology and management of scombrid species, particularly in areas of the world where the information is lacking, inadequate or outdated.

## Supporting Information

Appendix S1 **Bibliography of life history data set.**
(DOC)

Figure S1 **Number of studies to estimate age and growth by method type in scombrid species.** Aging methods including direct methods such as calcified structures (vertebrae, spines, scales and otoliths) and indirect methods such as modal analysis of length frequencies and tagging studies, or by various combinations of several of these methods.
(TIF)

Figure S2 **Illustration of the effect of different aging and growth techniques on the estimation of the growth performance index Ø′.** Only species for which there are more than 15 von Bertalanffy growth curves are shown.
(TIF)

Figure S3 **Illustration of the effect of different aging and growth techniques on the estimation of the von Bertalanffy growth parameter** ***k*** **(y^−1^).** Only species for which there are more than 15 von Bertalanffy growth curves are shown.
(TIF)

Figure S4 **Illustration of the effect of different aging and growth techniques on the estimation of longevity** ***T_max_*** **(y).** Only species for which there are more than 15 von Bertalanffy growth curves are shown.
(TIF)

Table S1 **List of scombrid species with a brief description of their fisheries.**
(DOC)

Table S2 **Criteria to construct the Venn Diagram of life history research priorities in scombrid species.** We differentiated between life history data-poor and data-rich species (see definition in main text), between species targeted and not-targeted by commercial fisheries (see Table S1), and between species listed as Threatened, Near Threatened and Data Deficient from those listed as Least Concern in the IUCN Red List [10]. IUCN Red List categories: CR - Critically Endangered, EN - Endangered, VU - Vulnerable, NT - Near Threatened, LC - Least Concern and DD - Data Deficient.
(DOC)
