# Supplementary material for: The Conservation and Management of Tunas and Their Relatives: Setting Life History Research Priorities
Source: PLoS One. 2013 Aug 8;8(8):e70405. doi: 10.1371/journal.pone.0070405 (PMC3738557; doi:10.1371/journal.pone.0070405)
Supplement: Appendix S1 — Bibliography of life history data set. (DOC) [file pone.0070405.s001.doc]

**Appendix 1. Bibliography life history data set.**

1. Abdussamad EM, Koya KPS, Ghosh S, Joshi KK, Manojkumar B, et al. (2012) Fishery, biology and population characteristics of longtail tuna, *Thunnus tonggol* (Bleeker, 1851) caught along the Indian coast. Indian Journal of Fisheries 59: 7-16.

2. Abdussamad EM, Mohamed-Kasim H, Achayya P (2006) Fishery and population characteristics of Indian mackerel, *Rastrelliger kanagurt*a (Cuvier) at Kakinada. Indian Journal of Fisheries 53: 77-83.

3. Abdussamad EM, Pillai NGK, Kasim HM, Mohamed OMMJH, Jeyabalan K (2010) Fishery, biology and population characteristics of the Indian mackerel, *Rastrelliger kanagurta* (Cuvier) exploited along the Tuticorin coast. Indian Journal of Fisheries 57: 17–21.

4. Adam MS, Sibert J, Itano D, Holland K (2003) Dynamics of bigeye (*Thunnus obesus*) and yellowfin (*T. albacares*) tuna in Hawaii's pelagic fisheries: analysis of tagging data with a bulk transfer model incorporating size-specific attrition. Fishery Bulletin 101: 215-228.

5. Agnalt AL (1989) Long-term changes in growth and age at maturity of mackerel, *Scomber scombrus* L., from the North Sea. Journal of Fish Biology 35: 305-311.

6. Aguayo M, Steffens H (1986) Edad y crecimiento de *Scomber japonicus* del norte de Chile. Investigación Pesquera (Chile) 33: 61-76.

7. Aguilar-Salazar FA, Salas-Márquez S, Cabrera-Vázquez MA, Martínez-Aguilar JD (1990) Crecimiento y mortalidad del carito *Scomberomorus cavalla*, en la zona de la costa norte de la Península de Yucatán. Ciencia Pesquera 8: 71-87.

8. Aguirre-Villaseñor H, Morales-Bojórquez E, Morán-Angulo RE, Madrid-Vera J, Valdez-Pineda MC (2006) Indicadores biológicos de la pesquería de sierra (*Scomberomorus sierra*) al sur del Golfo de California, México. Ciencias Marinas 32: 471-484.

9. Aires-da-Silva A, Maunder MN (2007) Status of bigeye tuna in the eastern Pacific Ocean in 2006 and outlook. Inter-American Tropical Tuna Commission, Stock Assessment Report 8: 105-228.

10. Al-Hosni AHS, Siddeek SM (1999) Growth and mortality of the narrowbarred Spanish mackerel, *Scomberomorus commerson* (Lacepède), in Omani waters. Fisheries Management and Ecology 6: 145-160.

11. Al-zibdah M, Odat N (2007) Fishery status, growth, reproduction, biology and feeding habit of two scombrid fish from the Gulf of Aqaba, Red Sea. Lebanese Science Journal 8: 3-20.

12. Alagarswami K, Hiyama Y, Nose Y (1969) Studies on age and growth of the Japanese mackerel. Records of Oceanographic Works in Japan 10: 39-63.

13. Albaret JJ (1977) La reproduction de l'albacore (*Thunnus albacares*) dans le Golfe de Guinée. Cahiers ORSTOM Série océanographie 15: 389-419.

14. Alencar-Vilela MJ, Castello JP (1991) Estudio de la edad y del crecimiento del barrilete (*Katsuwonus pelamis*) en la región sur y sudeste de Brasil. Frente Maritimo 9: 29-35.

15. Alencar-Vilela MJ, Castello JP (1993) Dinámica poblacional del barrilete (*Katsuwonus pelamis*) explotado en la región sudeste-sur del Brasil en el período 1980-1986. Frente Maritimo 14: 111-124.

16. Alves A, de Barros P, Pinho MR (1998) Age and growth of bigeye tuna *Thunnus obesus* captured in the Madeira Archipelago. Collective Volume of Scientific Papers, ICCAT 48: 277-283.

17. Amarasiri C, Joseph L (1986) Skipjack tuna (*K. pelamis*) - Aspects of the biology and fishery from the western and southern coastal waters of Sri Lanka. FAO Indo-Pacific Tuna Development and Management Programme Collective volume of working documents presented at the Expert Consultation on Stock Assessment of Tunas in the Indian Ocean 4-8 December 1986. Colombo, Sri Lanka. pp. 94-107.

18. Anderson RC (1987) Small tunas, seerfishes and billfishes in the Maldives. Report of Workshop on Small Tuna, Seerfish and Billfish in the Indian Ocean December 1987 IPTP/87/GEN/13. Colombo, Sri Lanka. pp. 38-45.

19. Anderson RC, Waheed A (1990) Exploratory fishing for large pelagic species in the Maldives. India.

20. Anderson RC, Waheed Z, Scholz O (1998) Kawakawa (*Euthynnus affinis*). In: Anderson RC, Waheed Z, Adam MS, editors. The tuna fishery resources of the Maldives Maldives Marine Research Bulletin 3. pp. 127-144.

21. Ann HB (1970) On the spawning and maturity of the Pacific mackerel, *Scomber japonicus* Houttuyn. Reports of Fisheries Resources (Korea) 8: 83-95.

22. Anonymous (1974) Valores geográficos de la cpue, distribución de tallas y crecimiento de la albacora (*Thunnus alalunga* B.) durante la temporada 1972 en la región Cántabro-Galaica. Collective Volume of Scientific Papers, ICCAT 2: 150-154.

23. Anonymous (1996) Report of the Final Meeting of the ICCAT Albacore Research Program. Collective Volume of Scientific Papers, ICCAT 43: 1-140.

24. Appukuttan KK, Radhakrishnan-Nair PN, Kunhikoya KK (1977) Studies on the fishery and growth rate of oceanic skipjack *Katsuwonus pelamis* (Linnaeus) at Minicoy Island from 1966 to 1969. Indian Journal of Fisheries 24: 33-47.

25. Arce FM (1987) The Auxis spp. fisheries of Batangas, Philippines. FAO Indo-Pacific Tuna Development and Management Programme IPTP/87/GEN/12. Colombo, Sri Lanka. pp. 137-144.

26. Arcos-Huitrón NE, Torres-Villegas JR (1990) Ciclo reproductor de la macarela del Pacífico *Scomber japonicus* Houttuyn (Pisces: Scombridae) en Bahía Magdalena, Baja California Sur, México. Investigaciones Marinas CICIMAR 5: 37-45.

27. Arena P, Potoschi A, Cefali A (1980) Risultati preliminari di studi sull'eta, l'accrescimento e la prima maturita' sessuale dell'alalunga *Thunnus alalunga* (Bonn.,1788) del Tirreno. Memorie di Biologia Marina e di Oceanografia 10: 71-81.

28. Aripin IE, Showers PAT (2000) Population parameters of small pelagic fishes caught off Tawi-Tawi, Philippines. Fishbyte 23: 21-26.

29. Arocha F, Lee DW, Marcano LA, Marcano JS (2001) Update information on the spawning of yellowfin tuna, *Thunnus albacares*, in the Western Central Atlantic. Collective Volume of Scientific Papers, ICCAT 52: 167-176.

30. Arreguín-Sánchez F, Cabrera MA, Aguilar FA (1995) Population dynamics of the king mackerel (*Scomberomorus cavalla*) of the Campeche Bank, Mexico. Scientia Marina 59: 637-645.

31. Asano K, Tanaka S (1989) Ovarian maturation and spawning of the Japanese common mackerel *Scomber japonicus*. Nippon Suisan Gakkaishi 55: 1715-1726.

32. Ashida H, Tanabe T, Suzuki N (2009) Recent progress on reproductive biology of skipjack tuna in the tropical region of the Western and Central Pacific Ocean. 16 p.

33. Ateş C, Cengiz Deval M, Bök T (2008) Age and growth of Atlantic bonito (*Sarda sarda* Bloch, 1793) in the Sea of Marmara and Black Sea, Turkey. Journal of Applied Ichthyology 24: 546-550.

34. Báez-Hidalgo M, Bécquer U (1994) Fecundidad del bonito *Katsuwonus pelamis* (Linnaeus) y la albacora *Thunnus atlanticus* (Lesson) en Cuba. Revista de Investigaciones Marinas 15: 218-222.

35. Baglin RE, Rivas LR (1977) Population fecundity of western and eastern north Atlantic bluefin tuna (*Thunnus thynnus*). Collective Volume of Scientific Papers, ICCAT 6: 361-365.

36. Baglin REJ (1982) Reproductive biology of western Atlantic bluefin tuna. Fishery Bulletin 80: 121-134.

37. Baird D (1977) Age, growth and aspects of reproduction of the mackerel, *Scomber japonicus* in South African waters (Pisces: Scombridae). Zoologica Africana 12: 347-362.

38. Ballagh AC, Begg GA, Mapleston A, Tobin A (2006) Growth trends of Queensland east coast Spanish mackerel (*Scomberomorus commerson*) from otolith back-calculations. Marine and Freshwater Research 57: 383-393.

39. Bard FX (1973) Etude sur le germon (*Thunnus alalunga* Bonaterre 1788) de l'Atlantique nord. Elements de dynamique de population [These de Doctorat de 3 cycle]. Paris: University Pierre et Marie Curie. 125 p.

40. Bard FX (1981) Le thon germon (*Thunnus alalunga*) de l'Océan Atlantique [PhD Thesis]. Paris: University of Paris. 333 p.

41. Bard FX, Antoine L. Croissance du listao dans l'Atlantique est. In: Symons PEK, Miyake PM, Sakagawa GT, editors; 1986; Madrid. pp. 301-308.

42. Barker J, Kennedy C, Jebreen E (In prep) Fishery dependent monitoring of the Queensland spotted mackerel fishery (Scomberomorus munroi) 2001 to 2003. Brisbane, Queensland.

43. Barrett I (1971) Preliminary observations on the biology and fishery dynamics of the bonito (*Sarda chiliensis*) in Chilean waters. Santiago, Chile. 55 p.

44. Bashirullah AKM (1990) Reproductive biology of Spanish mackerel, *Scomberomorus brasiliensis* Collete, Russo & Zavala-Camin, 1978 (Pisces: Fam: Scombridae) in eastern Venezuela. Boletin del Instituto Oceanografico de Venezuela 29: 91-96.

45. Batista VdS, Fabré NN (2001) Temporal and spatial patterns on serra, *Scomberomorus brasiliensis* (Teleostei Scombridae), catches from the fisheries on the Maranhão coast, Brazil. Brazilian Journal of Biology 61: 541-546.

46. Batts BS (1972) Age and growth of the skipjack tuna, *Katsuwonus pelamis* (Linnaeus), in North Carolina waters. Chesapeake Science 13: 237-244.

47. Batts BS (1972) Sexual maturity, fecundity and sex ratios of the skipjack tuna, *Katsuwonus pelamis* (Linnaeus), in North Carolina waters. Transactions of the American Fisheries Society 101: 626-637.

48. Bayhan B (2007) Growth characteristics of the chub mackerel (*Scomber japonicus* Houttuyn, 1782) in Izmir Bay (Aegean Sea, Turkiye). Journal of Animal and Veterinary Advances 6: 627-634.

49. Bayliff WH (1988) Growth of skipjack, *Katsuwonus pelamis*, and yellowfin, *Thunnus albacares*, tunas in the eastern Pacific Ocean as estimated from tagging data. Bulletin of the Inter-American Tropical Tuna Commission, IATTC 19: 307-385.

50. Bayliff WH, Ishizuka Y, Deriso RB (1991) Growth, movement, and attrition of northern bluefin tuna, *Thunnus thynnus*, in the Pacific Ocean, as determined by tagging Bulletin of the Inter-American Tropical Tuna Commission, IATTC 20: 1-94.

51. Beardsley GL (1971) Contribution to the population dynamics of Atlantic albacore with comments on potential yields. Fishery Bulletin 69: 845-857.

52. Beardsley GL, Richards WJ (1970) Size, seasonal abundance, and length-weight relation of some scombrid fishes from southeast Florida. Washington D. C. 7 p.

53. Beaumariage DS (1973) Age, growth, and reproduction of king mackerel, *Scomberomorus cavalla*, in Florida. Florida Marine Research Publications 1: 1-45.

54. Beerkircher LR (2005) Length to weight conversions for wahoo, *Acanthocybium solandri,* in the northwest Atlantic. Collective Volume of Scientific Papers, ICCAT 58: 1616-1619.

55. Begg GA (1998) Reproductive biology of school mackerel (*Scomberomorus queenslandicus*) and spotted mackerel (*S. munroi*) in Queensland east-coast waters. Marine and Freshwater Research 49: 261-270.

56. Begg GA, Chen CCM, O´Neill MF, Rose DB (2006) Stock assessment of the Torres Strait Spanish mackerel fishery. Townsville, Australia: CRC Reef Research Centre.

57. Begg GA, F ONM, Cadrin SX, Bergenius MAJ (2005) Stock assessment of the Australian east coast spotted mackerel fishery. Townsville, Australia: CRC Reef Research Centre. 1-159 p.

58. Begg GA, Sellin MJ (1998) Age and growth of school mackerel (*Scomberomorus queenslandicus*) and spotted mackerel (*S. munroi*) in Queensland east-coast waters with implications for stock structure. Marine and Freshwater Research 49: 109-120.

59. Bell RR (1962) Age determination of the Pacific albacore of the California coast. California Fish and Game 48: 39-48.

60. Berrien PL (1975) A description of Atlantic mackerel, *Scomber scombrus*, eggs and early larvae. Fishery Bulletin 73: 186-192.

61. Bertignac M, Yesaki M (1993) Preliminary assessment of the narrow-barred Spanish mackerel stock off Oman using length-frequency distributions by the Bhattacharya method. In: Ardill JD, editor. IPTP Collective Volumes No8 Proceedings of the expert consultation on Indian Ocean Tunas, 5th session,4-8 October. Mahe,Seychelles. pp. 88-95.

62. Bigelow HB, Schroeder WC (1953) Tuna*Thunnus thynnus* (Linnaeus) 1758. Fishes of the Gulf of Maine US Fisheries and Wildlife Service, Fishery Bulletin 53. pp. 338-346.

63. Black G (1979) Maturity and spawning of the Pacific bonito *Sarda chiliensis lineolata*, in the eastern north Pacific. California: California Department of Fish and Game.

64. BOBP (1987) Tuna in the Andaman Sea. Colombo, Sri Lanka. 1-64 p.

65. Bök T, Oray IK (2001) Age and growth of bullet tuna *Auxis rochei* (Risso, 1810) in Turkish waters. Collective Volume of Scientific Papers, ICCAT 52: 708-718.

66. Bolaños MA, Tzeng WN (1994) Estimation of growth parameters of two species of mackerel, *Scomber japonicus* and *S. australasicus*, in the coastal waters of Taiwan. Journal of the Fisheries Society of Taiwan 21: 313-321.

67. Boonprakob U. Study on the fecundity of the Indo-Pacific mackerel, *Rastrelliger spp*. in the Gulf of Thailand; 1967; Honolulu, Hawaii, USA. pp. 124-138.

68. Boonragsa V. Tuna resources in the Thai waters of the Andaman Sea; 1987; Colombo, Sri Lanka.

69. Boonraksa V (1988) Growth, mortality and maximum sustainable yield of the Indo-Pacific mackerel (*Rastrelliger brachysoma*) off the southwest coast of Thailand. In: Venema S, Moller-Christensen J, Pauly D, editors. Contributions to tropical fisheries biology FAO/DANIDA Follow-up Training Course on Fish Stock Assessment in the Tropics, Denmark, 1986 and Philippines, 1987 FAO Fisheries Report No 389. Rome. pp. 356-371.

70. Bouhlel M (1985) Stock assessment of the king fish, *Scomberomorus commerson*, inhabiting the coastal waters of Djibouti Republic and State of the fish stocks. Rome. 40 p.

71. Brock VE (1954) Some aspects of the biology of the aku, *Katsuwonus pelamis*, in the Hawaiian Islands. Pacific Science 8: 94-104.

72. Brouard F, Grandperrin R (1985) Les poissons profonds de la pente recifale externe a Vanuatu. Port-Vila, Vanuatu: Mission ORSTOM. 131 p.

73. Brouard F, Grandperrin R, Cillaurren E (1984) Croissance des jeunes thons jaunes (*Thunnus albacares)* et des bonites (*Katsuwonus pelamis*) dans le Pacifique tropical occidental. Port-Vila, Vanuatu: Mission ORSTOM. 1-23 p.

74. Brown-Peterson NJ, Franks JS, Burke AM. Preliminary observations on the reproductive biology of wahoo, Acanthocybium solandri, from the northern Gulf of Mexico and Bimini, Bahamas; 2000; Ocean Springs, USA. pp. 414-427.

75. Buckworth RC (1998) Age structure of the commercial catch of northern territory narrow- barred Spanish mackerel. Darwin, Australia: Northern Territory Department of Primary Industry and Fisheries. 28 p.

76. Buñag DM (1956) Spawning habits of some Philippine tuna based on diameter measurements of the ovarian ova. Journal of Philippine Fisheries 4: 145-176.

77. Cabrera MA, Defeo O, Aguilar F, Martínez JDD (2005) La pesquería de bonito (*Euthynnus alletteratus*) del noreste del banco de Campeche, México. Proceedings of the Gulf and Caribbean Fisheries Institute 46: 744-758.

78. Cabrera-Vazquez MA (1986) Contribución al conocimiento de la pesquería del carito (*Scomberomorus cavalla*) Cuvier 1829, en la Península de Yucatan [Tesis Profesional]. ENEP- Iztacala, México: Universidad Nacional Autónoma de México.

79. Caddy JF, Dickson CA, Butler MJA (1976) Age and growth of giant bluefin tuna (*Thunnus thynnus thynnus*) taken in Canadian waters in 1975. Fisheries Research Board of Canada Report Series 1395: 1-18.

80. Calkins TP, Klawe WL. Synopsis of biological data on black skipkjack, *Euthynnus lineatus*, Kishinouye, 1920; 1963; Rome. FAO. pp. 130-146.

81. Cameron D, Begg G (2002) Fisheries biology and interaction in the northern Australian small mackerel fishery.

82. Campbell G, Collins RA (1975) The age and growth of the pacific bonito, *Sarda chiliensis*, in the eastern north Pacific. California Fish and Game 61: 181-200.

83. Cantanhêde da Silva G, Leal de Castro AC, Gubiani EA (2005) Estrutura populacional e indicadores reprodutivos de *Scomberomorus brasiliensis* Collette, Russo e Zavala-Camin, 1978 (Perciformes: Scombridae) no litoral ocidental maranhense. Acta Scientiarum Biological Sciences 27: 383-389.

84. Caramantin-Soriano H, Vega-Pérez LA, Ñiquen M (2008) Growth parameters and mortality rate of the *Scomber japonicus peruanus* (Jordán & Hubb,1925) along the peruvian coast, south Pacific. Brazilian Journal of Oceanography 56: 201-210.

85. Carles-Martín CA (1971) Características biológico-pesqueras del bonito (*Katsuwonus pelamis*) y la albacora (*Thunnus atlanticus*) en la costa noreste de Cuba. Revista de Investigación Pesquera, Instituto Nacional de Pesca, Cuba 32: 1-51.

86. Carles-Martín CA (1975) Evaluación de la pesquería de bonito en la zona Occidental de Cuba. Revista de Investigación Pesquera, Instituto Nacional de Pesca, Cuba 1: 73-96.

87. Carles-Martín CA (1975) Edad y crecimiento del bonito (*Katsuwonus pelamis*) y la albacore (*Thunnus atlanticus*) en la parte occidental de Cuba. Revista de Investigación Pesquera, Instituto Nacional de Pesca, Cuba 1: 203-254.

88. Carneiro Ximenes MO (1981) Idade e crescimiento da serra, *Scomberomorus brasiliensis,* no estado do Cearã (Brasil). Arquivos de Ciências do Mar 21: 47-54.

89. Carneiro Ximenes MO, Ferreira de Menezes M, Fonteles-Filho AA (1978) Idade e crescimento da cavala, *Scomberomorus cavalla* (Cuvier), no estado do Cearã (Brasil). Arquivos de Ciências do Mar 18: 73-81.

90. Carvalho N, Perrota RG, Isidro EJ (2002) Age, growth and maturity in chub mackerel (*Scomber japonicus* Houttuyn, 1782) from the Azores. Arquipélago Ciências Biológicas e Marinhas 19: 93-99.

91. Castello JP, Cousseau MB (1976) Observaciones sobre la caballa en las temporadas de pesca del período 1969 a 1975 (Pisces, *Scomber japonicus marplatensis*). Physis 35: 195-2003.

92. Castello JP, Gagliardi RP (1969) Informe sobre estudios de edad y maduración sexual en el bonito (*Sarda sarda*, Bloch, 1793). Oficina Regional de Pesca para America Latina, Rio de Janeiro. 16 p.

93. Castello JP, Hamre J (1969) Age and growth of mackerel from Skagerak and the northern North Sea. International Council for the Exploration of the Sea CM H:7: 1-12.

94. Cayré P, Amon Kothias JB, Diouf T, Stretta J (1993) Biology of tuna. In: Fonteneau A, Marcille J, editors. Resources, fishing and biology of the tropical tunas of the Eastern Central Atlantic FAO Fisheries Technical Paper No 292. Roma: FAO. pp. 354.

95. Cayré P, Amon Kothias JB, Diouf T, Stretta JM (1988) Biologie des thons. In: Fonteneau A, Marcille J, editors. Resources, fishing and biology of the tropical tunas of the eastern central Atlantic FAO Fisheries Technical Paper, 292. Roma. pp. 157-268.

96. Cayré P, Diouf T (1980) Croissance de la thonine (*Euthynnus alletteratus*) (Rafinesque, 1810) etablie a partir de coupes tranversales du premier rayon de la nageoire dorsale. Document Scientifique - Centre de Recherches Océanographiques de Dakar - Thiaroye 75: 18.

97. Cayré P, Diouf T (1983) Estimating age and growth of little tunny, *Euthynnus alletteratus*, off the coast of Senegal, using dorsal fin spine sections. US Department of Commerce, NOAA Technical Report, NMFS 8: 105-110.

98. Cayré P, Diouf T (1984) Croissance du thon obese (*Thunnus obesus*) de l'Atlantique d'apres les resultats de marquage. Collective Volume of Scientific Papers, ICCAT 20: 180-187.

99. Cayré P, Farrugio H. Biologie de la reproduction du Listao (*Katsuwonus pelamis*) de l'Océan Atlantique. In: Symons PEK, Miyake PM, Sakagawa GT, editors; 1986; Madrid. pp. 252-272.

100. Cayré P, Laloé F. Relation poids-longueur du listao (*Katsuwonus pelamis*) de l'Océan Atlantique. In: Symons PEK, Miyake PM, Sakagawa G, editors; 1986; Paris, France. pp. 335-340.

101. Chale-Matsau JR, Govender A, Beckley LE (1999) Age and growth of the queen mackerel *Scomberomorus plurilineatus* from KwaZulu-Natal, South Africa. Fisheries Research 44: 121-127.

102. Champagnat C, Pianet R (1974) Croissance du patudo (*Thunnus obesus*) dans les régions de Dakar et de Pointe-Noire. Collective Volume of Scientific Papers, ICCAT 2: 141-144.

103. Chang KH, Wang TS (1970) Studies on fecundity and spawning of spotted mackerel in Taiwan. China Fisheries Monthly 211: 8-14.

104. Chang SK, Liu HC, Hsu CC (1993) Estimation of vital parameters for Indian albacore through length frequency data. Journal of the Fisheries Society of Taiwan 20: 1-13.

105. Chávez EA (1994) Simulación de la pesquería de sierra (*Scomberomorus maculatus*) del Golfo de México. Revista de Investigaciones Marinas 15: 209-218.

106. Chen JH, Lin LS (2004) Study on the biological characteristics and status of common mackerel (*Scomber japonicus* Houttuyn) fishery in the east China Sea region. Marine Fisheries 26: 73-78.

107. Chen K-S, Shimose T, Tanabe T, Chen C-Y, Hsu C-C (2012) Age and growth of albacore *Thunnus alalunga* in the North Pacific Ocean. Journal of Fish Biology 80: 2328–2344.

108. Chen KS, Crone P, Hsu CC (2006) Reproductive biology of female Pacific bluefin tuna *Thunnus orientalis* from south-western north Pacific Ocean. Fisheries Science 72: 985-994.

109. Chen KS, Crone PR, Hsu CC (2010) Reproductive biology of albacore *Thunnus alalunga*. Journal of Fish Biology 77: 119-136.

110. Chen TS (1973) Studies on the age, growth, maturity and spawning of Spanish mackerel *Scomberomorus commersoni* (Lacépéde) in Taiwan Strait. Bulletin of TFRI 22: 103-118.

111. Chen TS (1974) Studies on the age, growth, maturity, and spawning of Japanese mackerel *Scomberomorus niphonius* (C. & V.) in Taiwan Strait. Bulletin of Taiwan Fisheries Research Institute 23: 21-36.

112. Cheunpan A (1984) Sexual maturity, size at first maturity and spawning season of long tail tuna (*Thunnus tonggol*), eastern litte tuna (*E. affinis*) and frigate mackerel (*A. thazard*) in the Gulf of Thailand. Bangkok. 33 p.

113. Cheunpan A (1988) An assessment of king mackerel (*Scomberomorus commerson*) in the inner Gulf of Thailand. In: Venema S, Moller-Christensen J, Pauly D, editors. Contributions to tropical fisheries biology FAO/DANIDA Follow-up Training Courses on Fish Stock Assessment in the Tropics, Denmark, 1986 and Philippines, 1987 FAO Fisheries Report No 389. Rome: FAO.

114. Chi KS, Yang RT (1973) Age and growth of skipjack tuna in the waters around the southern part of Taiwan. Acta Oceanographica Taiwanica 3: 199-122.

115. Chiou WD, Cheng LZ, Chen KW (2004) Reproduction and food habits of kawakawa *Euthynnus affinis* in Taiwan. Journal of the Fisheries Society of Taiwan 31.

116. Chirinos De Vildoso A (1963) Estudios sobre la reproduccion del bonito *Sarda chilensis* (C. y V.) en aguas adyacentes a la costa Peruana. Rome. 1143-1152 p.

117. Chisara PK (1986) A preliminary report on the biology and fishery of *Scomberomorus lineolatus* in the Zanzibar Channel. FAO Indo-Pacific Tuna Development and Management Programme Collective volume of working documents presented at the Expert Consultation on Stock Assessment of Tunas in the Indian Ocean, 4-8 December 1986 Colombo, Sri Lanka. pp. 124-134.

118. Chong BJ, Chua CW (1974) Growth, age determination and spawning of ikan kembung, *Rastrelliger neglectus* (Van Kampen) in the northern Straits of Malacca. The Malaysian Agricultural Journal 49: 344-345.

119. Chur VN, Zharov VL (1983) Determination of age and growth rate of the skipjack tuna, *Katsuwonus pelamis* (Scombridae), from the southeastern part of the Gulf of Guinea. Journal of Ichthyology 23: 53-67.

120. Ciechomski JD, Capezzani DA (1969) Fecundity of the Argentinean mackerel *Scomber japonicus marplatensis*. Marine Biology 2: 277-282.

121. Cisneros MA, Estrada J, Montemayor G (1990) Growth, mortality and recruitment of exploited small pelagic fishes in the Gulf of California, Mexico. Fishbyte 8: 15-17.

122. Claereboudt MR, McIlwain JL, Al-Oufi HS, Ambu-Ali AA (2005) Patterns of reproduction and spawning of the kingfish (*Scomberomorus commerson*, Lacépède) in the coastal waters of the Sultanate of Oman. Fisheries Research 73: 273-282.

123. Claereboudt MRG, Al-oufi HS, McIlwain JL, Goddard JS (2004) Relationships between fishing gear, size frequency and reproductive patterns for the kingfish (*Scomberomorus commerson* Lacépède) fishery in the Gulf of Oman. In: Payne AIL, O’Brien CM, Rogers SI, editors. Management of Shared Fish Stocks: Oxford, Blackwell. pp. 56-67.

124. Claro R (1994) Características generales de la ictiofauna. Qintana Roo, México: Instituto de Oceanología Academia de Ciencias de Cuba and Centro de Investigaciones de Quintana Roo.

125. Clemens HB (1961) The migration, age, and growth of Pacific albacore (*Thunnus germo*), 1951-1958. Fish Bulletin California Department of Fish and Game 115: 1-128.

126. Collette BB (1986) Family Scombridae. In: Smith MM, Heemstra PC, editors. Smiths' Sea Fishes. MacMillan, Johannesburg. pp. 831-838.

127. Collette BB (2001) Tunas (also, albacore, bonitos, mackerels, seerfishes, and wahoo). In: Carpenter KE, Niem VH, editors. FAO Species Identification Guide for Fishery Purposes The Living Marine Resources of the Western Central Pacific. Rome: FAO. pp. 3721-3735.

128. Collette BB, Gillis GB (1992) Morphology, systematics, and biology of the double-lined mackerels (*Grammatorcynus*, Scombridae). Fishery Bulletin 90: 13-53.

129. Collette BB, Nauen CE (1983) FAO Species Catalogue. Vol. 2. Scombrids of the world: an annotated and illustrated catalogue of tunas, mackerels, bonitos and related species known to date. FAO Fisheries Synopsis 125: 137.

130. Collins MR, Schmidt DJ, Wayne-Waltz C, Pickney JL (1988) Age and growth of king mackerel, *Scomberomorus cavalla*, from the Atlantic coast of the United States. Fishery Bulletin 87: 49-61.

131. Compeán-Jimenez G, Bard FX (1983) Growth increments on dorsal spines of eastern Atlantic bluefin tuna (*Thunnus thynnus*) and their possible relation to migrations patterns. NOAA Technical Report NMFS 8: 77-86.

132. Cooksey CL (1996) Reproductive biology of Spanish mackerel, *Scomberomorus maculatus*, in the lower Chesapeake Bay [Master Thesis]. Virginia: Virginia Institute of Marine Science. 68 p.

133. Coombs SH, Pipe RK, Mithchell CE (1981) The vertical distribution of eggs and larvae of blue whiting (*Micromesistius poutassou*) and mackerel (*Scomber scombrus*) in the eastern north Atlantic and North Sea. Rapports et Proces-Verbaux des Reunions du Conseil International Pour L'Explotation de la Mer 178: 188-195.

134. Corpuz A, Saeger J, Sambilay V (1985) Population parameters of commercially important fishes in Philippine waters. 100 p.

135. Correa-Ivo CT (1974) Sobre a fecundidade da cavala, *Scomberomorus cavalla* (Cuvier), em águas costeiras do estado do Ceará (Brasil). Arquivos de Ciências do Mar 14: 87-89.

136. Corriero A, Karakulak S, Santamaria N, Deflorio M, Spedicato D, et al. (2005) Size and age at sexual maturity of female bluefin tuna (*Thunnus thynnus* L. 1758) from the Mediterranean Sea. Journal of Applied Ichthyology 21: 483-486.

137. Cort JL (1991) Age and growth of bluefin tuna (*Thunnus thynnus thynnus*) of the northeast Atlantic. Collective Volume of Scientific Papers, ICCAT 35: 213-230.

138. Cort JL, González S, Ilardia S (1986) La pesquería de caballa (o Verdel) (*Scomber scombrus*, Linnaeus, 1758) en el Mar Cantábrico, 1982-1983. Informe Técnico Instituto Español de Oceanografía 38: 1-12.

139. Cousseau MB, Angelescu V, Perrotta RG (1987) Algunas características de la estructura y comportamiento migratorio de los cardúmenes de caballa (*Scomber japonicus marplatensis*) en la plataforma bonaerense (Mar Argentino). Período 1965 - 1984. Revista de Investigación y Desarrollo Pesquero 7: 21-42.

140. CRFM (2007) CRFM Fishery Report - 2007. Volume 1. St. Vincent and the Grenadines.

141. Cucalón-Zenck E (1999) Growth and length-weight parameters of Pacific mackerel (*Scomber japonicus*) in the Gulf of Guayaquil, Ecuador. Naga, The ICLARM Quaterly 22: 32-36.

142. D'Aubenton F, Blanc M (1965) Étude systématique et biologique de *Scomberomorus sinensis* (Lacépède, 1802), poisson des eaux douces du Cambodge. Bulletin du Muséum National d'Histoire Naturelle 37: 233-243.

143. da Cruz JF, Paiva MP (1964) Sobre a biologia pesqueira da albacora, *Thunnus atlanticus* (Lesson), no nordeste do Brasil. Boletim do Instituto de Biologia Marinha da Universidade Federal do Rio Grande do Norte 1: 1-15.

144. Darvishi M, Behzadi S, Salarpour A (2003) Spawing, fecundity and feeding of longtail tuna (*Thunnus tonggol*) in the Persian Gulf and Oman Sea (Hormuzgan Province). Ministry of Jahad Sazandegi Pajouhesh and Sazandegi 59: 70-75.

145. Davidoff EB (1963) Size and year class composition of catch, age, and growth of yellowfin tuna in the eastern tropical Pacific Ocean, 1951-1961. Bulletin of the Inter-American Tropical Tuna Commission, IATTC 8: 199-251.

146. Davis T, Farley J, Gunn J (2001) Size and age at 50% maturity in SBT. An integrated view from published information and new data from the spawning ground. Tokyo, Japan. 10 p.

147. Dawson WA (1986) The interpretation of otolith structure for the assessment of age and growth of some pelagic fishes from the coast of Ecuador. Mackerel (*Scomber japonicus* L.), Pacific sardine (*Sardinops sagax*), Pacific thread herrings (*Opisthonema medirastre* and *Opisthone bulleri*), and round herring (*Etrumeus teres*). Instituto Nacional de Pesca, Boletin Scientifico y Tecnico 9: 1-24.

148. Dayaratne P (1989) Age, growth and mortality estimates of *Scomberomorus commerson* (Seerfish) from the west coast of Sri Lanka.

149. Dayaratne P (1993) An assessment of frigate tuna (*Auxis thazard*) stocks in the southern waters of Sri Lanka. IPTP Collective Volumes No 8: 72-76.

150. Dayaratne P, De Silva J (1991) An assessment of kawakawa (*Euthynnus affinis*) stock on the west coast of Sri Lanka. Asian Fisheries Science 4: 219-226.

151. Dayaratne P, Sivakumaran KP (1994) Biosocioeconomics of fishing for small pelagics along the southwest coast of Sri Lanka. Madras, India. 38 p.

152. de la Hoz Regules J, Villegas Cuadros ML (1987) Biometrie, croissance, reproduction et pêche de *Scomber scombrus,* L. 1768, aux cotes Asturiennes (Nord de L'Espagne) en 1985. International Council for the Exploration of the Sea CM H:5: 1-12.

153. de La Serna JM, Ortiz de Urbina JM, Alot E, Garcia S, Rioja P (2005) Biological parameters of bullet tuna (*Auxis rochei*) observed in the Spanish Mediterranean fisheries. Collective Volume of Scientific Papers, ICCAT 58: 517-526.

154. de León ME, Guardiola M (1984) Caracterización biológico-pesquera del género Scomberomorus de la zona suroriental de Cuba. Cuban Journal of Fisheries Research 9: 1-27.

155. De Metrio G, Cacucci M, Corriero A, Santamaria N, Spedicato D (1998) Indagini sulla pesca e la biologia dei grandi pelagici (*Thunnus thynnus* L., *Thunnus alalunga* Bonn., *Sarda sarda* Bloch, *Xiphias gladius* L., *Auxis rochei* Risso) nello Ionio settentrionale dal 1990 al 1997. Biologia Marina Mediterranea 5: 215-228.

156. De Metrio G, Megalofonou P, Marano G, De Zio V, Rosinati L, et al. (1994) Observations of a ten-year period on the biology and fishery of albacore, *Thunnus alalunga* (Bonn. 1788), carried out in the north Ionian and south Adriatic seas. FAO Fisheries Report No 533: 115-125.

157. de Nóbrega MF, Lessa RP (2009) Age and growth of Spanish mackerel (*Scomberomorus brasiliensis*) off the northeastern coast of Brazil. Neotropical Ichthyology 7: 667-676.

158. de Nóbrega MF, Lessa RPT, de Lucena FM (2001) Idade e crescimento da Serra (*Scomberomorus brasiliensis*) capturada na ZEE do nordeste do Brasil. Foz do Iguaçu.

159. De Sylva DP, Rathjen WF (1961) Life history notes on the little tuna, *Euthynnus alletteratus*, from the southeastern United States. Bulletin of Marine Science of the Gulf and Caribbean 11: 161-190.

160. Delgado de Molina A, Santana JC (1986) Estimación de la edad y crecimiento del patudo (*Thunnus obesus*, Lowe, 1939) capturado en las Islas Canarias. Collective Volume of Scientific Papers, ICCAT 25: 130-137.

161. Delgado de Molina A, Santana JC, Ariz J, Delgado de Molina R, Pallares P (1994) Estudio de algunos parámetros biológicos del rabil (*Thunnus albacares*, Bonnaterre 1788) del Atlántico este. Collective Volume of Scientific Papers, ICCAT 42: 153-154.

162. Devaraj M (1981) Age and growth of three species of seerfishes *Scomberomorus commerson*, *S. guttatus* and *S. lineolatus*. Indian Journal of Fisheries 28: 104-127.

163. Devaraj M (1983) Maturity, spawning and fecundity of the king seer, *Scomberomorus commerson*, in the seas around the Indian Peninsular. Indian Journal of Fisheries 30: 203-230.

164. Devaraj M (1986) Maturity, spawning and fecundity of the streaked seer, *Scomberomorus lineolatus* (Cuvier & Valenciennes) in the Gulf of Mannar and Palk Bay. Indian Journal of Fisheries 33: 293-319.

165. Devaraj M (1987) Maturity, spawning and fecundity of the spotted seer, *Scomberomorus guttatus*, in the Gulf of Mannar and Palk Bay. Indian Journal of Fisheries 34: 48-77.

166. Devaraj M, Mohamad-Kasim H, Muthiah C, Pillai NGK (1999) Assessment of the exploited seerfish stocks in the Indian waters. Journal of the Marine Biological Association of India 41: 62-84.

167. DeVries DA, Grimes CB (1997) Spatial and temporal variation in age and growth of king mackerel, *Scomberomorus cavalla*, 1977-1992. Fishery Bulletin 95: 694-708.

168. Di Natale A, Mangano A, Celona A, Navarra E, Valastro M (2005) First information about the Atlantic bonito (*Sarda sarda)* catch composition in the Tyrrhenian Sea and in the Straits of Sicily in 2002 and 2003. Collective Volume of Scientific Papers, ICCAT 58: 1537-1542.

169. Di Natale A, Mangano A, Celona A, Navarra E, Valastro M (2006) Atlantic bonito (*Sarda sarda*) catch composition in the Tyrrhenian Sea and in the Strait of Sicily in 2004. Collective Volume of Scientific Papers, ICCAT 59: 564-570.

170. Diaz GA (2011) A revision of western Atlantic bluefin tuna age of maturity derived from size samples collected by the Japanese longline fleet in the Gulf of Mexico (1975-1980). Collective Volume of Scientific Papers, ICCAT 66: 1216–1226.

171. Diaz GA, Turner SC (2006) Size frequency distribution analysis, age composition, and maturity of western bluefin tuna in the Gulf of Mexico from the U.S. (1981-2005) and Japanese (1975-1981) longline fleets. Collective Volume of Scientific Papers, ICCAT: 1-11.

172. Dickerson TL, Macewicz BJ, Hunter JR (1992) Spawning frequency and batch fecundity of chub mackerel, *Scomber japonicus*, during 1985. The California Cooperative Oceanic Fisheries Investigations Reports 33: 130-140.

173. Diouf T (1980) Peche & biologie de trois Scombridae exploités au Sénégal: *Euthynnus alletteratus*, *Sarda sarda* et *Scomberomorus tritor* [These de Doctorat de 3 cycle]. Brest: Université de Bretagne Occidentale. 159 p.

174. Diouf T (1988) Relation taille-poids de *Auxis thazard* peche en Atlantique Tropical Oriental. Collective Volume of Scientific Papers, ICCAT 28: 314-317.

175. Djabali F, Boudraa S, Bouhdid A, Bousbia H, Bouchelaghem EH, et al. (1990) Travaux réalisés sur les stocks pélagiques et démersaux de la région de Béni-saf. FAO Fisheries Report No 447: 160-165.

176. Doray M, Stéquert B, Taquet M (2004) Age and growth of blackfin tuna (*Thunnus atlanticus*) caught under moored fish aggregating devices, around Martinique Island. Aquatic Living Resources 17: 13-18.

177. Dorel D (1986) Poissons de l’Atlantique nord-est: Relations taille-poids. Nantes, France: IFREMER. 165 p.

178. Dorval E, Hill KT, Lo NCH, McDaniel JD (2007) Pacific mackerel (*Scomber japonicus*) stock assessment for U.S. management in the 2007-08 fishing season. Pacific Fishery Management Council, June 2007 Briefing Book, Agenda Item F2b, Attachment 1. La Jolla, USA. pp. 253.

179. Draganik B, Pelczarski W (1984) Growth and age of bigeye and yellowfin tuna in the central Atlantic as per data gathered by R/V "WIECZNO". Collective Volume of Scientific Papers, ICCAT 20: 96-103.

180. Duarte-Neto P, Higa FM, Lessa RP (2012) Age and growth estimation of bigeye tuna, Thunnus obesus (Teleostei: Scombridae) in the southwestern Atlantic. Neotropical Ichthyology 10: 148–158.

181. Dudley RG, Aghanashinikar AP (1987) Preliminary Studies of *Scomberomorus commerson* and *Thunnus Tonggol* in Omani Waters. Colombo, Sri Lanka.

182. Dudley RG, Aghanashinikar AP (1989) Growth of *Scomberomorus commerson* in Oman based on length data. Report of the workshop on tuna and seer fishes in the north Arabian Sea region, Muscat, Sultanate of Oman, 7-9 February 1989 IPTP/89/Gen/16. Colombo, Sri Lanka. pp. 72-81.

183. Dwiponggo A, Hariati T, Banon S, Palomares ML, Pauly D (1986) Growth, mortality and recruitment of commercially important fishes and penaeid shrimps in Indonesian waters. ICLARM Technical Report 17: 1-91.

184. Edwards RRC, Bakhader A, Shaher S (1985) Growth, mortality, age composition and fisheries yields of fish from the Gulf of Aden. Journal of Fish Biology 27: 13-21.

185. Edwards RRC, Shaher S (1991) The biometrics of marine fishes from the Gulf of Aden. Fishbyte 2: 27-29.

186. Eltink A, Gerritsen J (1982) Growth, spawning and migration of western mackerel. International Council for the Exploration of the Sea CM H:31.

187. Espino Barr E, Cruz Romero M, Garcia Boa A (1990) Biología pesquera de tres especies de la familia Scombridae en el litoral de Colima, México. In: Dailey M, Bertsch H, editors. Memorias del VIII Simposium Internacional de Biologia Marina. Ensenada, México. pp. 65-74.

188. Fable WA, Johnson AG, Barger LE (1987) Age and growth of Spanish mackerel, *Scomberomorus maculatus*, from Florida and the Gulf of Mexico. Fishery Bulletin 85: 777-783.

189. Farley JH, Clear NP, Leroy B, Davis TLO, McPherson G (2006) Age, growth and preliminary estimates of maturity of bigeye tuna, *Thunnus obesus*, in the Australian region. Marine and Freshwater Research 57: 713-724.

190. Farley JH, Davis TLO (1998) Reproductive dynamics of southern bluefin tuna, *Thunnus maccoyii*. Fishery Bulletin 96: 223-236.

191. Farley JH, Davis TLO, Gunn JS, Clear NP, Preece AL (2007) Demographic patterns of southern bluefin tuna, *Thunnus maccoyii*, as inferred from direct age data. Fisheries Research 83: 151-161.

192. Farley JH, Williams AJ, Davies CR, Clear NP, Eveson JP, et al. (2012) Population Biology of Albacore Tuna in the Australian Region. Castray Esplanade, Hobart, Tas, Australia.

193. Farrugio H (1980) Age et croissance du thon rouge (*Thunnus thynnus*) dans la pêcherie française de surface en Méditerranée. Cybium (3ème sér) 9: 45-59.

194. Fernández M (1992) Revision des methodes d'ageage du germon (*Thunnus alalunga*, Bonn. 1788) nord-est Atlantique par l'etude des pieces anatomiques calcifiees. Collective Volume of Scientific Papers, ICCAT 39: 225-240.

195. Figuerola-Fernández M, Peña-Alvarado N, Torres-Ruiz W (2008) Aspect of the reproductive biology of recreationally important fish species in Puerto Rico. Puerto Rico. 1-134 p.

196. Figuerola-Fernández M, Torres-Ruiz W, Peña-Alvarado N (2007) Sexual maturity and reproductive seasonality of king mackerel (*Scomberomorus cavalla*) and cero (*Scomberomorus regalis*) in Puerto Rico. Proceedings of the Gulf and Caribbean Fisheries Institute 58: 251-261.

197. Finucane JH, Collins LA (1984) Reproductive biology of cero, *Scomberomorus regalis*, from the coastal waters of south Florida. Northeast Gulf Science 7: 101-107.

198. Finucane JH, Collins LA (1986) Reproduction of Spanish mackerel, *Scomberomorus maculatus,* from the southeastern United States. Northeast Gulf Science 8: 97-106.

199. Fitzhugh G, Fioramonti C, Walling W, Gamby M, Lyon H, et al. Batch fecundity and an attempt to estimate spawning frequency of king mackerel (*Scomberomorus cavalla*) in U.S. waters; 2008; Gosier, Guadeloupe, French West Indies.

200. Foreman TJ, Ishizuka Y (1990) Giant bluefin tuna off southern California, with a new California size record. California Fish and Game 76: 181-186.

201. Forsbergh ED (1989) The influence of some environmental variables on the apparent abundance of skipjack tuna, *Katsuwonus pelamis*, in the eastern Pacific Ocean. Bulletin of the Inter-American Tropical Tuna Commission, IATTC 19: 429-569.

202. Frade F, Postel E (1955) Contribution à l’etude de la reproduction des scombridés et thonidés de l’Atlantique tropical. Rapport du Conseil International pour l’Exploration de la Mer 137: 33-35.

203. Franco L (1992) Maduración sexual y fecundidad del carite (*Scomberomorus maculatus*) de las costas del estado Falcón, Venezuela. Zootecnia Tropical 10: 157-169.

204. Franičević M, Sinovčić G, Čikes-Keč V, Zorica B (2005) Biometry analysis of the Atlantic bonito, *Sarda sarda* (Bloch, 1793), in the Adriatic Sea. Acta Adriatica 46: 213 - 222.

205. Franks JS, Brown-Peterson NJ, Griggs MS, Garber NM, Warren JR, et al. (2000) Potential of the first dorsal fin spine for estimating the age of wahoo, *Acanthocybium solandri,* from the northern Gulf of Mexico, with comments on specimens from Bimini, Bahamas. 51 Proceedings of the Fifty First Annual Gulf and Caribbean Fisheries Institute. St. Croix US Virgin Islands. pp. 428-440.

206. Freire KMF, Lessa R, Lins-Oliveira JE (2005) Fishery and biology of blackfin tuna (*Thunnus atlanticus*) off northeastern Brazil. Gulf and Caribbean Research 17: 15-24.

207. Frota LO, Costa PAS, Braga AC (2004) Length-weight relationships of marine fishes from the central Brazilian coast. Naga, WorldFish Center Quaterly 27: 20-26.

208. Fu SC (2004) Reproductive biology of skipjack tuna, *Katsuwonus pelamis*, in the western and central Pacific Ocean and waters off eastern Taiwan [Master Thesis]. Taipei, Taiwan (in Chinese): National Taiwan University.

209. Funicane JH, Collins LA, Brusher HA, Saloman CH (1986) Reproductive biology of king mackerel, *Scomberomorus cavalla*, from the southeastern United States. Fishery Bulletin 84: 841-850.

210. Gaikov VV, Chur VN, Zharov VL, Fedoseev YF (1980) On age and growth ot the Atlantic bigeye tuna. Collective Volume of Scientific Papers, ICCAT 9: 294-302.

211. Ganga U (2010) Investigations on the biology of Indian Mackerel Rastrelliger kanagurta (Cuvier) along the Central Kerala coast with special reference to maturation, feeding and lipid dynamics [PhD Thesis]. Kochi, India: Cochin University of Science and Technology. 175 p.

212. García-Coll I (1987) Relaciones largo-peso y proporción de sexos del bonito (*Katsuwonus pelamis*) y la albacora (*Thunnus atlanticus*) de Cuba. Revista de Investigaciones Marinas 8: 83-97.

213. García-Coll I (1988) Edad y crecimiento de la albacora, (*Thunnus atlanticus*) en la región sur-occidental de Cuba durante el período 1979 a 1983. Revista de Investigaciones Marinas 9: 53-59.

214. García-Coll I, Álvarez-Lajonchere LS, Noyola-Ugalde JI (1984) Determinación de la edad y el crecimiento del bonito, *Katsuwonus pelamis* (Linné) y la albacora, *Thunnus atlanticus* (Lesson) en la región suroccidental de Cuba en el año 1979. Revista de Investigaciones Marinas 5: 95-127.

215. García-Coll I, Bosch-Méndez A (1986) Determinación de la edad y el crecimiento del bonito (*Katsuwonus pelamis*) y la albacora (*Thunnus atlanticus*) en la región nororiental de Cuba. Revista de Investigaciones Marinas 7: 47-54.

216. García-Franco W, Cota-Villavicencio A, Sánchez-Ruiz FJ (2001) Diagnóstico de la pesquería de peces pelágicos menores en la costa occidental de Baja California, México. Ciencia Pesquera 14: 113-120.

217. George KC, Banerji SK (1964) Age and growth studies on the Indian mackerel *Rastrelliger kanagurta* (Cuvier) with special reference to length-frequency data collected at Cochin. Indian Journal of Fisheries 11: 621-638.

218. George S, Singh-Renton S, Lauckner B. Assessment of wahoo (*Acanthocybium solandri*) fishery using eastern Caribbean data. In: Singh-Renton S, editor; 2000; Belize City, Belize. pp. 24-50.

219. Ghodrati Shojaei M, Taghavi-Motlagh SA, Seyfabadi J, Abtahi B, Dehghani R (2007) Age, growth and mortality rate of the narrow-barred Spanish mackerel (*Scomberomerus commerson* Lacepède, 1800) in coastal waters of Iran from length frequency data. Turkish Journal of Fisheries and Aquatic Sciences 7: 115-121.

220. Ghosh S, Pillai NGK, Dhokia HK (2009) Fishery, population dynamics and stock assessment of the spotted seer in gill net fishery at Verabal. Indian Journal of Fisheries 56: 157-161.

221. Ghosh S, Sivadas M, Abdussamad EM, Rohit P, Koya KPS, et al. (2012) Fishery, population dynamics and stock structure of frigate tuna Auxis thazard (Lacepede , 1800) exploited from Indian waters. Indian Journal of Fisheries 59: 95-100.

222. Giacchetta F, Santamaria N, De Metrio P, De Metrio G (1995) Biologia e pesca della palamita (*Sarda sarda*, Bloch) nel Golfo di Taranto. Biologia Marina Mediterranea 2: 485-486.

223. Gluyas-Millán MG (1989) Período de reproducción, distribución de tallas y relación longitud-peso de la macarela del litoral de Baja California. Investigaciones Marinas CICIMAR 4: 65-72.

224. Gluyas-Millán MG, Quiñonez-Velázques C (1997) Age, growth, and reproduction of Pacific mackerel *Scomber japonicus* in the Gulf of California. Bulletin of Marine Science 61: 837-847.

225. Gnanamuthu JC, Girijavallabhan KG (1984) Some preliminary observations on the *Rastrelliger faughni* matsui occuring in the Madras coastal waters. Indian Journal of Fisheries 31: 383-386.

226. Godsil HC (1955) A description of two species of bonito *Sarda orientalis* and *S. chiliensis* and a consideration of relationships within the genus. 43 p.

227. Goldberg SR, Au DWK. The spawning of skipjack tuna from southeastern Brazil as determined from histological examination of ovaries. In: Symons PEK, Miyake PM, Sakagawa GT, editors; 1986; ICCAT, Madrid. pp. 277-284.

228. Goldberg SR, Mussiett DC (1984) Reproductive cycle of the Pacific bonito, *Sarda chilensis* (Scombridae), from northern Chile. Pacific Science 38: 228-231.

229. González N, Miranda M (1999) Edad y crecimiento de las especies: macarela (*Scomber japonicus*), sardina del sur (*Sardinops sagax*), pinchagua (*Opisthonema spp*) y chuhueco (*Cetengraulis mysticetus*) en el Ecuador. Boletin Cientifico y Tecnico, Instituto Nacional de Pesca de Ecuador 17: 1-20.

230. González-Garcés Santiso A (2002) Contribución al conocimiento de la dinámica de la población del atún blanco, *Thunnus alalunga* Bonnaterre, 1788 del Atlántico norte [Tesis Doctoral]. Madrid: Universidad Complutense de Madrid. 208 p.

231. Gordo LS, Martins MMB (1984) On some biological characteristics of mackerel (*Scomber scombrus* L.) from the west continental coast of Portugal. International Council for the Exploration of the Sea CM H:49.

232. Gordo LS, Martins MMB (1986) Sinopsis dos dados biológicos e estado de exploraçáo do stock de sarda *Scomber scombrus* L 1758, da costa continental Portuguesa. Boletim do Instituto Nacional de investigacao das Pescas 14: 29-57.

233. Gordo LS, Martins MMB, Jorge IM (1982) Preliminary study on the age and growth of mackerel (*Scomber scombrus* L.) in the ICES sub-area IX. International Council for the Exploration of the Sea CM H:16.

234. Govender A (1994) Growth of the king mackerel (*Scomberomorus commerson*) off the coast of Natal, South Africa from length and age data. Fisheries Research 20: 63-79.

235. Govender A (1995) Mortality and biological reference points for the king mackerel (Scomberomoues commerson) fishery off Natal, South Africa (based on a per-recruit assessment). Fisheries Research 23: 195–208.

236. Granados-Alcantar S (2002) Ciclo reproductivo del barrilete *Katsuwonus Pelamis* en el Oceano Pacifico Oriental [Maestro en Ciencias]. Mexico: Instituto Politecnico Nacional Centro Interdisciplinario de Ciencias Marinas. 68 p.

237. Grandcourt E, Al Abdessalaam TZ, Francis F, Al Shamsi AT, Al Ali S, et al. (2005) Assessment of the fishery for Kingfish (Kanaad/Khabat), *Scomberomorus commerson*, in the waters off Abu Dhabi Emirate. 34 p.

238. Grande M, Murua H, Zudaire I, Korta M (2010) Spawning activity and batch fecundity of skipjack, *Katsuwonus pelamis*, in the Western Indian Ocean. IOTC.

239. Greer-Walker M, Witthames P, Emerson L, Walsh M (1987) Estimation of fecundity in the western mackerel stock, 1986. International Council for the Exploration of the Sea CM H:41.

240. Grégoire F (1993) Biological characteristics of Atlantic mackerel (*Scomber scombrus* L.) sampled along the Canadian coast between 1983 and 1991. Mont-Joli, Quebec. 138 p.

241. Griffiths S, Pepperell J, Tonks M, Sawynok W, Olyott L, et al. (2010) Biology, Fisheries and Status of Longtail Tuna (Thunnus Tonggol), with Special Reference to Recreational Fisheries in Australian Waters. Final Report FRDC Project 2008/058.

242. Griffiths SP (2010) Stock assessment and efficacy of size limits on longtail tuna (*Thunnus tonggol*) caught in Australian waters. Fisheries Research 102: 248-257.

243. Griffiths SP, Fry GC, Manson FJ, Lou DC (2009) Age and growth of longtail tuna (*Thunnus tonggol)* in tropical and temperate waters of the central Indo-Pacific. ICES Journal of Marine Science 67: 125-134.

244. Griswold CA, Silverman MJ (1992) Fecundity of the Atlantic Mackerel (Scomber scombrus) in the Northwest Atlantic in 1987. . Journal of Northwest Atlantic Fishery Science 12: 35-40.

245. Grudtsev ME (1992) Particularites de repartition et caracteristique biologique de la melva *Auxis rochei* (Risso) dans les eaux du Sahara. Collective Volume of Scientific Papers, ICCAT 39: 284-288.

246. Grudtsev ME, Korolevich LI (1986) Studies of frigate tuna *Auxis thazard* (Lacepede) age and growth in the eastern part of the Equatorial Atlantic. Collective Volume of Scientific Papers, ICCAT 25: 269-274.

247. Guanco MR (1991) Growth and mortality on indian mackerel *Rastrelliger kanagurta* (Scombridae) in the Visayas Sea, central Philippines. Fishbyte 9: 13-15.

248. Gunn J, Polacheck T, Davis T, Klaer N, Cowling A, et al. (1998) Fishery indicators for the SBT stock: an update of 12 indicators first used in 1988 plus additional indicators from the 1990's. 1-25 p.

249. Gunn JS, Clear NP, Carter TI, Rees AJ, Stanley CA, et al. (2008) Age and growth in southern bluefin tuna, *Thunnus maccoyii* (Castelnau): Direct estimation from otoliths, scales and vertebrae. Fisheries Research 92: 207-220.

250. Hafiz A (1986) Skipjack fishery in the Maldives. FAO Indo-Pacific Tuna Development and Management Programme Collective volume of working documents presented at the third meeting of the working group on tunas in the EEZ of Maldives and Sri Lanka, 22-25 September 1986. Colombo, Sri Lanka. pp. 30-46.

251. Hajjej G, Hattour A, Allaya H, Jarboui O, Bouain A (2010) Biology of little tunny Euthynnus alletteratus in the Gulf of Gabes, Southern Tunisia (Central Mediterranean Sea). Revista de Biologia Marina y Oceanografia. Revista de Biologia Marina y Oceanografia 45: 399–406.

252. Hallier JP, Gaertner D (2006) Estimated growth rate of the skipjack tuna (*Katsuwonus pelamis*) from tagging surveys conducted in the Senegalese area (1996-1999) within a meta-analysis framework. Collective Volume of Scientific Papers, ICCAT 59: 411-420.

253. Hallier JP, Stéquert B, Maury O, Bard FX (2005) Growth of bigeye tuna (*Thunnus obesus*) in the eastern Atlantic Ocean from tagging-recapture data and otoliht readings. Collective Volume of Scientific Papers, ICCAT 57: 181-194.

254. Hamada T, Iwai S (1967) Biological studies on Sawara resources in Harima-Nada and adjacent waters-I. On some morphological characters and growth. Bulletin of the Japanese Society of Scientific Fisheries 33: 1013-1020.

255. Hamasaki S (1993) Age and growth of Japanese Spanish mackerel in the east China Sea and Yellow Sea. Bulletin of Seikai National Fisheries Research Institute (Japan) 71: 101-110.

256. Hampton J (1986) Effect of tagging on the condition of southeren bluefin tuna, *Thunnus maccoyii* (Castlenau). Australian Journal of Marine and Freshwater Research 37: 699-705.

257. Hampton J (1991) Estimation ot southern bluefin tuna *Thunnus maccoyii* growth parameters from tagging data, using von Bertalanffy models incorporating individual variation. Fishery Bulletin 89: 577-590.

258. Hampton J (1991) Estimation of southern bluefin tuna *Thunnus maccoyii* natural mortality and movement rates from tagging experiments. Fishery Bulletin 89: 591-610.

259. Hampton J (2000) Natural mortality rates in tropical tunas: size really does matter. Canadian Journal of Fisheries and Aquatic Sciences 57: 1002-1010.

260. Hansen JE (1987) Aspectos biológicos y pesqueros del bonito del Mar Argentino (Pisces, Scombridae, *Sarda sarda*). Collective Volume of Scientific Papers, ICCAT 26: 441-442.

261. Hansen JE (1988) Caracterización morfométrica y merística del bonito argentino. Revista de Investigación y Desarrollo Pesquero 8: 11- 18.

262. Hansen JE (1989) Crecimiento del "bonito" argentino (Pisces, Scombridae, *Sarda sarda*). Physis (A) 47: 13-19.

263. Hassani S, Stéquert B (1991) Sexual maturity, spawning and fecundity of the yellowfin tuna (*Thunnus albacares*) of the western Indian Ocean. FAO Indo-Pacific Tuna Development and Management Programme Collective volume of working documents presented at the Expert Consultation on Stock Assessment of Tunas in the Indian Ocean 2-6 July 1990. Bangkok, Thailand. pp. 91-107.

264. Hattour A (1984) Analyse de l'age, de la croissance et des captures des thons rouges (*Thunnus thynnus*) et des thonines (*Euthynnus alleteratus* L.) peches dans les eaux Tunisiennes. Bulletin de l'Institut National Scientifique et Technique d'Océanographie et de Pêche 11: 5-39.

265. Hattour A (2000) Contribution a l'etude des poissons pelagiques des eaux Tunisiennes [These de Doctorat]. Tunisie: Université de Tunis II. 327 p.

266. Hazin FHV, Hazin HG, Zagaglia CR, Travassos P, Júnior MFG (2001) Analyses des captures de la pêche à la senne réalisées par le ``B.P. Xixili´´ dans l 'Océan Atlantique équatorial. Collective Volume of Scientific Papers, ICCAT 52: 488-498.

267. Headley MD (2005) A preliminary study of the diet and other biological characteristics of the blackfin tuna (*Thunnus atlanticus*) in Tobago [Master Thesis]. Tobago: The University of the West Indies. 57 p.

268. Hearn WS, Polacheck T (2003) Estimating long-term growth-rate changes of southern bluefin tuna (*Thunnus maccoyii*) from two periods of tag-return data. Fishery Bulletin 101: 58-74.

269. Hennemuth RC (1959) Additional information on the length-weight relationship of skipjack tuna from the eastern tropical Pacific Ocean. Bulletin of the Inter-American Tropical Tuna Commission, IATTC 4: 23-37.

270. Hennemuth RC (1961) Size and year class composition of catch, age and growth of yellowfin tuna in the eastern tropical Pacific Ocean for the years 1954-1958. Bulletin of the Inter-American Tropical Tuna Commission, IATTC 5: 1-112.

271. Hogarth WT (1976) Life history aspects of the wahoo *Acanthocybium solandri* (Cuvier and Valenciennes) from the coast of North Carolina [Ph.D Thesis]. Raleigh, United States: North Carolina State University. 107 p.

272. Hongskul V (1974) Population dynamics of pla tu *Rastrelliger neglectus* (Van Kampen) in the Gulf of Thailand. Bangkok, Thailand. 297 p.

273. Hsu CC (1991) Parameters estimation of generalized von Bertalanffy growth equation. Acta Oceanographica Taiwanica 26: 66-77.

274. Hsu CC (1999) The length-weight relationship of albacore, *Thunnus alalunga*, from the Indian Ocean. Fisheries Research 41: 87-92.

275. Hsu CC, Liu HC, Wu CL, Huang ST, Liao HK (2000) New information on age composition and length–weight relationship of bluefin tuna, *Thunnus thynnus*, in the southwestern north Pacific. Fisheries Science 66: 485- 493.

276. Hu F, Yang RT (1972) A preliminary study on sexual maturity and fecundity of skipjack tuna. Journal of the Fisheries Society of Taiwan 1: 88-98.

277. Huang CS, Wu CL, Kuo CL, Su WC (1991) Age and growth of the Indian Ocean albacore, *Thunnus alalunga*, by scales. FAO Indo-Pacific Tuna Development and Management Programme Collective volume of working documents presented at the Expert Consultation on Stock Assessment of Tunas in the Indian Ocean 2-6 July 1990. Bangkok, Thailand. pp. 111-122.

278. Hunter JR, Macewicz BJ (1986) The spawning frequency of skipjack tuna, *Katsuwonus pelamis*, from the south Pacific. Fishery Bulletin 84: 895-903.

279. Hurley PCF, Iles TD (1983) Age and growth estimation of Atlantic bluefin tuna, *Thunnus thynnus*, using otoliths. NOAA-NMFS Technical Report 8: 71-75.

280. Hwang SD, Kim JY, Lee TW (2008) Age, growth, and maturity of chub mackerel off Korea. North American Journal of Fisheries Management 28: 1414-1425.

281. Hwang SD, Lee TW (2005) Spawning dates and early growth of chub mackerel *Scomber japonicus* as indicated by otolith microstructure of juveniles in the inshore nursery ground. Fisheries Science 71: 1185-1187.

282. IGFA (2010) Database of International Game Fish Association angling records until 2010. Fort Lauderdale, United States: IGFA.

283. Ingles J, Pauly D (1984) An atlas of the growth, mortality and recruitment of Philippines fishes. ICLARM Technical Report 13: 1-127.

284. Inoue T, Wada Y, Tojima T, Takeno K (2007) Age and migration of the Japanese Spanish mackerel (*Scomberomorus niphonius*) in the coastal waters of Kyoto prefecture. Bulletin of the Kyoto Institute of Oceanic and Fishery Science 29: 1-6.

285. IOTC (2006) Report of the ninth session of the scientific committee, 6-10 November 2006, Victoria, Seychelles. Victoria, Seychelles.

286. Isakov VI (1973) Growth and total mortality of mackerel from the new England area. International Commission for the Northwest Atlantic Fisheries Research Document 73: 1-7.

287. ISC (2006) Report of the ISC – Albacore Working Group Stock Assessment Workshop. National Research Institute of Far Seas Fisheries, 5-7-1, 28- November - 5 December 2006. Orido, Shizuoka, Japan. 60 p.

288. Itano DG (2000) The reproductive biology of yellowfin tuna (*Thunnus albacares*) in Hawaiian waters and the western tropical Pacific Ocean: Project summary. 1-69 p.

289. Ito RY, Hawn DR, Collette BB (1994) First record of the butterfly kingfish *Gasterochisma melampus* (Scombridae) from the north Pacific Ocean. Japanese Journal of Ichthyology 40: 482-486.

290. Ivanov LS. On the biology of the mackerel of the Black Sea (*Scomber scombrus* L.); 1966. pp. 97-134 (In Bulgarian).

291. Iversen ES, Yoshida HO (1957) Notes on the biology of the wahoo in the Line Islands. Pacific Science 11: 370-379.

292. Iversen SA, Adoff GR (1983) Fecundity observations on mackerel from the Norwegian coast. International Council for the Exploration of the Sea CM H:45.

293. Jabat M, Dalzell P (1988) Preliminary sotck assessment of the Danao ring net fishery for bullet tunas and small pelagic fishes in the Camotes Sea, central Visayas, Philippines. Bureau of Fisheries and Aquatic Resources Technical Paper Series 11: 1-38.

294. James PSBR, Pillai PP, Jayaprakash AA, Yohannan TM, Siraimeetan P, et al. (1992) Stock assessment of tunas from the Indian seas. Indian Journal of Fisheries 39: 260-277.

295. Jardim E, Santos A, Incom I, Bucal D, Paulo I, et al. (1998) Some biological parameters for several important species caught by the artisanal fleet of Guinea-Bissau.

296. Jenkins KLM, McBride RS (2009) Reproductive biology of wahoo, *Acanthocybium solandri*, from the Atlantic coast of Florida and the Bahamas. Marine and Freshwater Research 60: 893.

297. John ME, Reddy KSN (1989) Some considerations on the population dynamics of yellowfin tuna, *Thunnus albacares* (Bonnaterre) in Indian seas. FSI Special Publication 2: 33-54.

298. John ME, Sudarsan D (1993) Fishery and biology of yellowfin tuna occurring in oceanic fishery in Indian seas. In: Sudarsan D, John ME, editors. Tuna Research in India. Bombay: Fishery Survey of India. pp. 36-61.

299. Johnson AG, Fable WA, Barger LE, Williams ML (1980) Preliminary report on the age and growth ok king mackerel (*Scomberomorus cavalla*) from the United Sates. Collective Volume of Scientific Papers, ICCAT 9: 722-733.

300. Joseph J (1963) Fecundity of the yellowfin tuna (*Thunnus albacares*) and skipjack (*Katsuwonus pelamis*) from the eastern Pacific Ocean. Bulletin of the Inter-American Tropical Tuna Commission, IATTC 7: 255-292.

301. Joseph J, Calkins TP (1969) Population dynamics of the skipjack tuna (*Katsuwonus pelamis*) in the eastern Pacific Ocean. Bulletin of the Inter-American Tropical Tuna Commission, IATTC 13: 1-273.

302. Joseph L, Maldeniya R, Van der Knaap M (1987) Fishery and age and growth of kawakawa (*E. affinis*) and frigate tuna (*A. thazard*). FAO Indo-Pacific Tuna Development and Management Programme Collective volume of working documents presented at the Expert Consultation on stock assessment of tunas in the Indian Ocean 4-8 December 1986, Colombo, Sri Lanka. pp. 113-123.

303. Joshi KK, Abdussamad EM, Koya KPS, Sivadas M, Kuriakose S, et al. (2012) Fishery, biology and dynamics of dogtooth tuna, Gymnosarda unicolor (Rüppell , 1838) exploited from Indian seas. Indian Journal of Fisheries 59: 75–79.

304. Josse E, Le Guen JC, Kearney R, Lewis A, Smith A, et al. (1979) Growth of skipjack. Occasional paper-South Pacific Commission 11: 1-83.

305. Julien-Flüs M (1988) A study of growth parameters and mortality rates of *Scomberomorus brasiliensis* from the coastal areas of Trinidad, west Indies. In: Venema S, Christensen JM, Pauly D, editors. Contributions to tropical fisheries biology FAO/DANIDA Follow-up Training Course on Fish Stock Assessment in the Tropics, Denmark, 1986 and Philippines, 1987 FAO Fisheries Report No 389. Rome: FAO. pp. 385-400.

306. Kahraman AE (2005) Preliminary investigations on Atlantic black skipjack (*Euthynnus alletteratus* Raf.1810) in the eastern Mediterranean Sea. Collective Volume of Scientific Papers, ICCAT 58: 502-509.

307. Kahraman AE, Alicli TZ, Akayli T, Oray IK (2008) Reproductive biology of little tunny, *Euthynnus alletteratus* (Rafinesque), from the north-eastern Mediterranean Sea. Journal of Applied Ichthyology 24: 551-554.

308. Kahraman AE, Alicli TZ, Akayli T, Oray IK (2008) Reproductive biology of little tunny, *Euthynnus alletteratus* (Rafinesque), from the north-eastern Mediterranean Sea. Journal of Applied Ichthyology 24: 551–554.

309. Kahraman AE, Göktürk D, Bozkurt ER, Akaylı T, Karakulak S (2010) Some reproductive aspects of female bullet tuna, *Auxis rochei* (Risso), from the Turkish Mediterranean coasts. African Journal of Biotechnology 9: 6813–6818.

310. Kahraman AE, Göktürk D, Karakulak FS (2011) Age and growth of bullet tuna, *Auxis rochei* (Risso), from the Turkish Mediterranean coasts. African Journal of Biotechnology, 10, 3009–3013 10: 3009–3013.

311. Kahraman AE, Oray IK (2001) The determination of age and growth parameters of Atlantic little tunny *Euthynnus alleteratus* (Rafinesque, 1810) in Turkish waters. Collective Volume of Scientific Papers, ICCAT 52: 719-732.

312. Kakati VS, Chennappa Gowda N (2000) Record-sized mackerel, *Rastrelliger kanagurta* caught from Karwar waters on the west coast of India. Journal of the Marine Biological Association of India 41: 133-134.

313. Kara OF (1979) Observations on growth and relationship between length and weight of *Sarda sarda* (Bloch). Investigación Pesquera (Chile) 43: 95-105.

314. Karakulak S, Oray I, Corriero A, Aprea A, Spedicato D, et al. (2004) First information on the reproductive biology of the bluefin tuna (*Thunnus thynnus*) in the eastern Mediterranean. Collective Volume of Scientific Papers, ICCAT 56: 1158-1162.

315. Karpinski B, Hallier JP (1988) Preliminary results on yellowfin spawning in the western Indian Ocean. FAO Indo-Pacific Tuna Development and Management Programme Collective volume of working documents presented at the Expert Consultation on stock assessment of tunas in the Indian Ocean 22-27 December 1988. Mauritius. pp. 50-59.

316. Kästner D (1977) Preliminary results of the occurrence of two mackerel groups (*Scomber scombrus* L.) with different growth pattern west of Britain. International Council for the Exploration of the Sea CM H:38.

317. Kaymaram F, Darvishi M, Parafkandeh F, Ghasemi S, Talebzadeh SA (2011) Population dynamic parameters of *Thunnus tonggol* in the north of the Persian Gulf and Oman Sea.

318. Kaymaram F, Hossainy SA, Darvishi M, Talebzadeh SA, Sadeghi MS (2010) Reproduction and spawning patterns of the *Scomberomorus commerson* in the Iranian coastal waters of the Persian Gulf and Oman Sea. Iranian Journal of Fisheries Sciences 9: 233–244.

319. Kedidi SM, Fita NI, Abdulhadi A (1993) Population dynamics of the king seerfish *Scomberomorus commerson* along the Saudi Arabian Gulf coast. 19 p.

320. Khorshidian K, Carrara G (1993) An analysis of the length frequencies of *Thunnus tonggol* in Hormuzgan waters, Islamic Republic of Iran. FAO Indo-Pacific Tuna Development and Management Programme Collective volume of working documents presented at the Expert Consultation on Indian Ocean Tunas, 5th Session, 4-8 October 1993,TWS/93/2/4. Mahe, Seychelles. pp. 76-87.

321. Kikawa S (1962) Studies on the spawning activity of the Pacific tunas, Parathunnus mebachi and Neothunnus macropterus, by the gonad index examination. Nankai Regional Fisheries Research Laboratory 1: 43-56.

322. Kikawa S, Ferraro MG (1966) Maturation and spawning of tunas in the Indian Ocean. Proceedings of the Indo-Pacific Fisheries Council 12: 65-78.

323. Kimura R, Nashida K, Oozeki Y, Honda H (2002) Age determination method suitable for spotted mackerel *Scomber australasicus* using their otoliths. Bulletin of the Japanese Society of Fisheries Oceanography 66: 247-251.

324. Kiparissis S, Tserpes G, Tsimenidis N (2000) Aspects on the demography of chub mackerel (*Scomber japonicus* Houttuyn, 1782) in the Hellenic seas. Belgian Journal of Zoology 130: 3-7.

325. Kirkwood GP (1983) Estimation of von Bertalanffy growth curve parameters using both length increment and age-length data. Canadian Journal of Fisheries and Aquatic Sciences 40: 1405-1411.

326. Kishida T, Katsumi A (1989) Maturation and spawning of Japanese Spanish mackerel in the central and western waters of the Seto Inland Sea. Nippon Suisan Gakkaishi 55: 2065-2074.

327. Kishida T, Ueda K, Takao K (1985) Age and growth of Japanese Spanish mackerel in the central and western waters of the Seto Inland Sea. Bulletin of the Japanese Society of Scientific Fisheries 51: 529-537.

328. Klawe WL (1963) Observations on the spawning of four species of tuna, *Neothunnus macropterus*, *Katsuwonus pelamis*, *Auxis thazard*, and *Euthynnus lineatus*, in the eastern Pacific Ocean, based on the distribution of their larvae and juveniles. Bulletin of the Inter-American Tropical Tuna Commission, IATTC 9: 449-540.

329. Klawe WL, Calkins TP (1965) Length-weight relationship of black skipjack tuna, *Euthynnus lineatus*. California Fish and Game 51: 214-216.

330. Klima EF (1959) Aspects of the biology and the fishery for Spanish mackerel, *Scomberomorus maculatus* (Mitchill), of southern Florida. Coral Gables, Florida: Marine Laboratory,University of Miami. 40 p.

331. Knaggs EH, Parrish RH (1973) Maturation and growth of Pacific mackerel, *Scomber japonicus* Houttuyn. California Fish and Game 59: 114-120.

332. Kohno H (1994) Osteology and systematic position of the butterfly mackerel, *Gasterochisma melampus*. Japanese Journal of Ichthyology 31: 268-186.

333. Koido T, Suzuki Z (1989) Main spawning season of yellowfin tuna, *Thunnus albacares*, in the western tropical Pacific Ocean based on the gonad index. Bulletin of the Far Seas Fisheries Resesearch Laboratory 26: 153-164.

334. Kono N, Hanamura Y, Nishiyama Y, Fukuda M (1997) Changes in the age composition of Japanese Spanish mackerel, *Scomberomorus niphonius*, in the western Seto Inland Sea, Japan. Bulletin of the Nansei Regional Fisheries Research Laboratory 30: 1-8.

335. Koya KPS, Joshi KK, Abdussamad EM, Rohit P, Sivadas M, et al. (2012) Fishery, biology and stock structure of skipjack tuna, Katsuwonus pelamis (Linnaeus, 1758) exploited from Indian waters. Indian Journal of Fisheries. Indian Journal of Fisheries 59: 39–47.

336. Kramer D (1960) Development of eggs and larvae of Pacific mackerel and distribution and abundance of larvae 1952-56. Fishery Bulletin 60: 393-438.

337. Kramer SH (1986) Scombridae. In: Uchida RN, Uchiyama JH, editors. Fishery Atlas of the Northwestern Hawaiian Islands NOAA Technical Report NMFS 38. pp. 126-127.

338. Krishnamoorthi B (1958) Observations on the spawning season and the fisheries of the spotted seer, *Scomberomorus guttatus* (Bloch & Schneider). Indian Journal of Fisheries 5: 270-281.

339. Kromer JL, Insali P, Gomes M (1994) Rio Grande de Buba - Bio-ecologie et parametres environnementaux. Bissau: UICN/ Ministere des Peches de Guinee-Bissau. 118 p.

340. Ku JF, Tzeng WN (1985) Age and growth of spotted mackerel, *Scomber australasicus* (Cuvier), in the shelf waters of northeastern and southwestern Taiwan. Journal Fisheries Society of Taiwan 12: 12-26.

341. Kume S, Joseph J (1966) Size composition, growth and sexual maturity of bigeye tuna, *Thunnus obesus* (Lowe) from the Japanese long-line fishery in the eastern Pacific Ocean. Bulletin of the Inter-American Tropical Tuna Commission, IATTC 11: 47-49.

342. Kuo CM (1970) Taxonomic, growth, and maturation studies on the bonitos of the temperate eastern Pacific Ocean [Ph.D. Thesis]. San Diego: University of California. 321 p.

343. Labelle M (1991) Estimates of age and growth for south Pacific albacore. Taipei, Taiwan: National Taiwan University. 17 p.

344. Labelle M, Hampton J, Bailey K, Murray T, Fournier DA, et al. (1993) Determination of age and growth of south Pacific albacore (*Thunnus alalunga*) using three methodologies. Fishery Bulletin 91: 649-663.

345. Lablache G. Preliminary assessment for the Indian mackerel (*Rastrelliger kanagurta*) in Seychelles waters. In: Sanders MJ, Sparre P, Venema SC, editors; 1988.

346. Landau R (1965) Determination of age and growth rate in *Euthynnus alleteratus* and *E. affinis* using vertebrae. Rapports et Proces Verbaux des Reunions Publié par les Soins de Jean Furnesting Commission Internationale pour L’Exploration Scientifique de la Mediterranée 18: 241-244.

347. Laurs RM, Wetherall JA (1981) Growth rates of north Pacific albacore, *Thunnus alalunga*, based on tag returns. Fishery Bulletin 79: 293-302.

348. Lavapie-Gonzales F, Ganaden SR, Gayanilo FCJ (1997) Some population parameters of commercially-important fishes in the Philippines. Philippines: Bureau of Fisheries and Aquatic Resources. 114 p.

349. Le Guen JC, Baudin-Laurencin F, Champagnat C (1969) Croissance de l'albacore (*Thunnus albacares)* dans les régions de Pointe-Noire et de Dakar. Cahiers ORSTOM Série océanographie 7: 19-40.

350. Le Guen JC, Champagnac C (1968) Croissance des albacores dans les régions de Pointe-Noire et de Dakar. Document Scientifique Centre ORSTOM de Pointe-Noire 431: 1-25.

351. Le Guen JC, Sakagawa GT (1973) Apparent growth of yellowfin tuna from the eastern Atlantic Ocean. Fishery Bulletin 71: 175-187.

352. Lee LK, Yeh SY (1993) Studies on the age and growth of south Atlantic albacore (*Thunnus alalunga*) specimens collected from Taiwanese longliners. Collective Volume of Scientific Papers, ICCAT 40: 354-360.

353. Lee LK, Yeh SY (2007) Age and growth of south Atlantic albacore - a revision after the revelation of otolith daily ring counts. Collective Volume of Scientific Papers, ICCAT 60: 443-456.

354. Lee YC, Liu HC (1992) Age determination, by vertebra reading, in Indian albacore, *Thunnus alalunga* (Bonnaterre). Journal of the Fisheries Society of Taiwan 19: 89-102.

355. Lehodey P, Hampton J, Leroy B (1999) Preliminary results on age and growth of bigeye tuna (*Thunnus obesus*) from the western and central Pacific Ocean as indicated by daily growth increments and tagging data. Noumea, New Caledonia. 18 p.

356. Lehodey P, Leroy B (1999) Age and growth of yellowfin tuna (*Thunnus albacares*) from the western and central Pacific Ocean as indicated by daily growth increments and tagging data. Tahiti. 1-21 p.

357. Lema L, Macias D, Gómez-Vives MJ, {de La Serna} JM (2006) A preliminary approach to the Bullet tuna (Auxis rochei) fecundity in the Spanish Mediterranean. Collective Volume of Scientific Papers, ICCAT 59: 571-578.

358. Lessa R, Duarte-Neto P (2004) Age and growth of yellowfin tuna (*Thunnus albacares*) in the western equatorial Atlantic, using dorsal fin spines. Fisheries Research 69: 157-170.

359. Lessa RP, de Nóbrega MF, Bezerra-Junior JL (2004) Dinâmica de populaçoes e avaliaçao de estoques dos recursos pesqueiros da regiao nordeste. 274 p.

360. Lewis AD, Chapman LB, Sesewa A (1983) Biological notes on coastal pelagic fishes in Fiji. Suva, Fiji. 72 p.

361. Li G, Chen X, Feng B (2008) Age and growth of chub mackerel (*Scomber japonicus*) in the east China and Yellow seas using sectioned otolith samples. Journal of Ocean University of China (Oceanic and Coastal Sea Research) 7: 439-446.

362. Lieske E, Myers R (1994) Collins Pocket Guide. Coral reef fishes. Indo-Pacific & Caribbean including the Red Sea. Haper Collins Publishers. 1-400 p.

363. Lima JTAX, Fonteles-Filho AA, Chellappa S (2007) Biologia reproductiva da serra, *Scomberomorus brasiliensis* (osteichthyes: Scombridae), em águas costeiras do Rio Grande do Norte. Arquivos de Ciências do Mar 40: 24-30.

364. Lockwood SJ (1978) (1978) The fecundity of mackerel, Scomber scombrus L. International Council for the Exploration of the Sea CM, H:9. International Council for the Exploration of the Sea CM H:9: 1-5.

365. Lorenzo JM, González Pajuelo J (1996) Growth and reproductive biology of chub mackerel *Scomber japonicus* off the Canary Islands. South African Journal of Marine Science 17: 275-280.

366. Lorenzo JM, Pajuelo JG, Ramos AG (1995) Growth of the chub mackerel *Scomber japonicus* (Pisces: Scombridae) off the Canary Islands. Scientia Marina 59: 287-291.

367. Lorenzo-Nespereira JM, González-Pajuelo JM (1993) Determinación de la talla de primera madurez sexual y período reproductivo de la caballa *Scomber japonicus* (Houttuyn, 1782) de las Islas Canarias. Boletín del Instituto Español de Oceanografía 9: 15-21.

368. Lucano-Ramirez G, Ruiz-Ramirez S, Palomera-Sanchez FI, Gonzalez-Sanson G (2011) Reproductive biology of the Pacific sierra Scomberomorus sierra (Pisces, Scombridae) in the central Mexican Pacific. . Ciencias Marinas 37: 249–260.

369. Lucas C (1974) Working paper on southern bluefin tuna population dynamics. Collective Volume of Scientific Papers, ICCAT 111: 110-124.

370. Lucio P (1997) Biological aspects of mackerel (*Scomber scombrus* L. 1758) in the Bay of Biscay from the Basque country catches in the period 1987-1993. International Council for the Exploration of the Sea CM BB:9.

371. Luckhurst BE, Trott T (2000) Bermuda's commercial line fishery for wahoo and dolphinfish: Landings, seasonality and catch per unit effort trends

. Proceedings of the Gulf and Caribbean Fisheries Institute. Bermuda. pp. 404-413.

372. Lushkareva NF (1960) Data on the fecundity and on the development of the gonads of a mackerel. Okeanographia 46: 79-94.

373. Luther G (1973) Observations on the biology and the fishery of the Indian mackerel, *Rastrelliger kanagurta* (Cuvier) from Andaman Islands. Indian Journal of Fisheries 20: 425-447.

374. Macias D, Lema L, Gómez-Vives MJ, {de La Serna} JM (2005) Preliminary results on fecundity of atlantic bonito (Sarda sarda) caught in South Western Mediterranean Trap. Collective Volume of Scientific Papers, ICCAT 58: 1635-1645.

375. Macías D, Lema L, Gómez-Vives MJ, Ortiz de Urbina JM, de la Serna JM (2006) Some biological aspects of small tunas (*Euthynnus alletteratus*, *Sarda sarda* & *Auxis rochei*) from the south western Spanish Meditarraneam traps. Collective Volume of Scientific Papers, ICCAT 59: 579-589.

376. Mackie MC, Gaughan DJ, Buckworth RC (2003) Stock assessment of narrow-barred Spanish mackerel (*Scomberomorus commerson*) in western Australia. 242 p.

377. Mackie MC, Lewis PD, Gaughan DJ, Newman SJ (2005) Variability in spawning frequency and reproductive development of the narrow-barred Spanish mackerel (*Scomberomorus commerson*) along the west coast of Australia. Fishery Bulletin 103: 344-354.

378. Maldeniya R, Joseph L (1986) On the distribution and biology oy yellowfin tuna (*T. albacares*) from the western and southern coastal waters of Sri Lanka. Collective Volume of Working Document, IPTP 1: 51-61.

379. Manning MJ, Marriot PM, Taylor PR (2006) The length and age composition of the commercial catch of blue mackerel (*Scomber australasicus*) in EMA 1 during the 2002-03 fishing year, including a comparison with data collected during the 1997-98 fishing year, and some remarks on estimating blue mackerel ages from otoliths. 42 p.

380. Manooch CS, Naughton SP, Grimes CB, Trent L (1987) Age and growth of king mackerel, *Scomberomorus cavalla*, from the U.S. Gulf of Mexico. Marine Fisheries Review 49: 102-108.

381. Mansor MI. On the status of the Rastrelliger and Decapterus fisheries of the west coast of Peninsular Malaysia in 1984-1985; 1987; Colombo, Sri Lanka. pp. 81-100.

382. Mansor MI, Abdullah S (1995) Growth and mortality of Indian mackerel (*Rastrelliger kanagurta*) and slender scad (*Decapterus russelli*) off the east coast of Peninsular Malaysia. Scientia Marina 59: 533-457.

383. Marcille J, Champagnat C, Armada N (1978) Croissance du patudo (*Thunnus obesus*) de l'Océan Atlantique intertropical oriental. Document Centre de Recherches Océanologiques, Abidjan, ORSTOM 9: 73-81.

384. Martínez C, Böhm MG, Cerna F, Díaz E, Muñóz P, et al. (2007) Estudio biológico-pesquero de la caballa entre la I - X regiones. Chile. 1-416 p.

385. Martins MMB, Gordo LS (1984) On the comparison of Spanish mackerel (*Scomber japonicus* Houttuyn,1780) from Gorringe Bank and Peniche (Portuguese coast). International Council for the Exploration of the Sea CM H:50.

386. Martins MMB, Jorge IM, Gordo LS (1983) On the maturity, morphological characteristics and growth of *Scomber japonicus* Houttuyn, 1780 of west continental coast of Portugal. 21 p.

387. Mather FJ, Mason JM, Jones AC (1995) Historical document: life history and fisheries of Atlantic bluefin Tuna. NOAA Technical Memorandum, NMFS-SEFSC-370: 1-174.

388. Matsumoto T, Miyabe N (2002) Preliminary report on the maturity and spawning of bigeye tuna *Thunnus obesus* in the central Atlantic Ocean. Collective Volume of Scientific Papers, ICCAT 54: 246-260.

389. Maxwell WD (1977) Age composition of California barracuda, *Sphyraena argentea*; Pacific bonito, *Sarda chiliensis*; white seabass, *Cynoscion nobilis*; and yellowtail, *Seriola dorsalis* from southern California partyboats 1972-1974. Fort Bragg, California: California Department of Fish and Game, Marine Resources Region. 22 p.

390. Mayo CA (1973) Rearing, growth, and development of the eggs and larvae of seven scombrid fishes from the Straits of Florida [Ph.D thesis]. Gainesville, Florida: University of Florida. 138 p.

391. McBride RS, Richardson AK, Maki KL (2008) Age, growth, and mortality of wahoo, *Acanthocybium solandri*, from the Atlantic coast of Florida and the Bahamas. Marine and Freshwater Research 59: 799-807.

392. McIlwain JL, Claereboudt MR, Al-Oufi HS, Zaki S, Goddard JS (2005) Spatial variation in age and growth of the kingfish (*Scomberomorus commerson*) in the coastal waters of the Sultanate of Oman. Fisheries Research 73: 283-298.

393. McPherson GR (1991) Reproductive biology of yellowfin tuna in the eastern australian fishing zone, with special reference to the north-western Coral Sea. Australian Journal of Marine and Freshwater Research 42: 465-477.

394. McPherson GR (1992) Age and growth of the narrow-barred Spanish mackerel (*Scomberomorus commerson* Lacépède, 1800) in north-eastern Queensland waters. Australian Journal of Marine and Freshwater Research 43: 1269-1282.

395. McPherson GR (1993) Reproductive biology of the narrow barred Spanish Mackerel (*Scomberomorus commerson* Lacepede,1800) in Queensland waters. Asian Fisheries Science 6: 169-182.

396. Medina A, Abascal FJ, Megina C, García A (2002) Stereological assessment of the reproductive status of female Atlantic northern bluefin tuna during migration to Mediterranean spawning grounds through the Strait of Gibraltar. Journal of Fish Biology 60: 203-217.

397. Medina-Gómez SP (2006) Edad y crecimiento de la sierra del Pacífico *Scomberomorus Sierra* (Jordan y Starks, 1895), en el Golfo de California, México [Tesis de Maestria]. La Paz: Instituto Politécnico Nacional, Centro Interdisciplinario de Ciencias Marinas. 51 p.

398. Medina-Quej A, Domínguez-Viveros M (1997) Edad y crecimiento del *Scomberomorus maculatus* (Scombriformes: Scombridae) en Quintana Roo, México. Revista de Biologia Tropical 45: 1155-1161.

399. Megalofonou P (1990) First age estimates of albacore, Thunnus alalunga Bonn, in the Aegean Sea using scales. Rapports et Procès-Verbaux des Réunions Commission Internationale pour l'Exploration Scientifique de la Mer Mediterranée 32: 1-268.

400. Megalofonou P (1991) Size distribution, length-weight relationships, age and sex of albacore, *Thunnus alalunga* Bonn., in the Aegean Sea. FAO Fisheries Report No 449: 197-213.

401. Megalofonou P (2000) Age and growth of Mediterranean albacore. Journal of Fish Biology 57: 700-715.

402. Mehanna SF (2001) Population dynamics and fisheries management of Indian mackerel *Rastrelliger kanagurta* in the Gulf of Suez, Egypt. Journal of King Abdulaziz University - Marine Sciences 12: 217-229.

403. Mejuto J, González Garcés A (1984) Relación talla-peso de atún blanco juvenil del Atlántico norte. Collective Volume of Scientific Papers, ICCAT 23: 278-281.

404. Mendes BB, Fonseca P, Campos A (2004) Weight – length relationships for 46 fish species of the Portuguese west coast. Journal of Applied Ichthyology 20: 355-361.

405. Mendizabal y Oriza D (1987) Análisis preliminar del estado de la población de sierra, *Scomberomorus maculatus* (Mitchill), del Golfo de México (Período 1973-1976) [Tesis para obtener el título de biólogo]. México: Universidad Nacional Autónoma de México. 127 p.

406. Mendo J (1984) Age, growth and some reproductive and feeding aspects of the Peruvian mackerel. Boletín del Instituto del Mar de Peru 8: 104-156.

407. Menezes MF, Pessoa Aragao L (1977) Aspectos da biometria e biologia do bonito, *Euthynnus alletteratus* (Rafinisque), no estado do Ceará, Brasil. Arquivos de Ciências do Mar 17: 95-100.

408. Menz A, Pizarro S (1988) The fishery, biology and bionomics of the Pacific mackerel (*Scomber japonicus*, Houttuyn 1782) in Ecuador. Boletin Cientifico y Tecnico, Instituto Nacional de Pesca de Ecuador 9: 16-48.

409. Moazzam M, Badar Osmany H, Zohra K (2005) Indian mackerel (*Rastrelliger kanagurta*) from Pakistan-I. Some aspects of biology and fisheries. Records Zoological Survey of Pakistan 16: 58-75.

410. Mohamad-Kasim H, Ameer Hamsa KMS (1989) On the fishery and population dynamics of seerfish *Scomberomorus commerson* (Lacepede) off Tuticorin (Gulf of Mannar). Central Marine Fisheries Research Institute Bulletin 44: 46-53.

411. Mohamad-Kasim HC, Muthiah C, Pillai NGK, Yohannan TM, Manojkumar B, et al. (2002) Stock assessment of seerfishes in the Indian seas. In: Pillai NGK, Menon NG, Pillai PP, Ganga U, editors. Management of Scombroid Fisheries: Kochi, India, Central Marine Fisheries Research Institute. pp. 108-124.

412. Mohan M, Kunhikoya KK (1964) Spawning biology of skipjack, *Katsuwonus pelamis* (Linnaeus) from Minicoy waters. Bulletin Central Marine Fisheries Research Institute, Cochin 36: 149-154.

413. Monte S (1964) Observações sobre a estrutura histológica das gônadas da albacora, *Thunnus atlanticus* (Lesson), no nordeste do Brasil. Boletim do Instituto de Biologia Marinha da Universidade Federal do Rio Grande do Norte 1: 17-31.

414. Moore HL (1951) Estimation of age and growth of yellowfin tuna (*Neothunnus macropterus*) in Hawaiian waters by size frequencies. Fishery Bulletin 52: 131-149.

415. Moores JA, Winters GH, Parsons LS (1975) Migrations and biological characteristics of Atlantic mackerel (*Scomber scombrus*) occurring in Newfoundland waters. Journal of the Fisheries Research Board of Canada 32: 1347-1357.

416. Morales-Nin B (1988) Crecimiento del *Scomber japonicus* (Houyttun,1872) (Pisces: Scombridae) y *Sardinops sagax* (Jenyns,1923) (Pisces: Clupeidae) en aguas ecuatorianas. Investigación Pesquera (Chile) 52: 483-500.

417. Morales-Nin B (1989) Growth determination of tropical marine fishes by means of otolith interpretation and length frequency analysis. Aquatic Living Resources 2: 241-253.

418. Morrison M, Taylor P, Marriott P, Sutton C (2001) An assessment of information on blue mackerel (*Scomber australasicus*) stocks. New Zealand Fisheries Assessment Report 2001/44. 26 p.

419. Morse WW (1980) Spawning and fecundity of Atlantic mackerel, *Scomber scombrus*, in the middle Atlantic Bight. Fishery Bulletin 78: 103-108.

420. Motlagh SAT, Shojaei MG (2009) Population dynamics of narrow-barred Spanish mackerel (*Scomberornorus commerson*) in the Persian Gulf, Bushehr province, Iran. Indian Journal of Fisheries 56: 7-11.

421. Moutopoulos DK, Stergiou KI (2002) Length-weight and length-length relationships of fish species from the Aegean Sea (Greece). Journal of Applied Ichthyology 18: 200-203.

422. Murayama T, Mitani I, Aoki I (1995) Estimation of the spawning period of the Pacific mackerel *Scomber japonicus* based on the changes in gonad index and the ovarian histology. Bulletin of the Japanese Society of Fisheries Oceanography 59: 11-17.

423. Murphy GI (1977) New understanding of southern bluefin tuna. Australian Fisheries 36: 2-6.

424. Murray PA, Joseph WB (1996) Trends in exploitation of the wahoo, *Acanthocybium solandri*, by the St. Lucian pelagic fishery. Proceedings of the Gulf and Caribbean Fisheries Institute 44: 737-746.

425. Murray PA, Sarvay WB (1987) Use of ELEFAN programs in the estimation of growth parameters of the wahoo, *Acanthocybium solandri*, caught off St. Lucia, West Indies. Fishbyte 5: 14-15.

426. Muthiah C (1985) Maturation and spawning of *Euthynnus affinis*, *Auxis thazard* and *Auxis rochei* in the Mangalore inshore area during 1979-82. Bulletin Central Marine Fisheries Research Institute, Cochin 36: 71-85.

427. Muus BJ, Nielsen JG (1999) Sea fish. Scandinavian Fishing Year Book. Denmark: Hedehusene. 1-340 p.

428. Mwebaza-Ndawula L (1990) Seasonal variation in abundance of the Indian mackerel, *Rastrelliger kanagurta* Cuvier (Pisces: Scombridae) along the Zanzibar coast of east Africa. Hydrobiologia 190: 233-239.

429. Nagai T, Takeda Y, Nakamura Y, Shinohara M, Ueta Y, et al. (1996) Stock status of Spanish mackerel, *Scomberomorus niphonius*, in the eastern Seto Inland Sea, Japan. Bulletin of the Nansei Regional Fisheries Research Laboratory 29: 19-26.

430. Naik SK, Tiburtius A, Bhalkar SR (1998) Biology of the seerfish landed by exploratory trawlers. Indian Journal of Fisheries 45: 35-41.

431. Nakamura EL, Uchiyama JH. Length-weight relations of Pacific tunas. In: Manar TA, editor; 1966. Honolulu, Hawaii. pp. 197-201.

432. Nakamura I (1990) Scombridae. In: Gon O, Heemstra PC, editors. Fishes of the Southern Ocean: J.L.B. Smith Institute of Ichthyology, Grahamstown, South Africa. pp. 404-405.

433. Natarajan R, Bensam P (1978) Eggs and early larvae of the Indian mackerel, *Rastrelliger kanagurta* (Cuvier) from nearshore waters of Porto Novo. Current Science 47: 829-830.

434. Nava-Ortega RA, Espino-Barr E, Gallardo-Cabello M, Puente-Gómez M, Cabral-Solís EG (2012) Growth analysis of the Pacific sierra Scomberomorus sierra in Colima, México. Revista de Biologia Marina y Oceanografia 47: 273–281.

435. Neilson JD, Campana SE (2008) A validated description of age and growth of western Atlantic bluefin tuna (*Thunnus thynnus*). Canadian Journal of Fisheries and Aquatic Sciences 65: 1523-1527.

436. Neja Z (1992) Maturation and fecundity of Mackerel (*Scomber scombrus* L.) in northwest Atlantic. Acta Ichthyologia et Piscatoria 22: 125-140.

437. Niiya Y (2001) Maturation cycle and batch fecundity of the bullet tuna *Auxis rochei* off Cape Ashizuri, southwestern Japan. Nippon Suisan Gakkaishi 67: 10-16.

438. Niiya Y (2001) Age, growth, maturation and life of bullet tuna *Auxis rochei* in the Pacific waters off Kochi prefecture. Nippon Suisan Gakkaishi 67: 429-437.

439. Nikaido H, Miyabe N, Ueyanagi S (1991) Spawning time and frequency of bigeye tuna,*Thunnus obesus*. Bulletin of the National Research Institute of Far Seas Fisheries 28: 47-73.

440. Noble A, Gopakumar G, Gopalakrishna-Pillai N, Kulkarni GM, Narayana-Kurup K, et al. (1992) Assessment of mackerel stock along the Indian coast. Indian Journal of Fisheries 39: 119-124.

441. Nóbrega MF, Lessa RP (2009) Age and growth of the king mackerel (*Scomberomorus cavalla*) off the northeastern coast of Brazil. Brazilian Journal of Oceanography 57: 273-285.

442. Nomura H (1967) Dados biológicos sobre a serra *Scomberomorus maculatus* (Mitchill), das águas Cearenses. Arquivo da Estação de Biologia Marinha, Universidade Federal Ceara 7: 29-39.

443. Nomura H, de Sousa Rodrigues MS (1967) Biological notes on king mackerel, *Scomberomorus cavalla* (Cuvier), from northeastern Brazil. Arquivo da Estação de Biologia Marinha, Universidade Federal Ceara 7: 79-85.

444. Nootmorn P (2004) Reproductive biology of bigeye tuna in the eastern Indian Ocean. IOTC Proceedings 7: 1-5.

445. Nootmorn P, Yakoh A, Kawises K (2005) Reproductive biology of yellowfin tuna in the eastern Indian Ocean. Phuket, Thailand. 8 p.

446. Nurhakim S (1995) Population dynamics of ikan banyar (*Rastrelliger kanagurta*) in the Java Sea. In: Potier M, Nurhakim S, editors. BIODYNEX : Biology, Dynamics, Exploitation of the Small Pelagic Fishes in the Java Sea: AARD/ORSTOM. pp. 109-123.

447. Nzioka RM (1991) Population characteristics of kingfish *Scomberomorus commerson*, in inshore waters of Kenya. FAO Indo-Pacific Tuna Development and Management Programme Collective volume of working documents presented at the Expert Consultation on Stock Assessment of Tunas in the Indian Ocean 2-6 July 1990. Bangkok, Thailand. pp. 200-207.

448. O'Brien L, Burnett J, Mayo RK (1993) Maturation of nineteen species of finfish off the northeast coast of the United States, 1985-1990. 1-66 p.

449. O'Driscoll RL, McClatchie S (1997) Spatial distribution of planktivorous fish schools in relation to krill abundance and local hidrography off Otago, New Zeland. Deep-Sea Research II 45: 1295-1325.

450. Oliva-López J, González L (1987) Fecundidad parcial de la caballa de Chile (*Scomber japonicus*) 1987. Santiago, Chile. 18 p.

451. Orange CJ (1961) Spawning of yellowfin tuna and skipjack in the eastern tropical Pacific, as inferred from studies of gonad development. Bulletin of the Inter-American Tropical Tuna Commission, IATTC 5: 459-526.

452. Oray IK, Karakulak FS, Zengin M (2004) Report on the Turkish bonito (*Sarda sarda*) fishery in 2000/2001. Collective Volume of Scientific Papers, ICCAT 56: 784-788.

453. Ortiz de Zárate V, Cummings-Parrack N, Rodriguez-Cabello C (1994) New tag-recapture growth analysis for north Atlantic albacore data. Collective Volume of Scientific Papers, ICCAT 42: 395-401.

454. Ortiz de Zárate V, Parrack NC (1996) Note on updated tag-recapture growth analyses for north Atlantic albacore. Collective Volume of Scientific Papers, ICCAT 43: 247-253.

455. Ortiz de Zárate V, Restrepo V (2001) Analysis of tagging data from north Atlantic albacore: von Bertalanffy growth estimates and catch-at-age. Collective Volume of Scientific Papers, ICCAT 52: 1435-1446.

456. Ortiz M, Palmer C (2008) Review and estimates of von Bertalanffy growth curves for the king mackerel Atlantic and Gulf of Mexico stock units.

457. Ostapenko AT (1988) Age, croissance et caracteristiques morphologiques du maquereau espagnol (*Scomber japonicus* Houtt.) de l'Atlantique sud-est. Collection of Scientific Papers International Commission for the Southeast Atlantic Fisheries 15: 161-174.

458. Otsu T, Hansen R (1962) Sexual maturity and spawning of the albacore in the central south Pacific Ocean. Fishery Bulletin 62: 151-162.

459. Ouchi A (1978) Studies on the age and growth of common mackerel, *Scomber japonicus,* in the waters west of Kyushu and east of Tsushima Islands. Bulletin of Seikai Regional Fisheries Research Laboratory (Japan) 51: 97-110.

460. Pagavino M, Gaertner D (1995) Ajuste de una curva de crecimiento a frecuencias de tallas de atún listado (*Katsuwonus pelamis*) pescado en el Mar Caribe suroriental. Collective Volume of Scientific Papers, ICCAT 44: 303-309.

461. Pardo AS, Oliva JL (1992) Estimación de la talla de primera madurez sexual de caballa (*Scomber japonicus peruanus*) en la zona norte de Chile durante el período de máxima actividad reproductiva. Investigación Pesquera (Chile): 97-106.

462. Parks W, Bard FX, Cayré P, Kume S, Santos-Guerra A (1982) Length-weight relations for bigeye tuna captured in the eastern Atlantic Ocean. Collective Volume of Scientific Papers, ICCAT 17: 214-225.

463. Parrack ML, Phares PL (1979) Aspects of the growth of Atlantic bluefin tuna determined from mark-recapture data. Collective Volume of Scientific Papers, ICCAT 8: 356-366.

464. Pathansali D (1962) A preliminary report on the Rastrelliger fishery in Malaya. Proceedings of the Indo-Pacific Fisheries Council 9: 37-48.

465. Pathansali D (1967) Observations on the gonad maturity stages of female *Rastrelliger kanagurta* (Cuvier). Proceedings of the Indo-Pacific Fisheries Council 12: 116-123.

466. Pauly D, Aung S (1984) Population dynamics of some fishes of Burma based on length-frequency data. Rome: FAO. 22 p.

467. Pauly D, de Vildoso AC, Mejia J, Samamé M, Palomares ML (1987) Population dynamics and estimated anchoveta consumption of bonito (*Sarda chiliensis*) off Peru, 1953 to 1982. In: Pauly D, Tsukayama I, editors. The Peruvian anchoveta and its upwelling ecosystem: three decades of changes. Makati, Metro Manila, Philippines: ICLARM studies and Reviews 15. pp. 248-267.

468. Peña N, Alheit J, Nakama ME (1986) Fecundidad parcial de la caballa del Peru (*Scomber japonicus peruanus*). Boletín del Instituto del Mar del Peru 10: 91-104.

469. Penney AJ (1994) Morphometric relationships, annual catches and catch-at-size for South African caught south Atlantic albacore (*Thunnus alalunga*). Collective Volume of Scientific Papers, ICCAT 42: 371-382.

470. Pereira J (1984) Croissance du patudo (*Parathunnus obesus*) de l'Atlantique. Collective Volume of Scientific Papers, ICCAT 21: 143-154.

471. Perrotta RG (1992) Growth of mackerel (*Scomber japonicus* Houttuyn, 1782) from the Buenos Aires-north Patagonian region (Argentine Sea). Scientia Marina 56: 7-16.

472. Perrotta RG, Carvalho N, Isidro E (2005) Comparative study on growth of chub mackerel (*Scomber japonicus* Houttuyn,1782) from three different regions: NW Mediterranean, NE and SW Atlantic. Revista de Investigación y Desarrollo Pesquero 17: 67-79.

473. Perrotta RG, Christiansen HE (1990) Estimación de la frecuencia reproductiva y algunas consideraciones acerca de la pesca de la caballa (*Scomber japonicus*) en relación con el comportamiento de los cardúmenes. Physis (Buenos Aires) Seccion A,: 1-14.

474. Perrotta RG, Forciniti L (1988) Sobre la edad y el crecimiento de la caballa (*Scomber japonicus*) del área marplatense. Revista de Investigación y Desarrollo Pesquero 8: 19-32.

475. Perrotta RG, Forciniti L (1994) Un análisis del crecimiento de la caballa (*Scomber japonicus*) en dos áreas de su distribución. Frente Maritimo 15: 101-109.

476. Perrotta RG, Forciniti L, Cousseau MB, Hansen JE (1990) Parte I. Cálculo de los parámetros de crecimiento, estimación de tasas de mortalidad y análisis de otros aspectos biológicos del efectivo marplatense. Período Enero de 1980 - Diciembre de 1985. Contribución - Instituto Nacional de Investigación y Desarrollo Pesquero Informe Técnico. pp. 43-65.

477. Pillai NGK, Pillai PP, Said-Koya KP, Sathianandan TV (1996) Assessment of the stock of kingseer, *Scomberomorus commerson* (Lacepede), along the west coast of India. In: Anganuzzi AA, Stobberup KA, Webb NJ, editors. FAO Indo-Pacific Tuna Development and Management Programme Collective volume of working documents presented at the Expert Consultation on Indian Ocean Tunas, 25-29 September 1995. Colombo, Sri Lanka. pp. 299-311.

478. Pillai PP, Pillai NGK, Muthiah C, Yohannan TM, Mohamad Kasim H, et al. (2002) Stock assessment of coastal tunas in the Indian Seas. In: Pillai NGK, Menon NG, Pillai PP, Ganga U, editors. Management of Scombroid Fisheries. Kochi: Central Marine Fisheries Research Institute. pp. 125-130.

479. Pillai PP, Pillai NGK, Muthiah C, Yohannan TM, Mohamad-Kasim H, et al. (2002) Status of exploitation of coastal tunas in the Indian seas. In: Pillai NGK, Menon NG, Pillai PP, Ganga U, editors. Management of Scombroid Fisheries: Central Marine Fisheries Research Institute, Kochi, India.

480. Pillai PP, Pillai NGK, Sathianandan TV, Elayathu MNK (1994) Fishery biology and stock assessment of *Scomberomorus commerson* (Lacepede) from the south-west coast of India. FAO Indo-Pacific Tuna Development and Management Programme Collective volume of working documents presented at the Expert Consultation on Indian Ocean Tunas, Mahe, Seychelles, 4-8 October 1993. Colombo, Sri Lanka. pp. 55-61.

481. Pillay PP, Gopakumar G (1989) Stock assessment of migratory fish species based on localized data - oceanic skipjack tuna pole and line fishery at Minicoy as a case study. Contributions to tropical fish stock assessment in India FAO/DANIDA/ICAR National Follow-up Training Course on Fish Stock Assessment, 2 - 28 November. Cochin. pp. 127-142.

482. Pizarro de Rodríguez S (1983) Estudio preliminar sobre la edad y crecimiento del *Scomber japonicus* Houttuyn en aguas ecuatorianas. Revista de Ciencias Marinas y Limnologia 2: 79-95.

483. Pó LA, Dionísio C, de Paula e Silva R (1992) Growth of skipjack *Katsuwonus pelamis* from Mozambique. Revista de Investigação Pesqueira (Maputo) 21: 98-105.

484. Polacheck T, Eveson JP, Laslett GM (2004) Increase in growth rates of southern bluefin tuna (*Thunnus maccoyii*) over four decades: 1960 to 2000. Canadian Journal of Fisheries and Aquatic Sciences 61: 307-322.

485. Postel E (1954) Comparaison entre la taille des mâles et des femelles. Taille de première maturité chez *Euthynnus alliteratus* (Raf.). Bulletin de la Societe Scientifique de Bretagne 29: 155-157.

486. Postel E (1955) Contribution à l'étude de la biologie de quelques Scombridae de l'Atlantique tropico-oriental [These de Doctorat]. France: Université de Rennes. 1-167 p.

487. Postel E (1956) Essai sur la Palomette *Orcynopsis unicolor* (Geoffroy Saint-Hilaire, 1809). Bulletin de l'Institut Fondamental d'Afrique Noire Série A Sciences Naturelles 18: 1220-1248.

488. Powell D (1975) Age, growth, and reproduction in Florida stocks of Spanish mackerel, *Scomberomorus maculatus*. Florida Marine Research Publications 5: 1-21.

489. Prabhakar A, Dudley RG (1989) Age, growth and mortality rates of longtail tuna *Thunnus tonggol* (Bleeker) in Omani waters based on length data. FAO Indo-Pacific Tuna Development and Management Programme Report of the Workshop on Tunas and Seerfishes in the Arabian Sea Region, IPTP/89/GEN/16, February 1989. Muscat, Oman. pp. 90-96.

490. Quiñones-Velázques C (2006) Dinámica Poblacional de la Sierra del Golfo Scomberomorus Concolor (Lockington, 1879) y de la Sierra del Pacífico Scomberomorus Sierra (Jordan y Starks, 1895) en el Golfo de California.

491. Quiñónez-Velázquez C (2007) Biología y dinámica poblacional de *Scomberomurus concolor* en el Golfo de California. 1-3 p.

492. Quiñónez-Velázquez C, Gluyas-Millán MG (1996) Evidence of different stocks of Mackerel, *Scomber japonicus*. Ciencias Marinas 22: 377-395.

493. Rafail SZ (1972) Studies of Red Sea fisheries by light and purse-seine near Al-Ghardaqa. Bulletin of the Institute of Oceanography and Fisheries (Cairo) 2: 25-49.

494. Rafail SZ (1972) A statistical study of length-weight relationship of eight Egyptian fishes. Bulletin of the Institute of Oceanography and Fisheries (Cairo) 2: 136-156.

495. Raju G. Studies on the spawning of the oceanic skipjack *Katsuwonus pelamis* (Linnaeus) in Minicoy waters; 1964; Mandapam Camp, India. pp. 744-768.

496. Ramírez-Arredondo I (1990) Aspectos biométricos de la carachana pintada, *Euthynnus alletteratus* (Pisces: Scombridae) de los alrededores de la Isla Picua, Edo, Sucre, Venezuela. Boletin del Instituto Oceanografico de Venezuela 29: 141-151.

497. Ramírez-Arredondo I (1993) Aspectos reproductivos de la carachana pintada, *Euthynnus alletteratus* (Pisces:Scombridae) de los alrededores de la Isla de Picua, Estado Sucre, Venezuela. Boletin del Instituto Oceanografico de Venezuela 32: 69-78.

498. Ramírez-Arredondo I, Silva J, Marchán F (1996) Relación longitud peso y factor de condición en *Euthynnus alletteratu*s (Rafinesque 1810), (Pisces: Scombridae) de los alrededores de las Islas los Testigos, Venezuela. Boletin del Instituto Oceanografico de Venezuela 35: 63-68.

499. Ramon D, Bailey K (1996) Spawning seasonality of albacore, *Thunnus alalunga* in the south Pacific Ocean. Fishery Bulletin 94: 725-733.

500. Rao KVN. An account of the ripe ovaries of some Indian tunas; 1964; Mandapam Camp, India. pp. 733-743.

501. Rao VR (1967) Spawning behaviour and fecundity of the Indian mackerel, *Rastrelliger kanagurta* (Cuvier), at Mangalore. Indian Journal of Fisheries 14: 171-186.

502. Ratty FJ, Michael-Laurs R, Kelly RM (1989) Gonad morphology, histology and spermatogenesis in south Pacific albacore tuna *Thunnus alalunga* (Scombridae). Fishery Bulletin 88: 207-216.

503. Restrepo VR, Diaz GA, Walter JF, Neilson JD, Campana SE, et al. (2011) Updated estimate of the growth curve of Western Atlantic bluefin tuna. Aquatic Living Resources 23: 335–342.

504. Rey JC, Aloi E, Ramos A (1986) Growth of the Atlantic bonito (*Sarda sarda* Bloch, 1793) in the Atlantic and Mediterranean area of the Strait of Gibraltar. Investigación Pesquera (Chile) 50: 179-185.

505. Rey JC, Alot E, Ramos A (1984) Synopsis biológica del bonito, *Sarda sarda* (Bloch) del Mediterraneo y Atlántico este. Collective Volume of Scientific Papers, ICCAT 20: 469-502.

506. Richards WJ, Bullis HR (1978) Status of the konwledge on the biology and resources of the blackfin tuna, Thunnus atlanticus (Pisces, Scombridae). Collective Volume of Scientific Papers, ICCAT 7: 130-141.

507. Rodríguez-Cabello C, Restrepo VR, Rodríguez-Marín E, Cort JL, de la Serna JM (2007) Estimation of northeast Atlantic bluefin tuna (*Thunnus thynnus*) growth parameters from tagging data. Collective Volume of Scientific Papers, ICCAT 60: 1258-1264.

508. Rodríguez-Roda J (1964) Biología del atún,*Thunnus thynnus* (L), de la costa sudatlántica de España. Investigación Pesquera (Chile) 25: 33-146.

509. Rodríguez-Roda J (1966) Estudio de la bacoreta, *Euthynnus alletteratus* (Raf.), bonito, *Sarda sarda* (Bloch) y melva, *Auxis thazard* (Lac.), capturados por las almadrabas españolas. Investigación Pesquera (Chile) 30: 247-292.

510. Rodríguez-Roda J (1967) Fecundidad del atún, *Thunnus thynnus* (L.), de la costa sudatlántica de España. Investigación Pesquera (Chile) 31: 33-52.

511. Rodríguez-Roda J (1979) Edad y crecimiento de la bacoreta *Euthynnus alletteratus* (Raf.) de la costa sudaltántica de España. Investigación Pesquera (Chile) 43: 591-599.

512. Rodríguez-Roda J (1981) Estudio de la edad y crecimiento del bonito, *Sarda Sarda* (Bloch), de la costa sudatlántica de España. Investigación Pesquera (Chile) 45: 181-186.

513. Rodríguez-Roda J (1982) Biología de la caballa (o estornino), *Scomber (Pneumatophorus) japonicus* Houttuyn (1782) del Golfo de Cádiz. Investigación Pesquera (Chile) 41: 143-259.

514. Rodríguez-Roda J (1983) Edad y crecimiento de la melva, *Auxis rochei* (Risso), del sur de España. Investigación Pesquera (Chile) 47: 397-402.

515. Roedel P (1938) Record-size mackerel in Santa Monica Bay. California Fish and Game 24: 423.

516. Rohit P, Gupta AC (2004) Fishery, biology and stock of the Indian mackerel *Rastrelliger kanagurta* off Mangalore-Malpe in Karnataka, India. Journal of the Marine Biological Association of India 46: 185-191.

517. Rohit P, Rao GS, Rammohan K (2012) Age, growth and population structure of the yellowfin tuna Thunnus albacares (Bonnaterre, 1788) exploited along the east coast of India. Indian Journal of Fisheries 59: 1-6.

518. Romanov EV, Korotkova LP (1988) Age and growth rates of the yellow-fin tuna (*Thunnus albacares*) (Bonnaterre, 1978) (Pisces, Scombridae) in the north-western part of the Indian Ocean, determined by counting the rings of vertebrae. FAO Indo-Pacific Tuna Development and Management Programme Collective volume of working documents presented at the Expert Consultation on Stock Assessment of Tunas in the Indian Ocean 22-27 June 1988. Moka, Mauritius. pp. 68-73.

519. Romeo T, Azzurro E, Mostarda E (2005) Record of *Acanthocybium solandri* in the central Mediterranean Sea , with notes on parasites. Journal of the Marine Biological Association of the United Kingdom 85: 1295-1296.

520. Ronquillo IA (1963) A contribution to the biology of Philippine tunas. FAO Fisheries Report No 6: 1683-1752.

521. Ronquillo T (1964) Results of studies on the biology of tunas. Science Review 5: 60-65.

522. Rothschild BJ (1963) Skipjack ecology. In: van Campen WG, editor. Progress in 1961-1962 US Fish Wildlife Service Circular. pp. 13-17.

523. Rothschild BJ (1967) Estimates of the growth of skipjack tuna (*Katsuwonus pelamis*) in the Hawaiian Islands. Proceedings of the Indo-Pacific Fisheries Council 12: 100-111.

524. Rudomiotkina GP (1984) New data on reproduction of Auxis spp in the Gulf of Guinea. Collective Volume of Scientific Papers, ICCAT 20: 465-468.

525. S.C. T, V.R. R (1994) Areview of the growth rate ofWest Atlantic bluefin tuna, *Thunnus thynnus*, estimated from marked and recaptured fish. Collective Volume of Scientific Papers, ICCAT 42: 170-172.

526. Sabatés A, Recasens L (2001) Seasonal distribution and spawning of small tunas (*Auxis rochei* and *Sarda sarda*) in the northwestern Mediterranean. Scientia Marina 65: 95-100.

527. Sadeghi MS, Kaymaram F, Jamili S, Fatemi MR, Mortazavi MS (2009) Patterns of reproduction and spawning of the *Scomberomorus commerson* in the coastal waters of Iran. Journal of Fisheries and Aquatic Science 4: 32-40.

528. Sadhotomo B, Banon-Atmadja dS (1985) On the growth of some small pelagic fishes in the Java Sea. Jurnal Penelitian Perikanan Laut (Journal of Marine Fisheries Research) 33: 53-60.

529. Sanders MJ, Kedidi SM (1984) Stock assessment for the Indian mackerel (*Rastrelliger kanagurta*) caught by purse seine from the Gulf of Suez and more southern Red Sea waters. Cairo. 28 p.

530. Santamaria N, Bello G, Corriero A, Deflorio M, Vassallo-Agius R, et al. (2009) Age and growth of Atlantic bluefin tuna, *Thunnus thynnus* (Osteichthyes: Thunnidae), in the Mediterranean Sea. Journal of Applied Ichthyology 25: 38-45.

531. Santamaria N, Sion L, Cacucci M, De Metrio G (1998) Eta` ed accrescimento di *Sarda sarda* (Bloch, 1793) (Pisces, Scombridae) nello Ionio settentrionale. Biologia Marina Mediterranea 5: 721-725.

532. Santana JC, Delgado de Molina A, Ariz J (1993) Estimación de una ecuación talla-peso para *Acanthocybiun solandri* (Cuvier, 1832), capturado en la Isla de el Hierro (Islas Canarias). Collective Volume of Scientific Papers, ICCAT 40: 401-405.

533. Santiago J (1992) Application of "Multifan" to estimate the age composition of the north Atlantic albacore catches. Collective Volume of Scientific Papers, ICCAT 39: 188-195.

534. Santiago J (1993) A new length-weight relationship for the north Atlantic albacore. Collective Volume of Scientific Papers, ICCAT 40: 316-319.

535. Santiago J (2004) Dinámica de la población de atún blanco (*Thunnus alalunga*, Bonaterre 1788) del Atlántico norte [Tesis Doctoral]. España: Universidad del País Vasco. 354 p.

536. Sanzo L (1933) Uova e primi stadi larvali di alalonga (*Orcynus germo* Ltkn.). R Comitato Talassografico Italiano 198: 1-11.

537. Sato Y (1990) Common mackerel (*Scomber japonicus* Houttuyn) of the Pacific: its ecology and fishing activities. Marine and Freshwater Behaviour and Physiology 17: 15-65.

538. Schaefer KM (1987) Reproductive biology of black skipjack, *Euthynnus lineatus*, an eastern Pacific Tuna. Bulletin of the Inter-American Tropical Tuna Commission, IATTC 19: 169-260.

539. Schaefer KM (1996) Spawning time, frequency, and batch fecundity of yellowfin tuna, *Thunnus albacares*, from Clipperton Atoll in the eastern Pacific Ocean. Fishery Bulletin 94: 98-112.

540. Schaefer KM (1998) Reproductive biology of yellowfin tuna (*Thunnus albacares)* in the eastern Pacific Ocean. Bulletin of the Inter-American Tropical Tuna Commission, IATTC 21: 205-272.

541. Schaefer KM, Fuller DW (2006) Estimates of age and growth of bigeye tuna (*Thunnus obesus*) in the eastern Pacific Ocean, based on otolith increments and tagging data. Bulletin of the Inter-American Tropical Tuna Commission, IATTC 23: 35-77.

542. Schaefer KM, Fuller DW, Miyabe N (2005) Reproductive biology of bigeye tuna (*Thunnus obesus*) in the eastern and central Pacific Ocean. Bulletin of the Inter-American Tropical Tuna Commission, IATTC 23: 1-35.

543. Schaefer MB, Orange CJ (1956) Studies of the sexual development and spawning of yellowfin tuna (*Neothunnus macropterus*) and skipjack (*Katsuwonus pelamis*) in three areas of the eastern Pacific Ocean, by examination of gonads. Bulletin of the Inter-American Tropical Tuna Commission, IATTC 1: 283-349.

544. Schmidt DJ, Collins MR, Wyanski DM (1993) Age, growth, maturity, and spawning of Spanish mackerel, *Scomberomorus maculatus* (Mitchill), from the Atlantic coast of the southeastern United States. Fishery Bulletin 91: 526-533.

545. Schneider W (1990) FAO species identification sheets for fishery purposes. Field guide to the commercial marine resources of the Gulf of Guinea. Prepared and published with the support of the FAO Regional Office for Africa. Rome: FAO. 268 p.

546. Schultze DL, Collins RA (1977) Age composition of California landings of bluefin tuna, *Thunnus thynnus*, 1963 through 1969. 1-44 p.

547. Secor DH, Wingate RL, Neilson JD, Rooker JR, Campana SE (2008) Growth of Atlantic bluefin tuna: direct age estimates. ICCAT, SCRS/2008/084: 1-14.

548. Sekharan KV (1958) On the south Kanara coastal fishery for mackerel, *Rastrelliger kanagurta* (Cuvier) together with notes on the biology of the fish. Indian Journal of Fisheries 5: 1-31.

549. Serventy DL (1956) Additional observations on the biology of the northern bluefin tuna, *Kishinoella tonggol* (Bleeker), in Australia. Australian Journal of Marine and Freshwater Research 7: 44-63.

550. Sette OE (1950) Biology of the Atlantic mackerel (*Scomber scombrus*) of north America. Part II - Migrations and habits. Fishery Bulletin 49: 250-358.

551. Shimose T, Tanabe T, Chen KS, Hsu CC (2009) Age determination and growth of Pacific bluefin tuna, *Thunnus orientalis,* off Japan and Taiwan. Fisheries Research 100: 134-139.

552. Shingu C (1970) Studies relevant to distribution and migration of the southern bluefin tuna. Bulletin of the Far Seas Fisheries Resesearch Laboratory 3: 57-114.

553. Shingu C (1978) Ecology and stock of southern bluefin tuna. Japan Association of Fishery Resources Protection Fishery Study 31: 88.

554. Shiraishi T, Ketkar SD, Katoh Y, Nyuji M, Yamaguchi A, et al. (2009) Spawning frequency of the Tsushima Current subpopulation of chub mackerel Scomber japonicus off Kyushu, Japan. Fisheries Science 75: 649-655.

555. Shiraishi T, Okamoto K, Yoneda M, Sakai T, Ohshimo S, et al. (2008) Age validation, growth and annual reproductive cycle of chub mackerel *Scomber japonicus* off the waters of northern Kyushu and in the east China Sea. Fisheries Science 74: 947-954.

556. Shomura RS. Age and growth studies of four species of tunas in the Pacific Ocean. In: Manar TA, editor; 1966; Honolulu, Hawaii. Bureau of Commercial Fisheries, Biological Laboratory, Honolulu, Hawaii. pp. 203-219.

557. Shomura RS, Keala BA (1963) Growth and sexual dimorphism in growth of bigeye tuna (*Thunnus obesus*) a preliminary report. FAO Fisheries Report No 2: 1409-1417.

558. Shuford RL, Dean JM, Stéquert B, Morize E (2007) Age and growth of yellowfin tuna in the Atlantic Ocean. Collective Volume of Scientific Papers, ICCAT 60: 330-341.

559. Sibert JR, Kearney RE, Lawson TA (1987) Variations de croissance des bonites marquees (*Katsuwonus pelamis*). Nouméa: South Pacific Commission. 43 p.

560. Silas EG (1963) Synopsis of biological data on double-lined mackerel *Grammatorcynus bicarinatus* (Quoy and Gaimard) (Indo-Pacific). FAO Fisheries Biology synopsis No 72: 811-833.

561. Silas EG. Aspects of the taxonomy and biology of the oriental bonito *Sarda orientalis* (Temminck and Schlegel); 1964. pp. 283-308.

562. Silas EG, Pillai PP (1985) Exploratory fishing by oceanic drift gillnetting and purse seining in the Lakshadweep. Bulletin Central Marine Fisheries Research Institute, Cochin 36: 165-175.

563. Silas EG, Pillai PP, Srinath M, Jayaprakash AA, Muthiah C, et al. (1985) Population dynamics of tunas: stock assessment. Bulletin Central Marine Fisheries Research Institute, Cochin 36: 20-27.

564. Simmons DC (1969) Maturity and spawning of skipjack tuna (*Katsuwonus pelamis*) in the Atlantic Ocean, with comments on nematode infestation of the ovaries. Miami, USA: U.S. Dept. of the Interior, Bureau of Commercial Fisheries. 17 p.

565. Sinovčić G (2001) Population structure, reproduction, age and growth of Atlantic mackerel, *Scomber scombrus* L. in the Adriatic Sea. Acta Adriatica 42: 85-92.

566. Sinovčić G, Franičević M, Zorica B, Cikes-Keč V (2004) Length-weight and length-length relationships for 10 pelagic fish species from the Adriatic Sea (Croatia). Journal of Applied Ichthyology 20: 156-158.

567. Sivadas M, Abdussamad EM, Jasmine S, Rohit P, Koya KPS, et al. (2012) Assessment of the fishery and stock of striped bonito, Sarda orientalis (Temminck and Schlegel, 1844) along Kerala coast with a general description of its fishery from Indian coast. Indian Journal of Fisheries 59: 57–61.

568. Sivadas M, Pillai PP, Ganga U (2002) Stock assessment of the oceanic skipjack, *Katsuwonus pelamis* in Minicoy, Lakshadweep. In: Pillai NGK, Menon NG, Pillai PP, Ganga U, editors. Management of Scombroid Fisheries: CMFRI; Kochi, Kochi. pp. 131-138.

569. Sivasubramaniam K (1966) Distribution and length-weight relationships of tunas and tuna-like fishes around Ceylon. Bulletin of the Fisheries Research Station Ceylon 19: 27-46.

570. Skagen DW (1989) Growth patterns in the North Sea and western mackerel in Norwegian catches 1960-1985. International Council for the Exploration of the Sea CM H:21: 1-21.

571. Sommer C, Schneider W, Poutiers JM (1996) FAO species identification field guide for fishery purposes. The living marine resources of Somalia. Rome: FAO. 1-376 p.

572. Sousa MI (1992) Seasonal growth of five commercially important fishes at Sofala Bank, Mozambique. Revista de Investigação Pesqueira (Maputo) 21: 79-97.

573. Sousa MI, Gislason H (1985) Reproduction, age and growth of the Indian mackerel, *Rastrelliger kanagurta* (Cuvier,1816) from Sofala Bank, Mozambique. Revista de Investigação Pesqueira (Maputo) 14: 1-28.

574. Staicu I, Maxim C (1974) Observations sur la biologie et la dynamique du maquereau espagnol (*Scomber japonicus* colias Gmelin) dans l'Atlantique centre-est. Cercetari Marine/Recherches Marines 7: 113-128.

575. Stéquert B, Conand F. Age and growth of bigeye tuna (*Thunnus obesus*) in the western Indian Ocean; 2003. pp. 1-17.

576. Stéquert B, Panfili J, Dean J (1996) Age and growth of yellowfin tuna, *Thunnus albacares*, from the western Indian Ocean, based on otolith microstructure. Fishery Bulletin 94: 124-134.

577. Stéquert B, Ramcharrun B (1995) La fécondité du listao (*Katsuwonus pelamis)* de l'ouest de l'oceán Indien. Aquatic Living Resources 8: 79-89.

578. Stéquert B, Ramcharrun R (1996) La reproduction du listao (*Katsuwonus pelamis*) dans le bassin ouest de l'océan Indien. Aquatic Living Resources 9: 235-247.

579. Stevens JD, Hausfeld HF, Davenport SR (1984) Observations on the biology, distribution and abundance of *Trachurus declivis*, *Sardinops neopilchardus* and *Scomber australasicus* in the Great Australian Bight. Cronulla, Australia: Commonwealth Scientific and Industrial Research Organization, Marine Laboratories. 27 p.

580. Stewart J, Ferrell DJ (2001) Age, growth, and commercial landings of yellowtail scad (*Trachurus novaezelandiae*) and blue mackerel (*Scomber australasicus*) off the coast of New South Wales, Australia. New Zealand Journal of Marine and Freshwater Research 35: 541-551.

581. Stobo WT, Hunt JJ (1974) Mackerel biology and history of the fishery in subarea 4. 23 p.

582. Sturm MGdL (1978) Aspects of the biology of *Scomberomorus maculatus* (Mitchill) in Trinidad. Journal of Fish Biology 13: 155-172.

583. Sturm MGdL, Salter P (1989) Age, growth, and reproduction of the king mackerel *Scomberomorus cavalla* (Cuvier) in Trinidad waters. Fishery Bulletin 88: 361-370.

584. Suarez-Caabro JA, Duarte-Bello PP (1961) Biología pesquera del bonito (*Katsuwonus pelamis)* y la albacora (*Thunnus atlanticus*) en Cuba. La Habana: Instituto Cubano de Investigaciones Tecnológicas. 151 p.

585. Sucondhamarn P, Tantisawetrat C, Sriruangcheep U (1970) Estimation of age and growth of chub mackerel *Rastrelliger neglectus* (van Kampen) in the western Gulf of Thailand. In: Marr JC, editor. The Kuroshio: a symposium on the Japanese current: East West Center Press, Honolulu. pp. 471-480.

586. Suda A, Kume S (1967) Survival and recruit of bigeye tuna in the Pacific Ocean, estimated by the data of tuna longline catch. Report of Nankai Regional Fisheries Research Laboratory 25: 91-103.

587. Sudarshan D, John ME, Nair KNV (1991) Some biological considerations of yellowfin tuna, Thunnus albacares (Bonnaterre) taken by longline gear in the Indian EEZ. FAO Indo-Pacific Tuna Development and Management Programme Workshop on stock assessment of yellowfin tuna in the Indian Ocean, TWS/91/11. Colombo, Sri Lanka. pp. 18-28.

588. Sudjastani T (1974) The species of *Rastrelliger* in the Java Sea, their taxonomy, morphometry and population dynamics [Master Thesis]. Vancouver: University of British Columbia. 155 p.

589. Sun C-L, Chu S-L, Yeh S-Z (2006) Reproductive biology of bigeye tuna in the western and central Pacific Ocean. Manila, Philippines. 1-22 p.

590. Sun C-L, Wang W-R, Yeh S (2005) Reproductive biology of yellowfin tuna in the central and western Pacific Ocean. Noumea, New Caledonia. 14 p.

591. Sun CL, Huang CL, Yeh S, Z (2001) Age and growth of the bigeye tuna, *Thunnus obesus*, in the western Pacific Ocean. Fishery Bulletin 99: 502-509.

592. Sutter III FC, Williams RO, Godcharles MF (1991) Growth and mortality of king mackerel *Scomberomorus cavalla* tagged in the southeastern United States. Fishery Bulletin 89: 733-737.

593. Sutthakorn P, Saranakomkul R. Biological aspects of chub mackerels (*Rastrelliger spp.*) and round scads (*Decapterus spp.*) on the weat coast of Thailand; 1987; Colombo, Sri Lanka. pp. 48-80.

594. Taghavi Motlagh SA, Hashemi SA, Kochanian P (2010) Population biology and assessment of kawakawa (Euthynnus affinis) in coastal waters of the Persian Gulf and Sea of Oman (Hormozgan Province). Iranian Journal of Fisheries Sciences 9: 315–326.

595. Takemori H, Sakamoto H, Ueda Y, Yamazaki H, Iwamoto A (2005) Growth of spanish mackerel *Scomberomorus niphonius* in the eastern Seto Inland Sea. Saibai Giken 32: 35-41.

596. Tampubolon GH (1988) Growth and mortality estimation of Indian mackerel (*Rastrelliger kanagurta*) in the Malacca Strait, Indonesia. In: Venema S, Moller-Christensen J, Pauly D, editors. Contributions to tropical fisheries biology FAO/DANIDA Follow-up Training Course on Fish Stock Assessment in the Tropics, Denmark, 1986 and Philippines, 1987 FAO Fisheries Report 389. Rome: FAO. pp. 372-384.

597. Tampubolon GH, Sedana-Merta IG. Mackerels fisheries in the Malacca Straits; 1987; Colombo, Sri Lanka. pp. 101-116.

598. Tan EO. Notes on the biology of chub mackerel *Rastrelliger brachysoma* (Bleeker), in Manila Bay. In: Marr Jc, editor; 1970. East- West Center Press, Honolulu. pp. 614.

599. Tanaka S (2006) Maturation of bluefin tuna in the Sea of Japan. Shimizu, Japan. 16-20 p.

600. Tandog-Edralin D, Ganaden SR, Fox P (1988) A comparative study of fish mortality rates in moderately and heavily fished areas of the Philippines. Contributions to tropical fisheries biology FAO/DANIDA Follow-up Training Course on Fish Stock Assessment in the Tropics, Denmark, 1986 and Philippines, 1987 FAO Fisheries Report No 389. Rome, Italy. pp. 468-481.

601. Tandog-Edralin DD, Cortes-Zaragoza EC, Dalzell P, Pauly D (1990) Some aspects of the biology of skipjack (*Katsuwonus pelamis*) in Philippine waters. Asian Marine Biology 7: 15-29.

602. Tankevich PB (1982) Age and growth of the bigeye tuna, *Thunnus obesus* (Scombridae) in the Indian Ocean. Journal of Ichthyology 22: 26-31.

603. Tantivala C (2000) Some biological study of yellowfin tuna (*Thunnus albacares*) and bigeye tuna (*Thunnus obesus*) in the eastern Indian Ocean. IOTC Proceedings No 3, WPTT00-30. Samuthprakarn, Thailand. pp. 436-440.

604. Taquet M, Reynal L, Laurans M, Lagin A (2000) Blackfin tuna (*Thunnus atlanticus*) fishing around FADs in Martinique (French West Indies). Aquatic Living Resources 13: 259-262.

605. Taylor PR (2002) A summary of information on blue mackerel (*Scomber australasicus*), characterisation of its fishery in QMAs 7,8, and 9, and recommendations on appropriate methods to monitor the status of this stock. Wellington, New Zealand. 68 p.

606. Thayer BD (1973) The status of the Pacific bonito resource and its management. California: California Department of Fish and Game. 1-16 p.

607. Thorogood J (1986) Aspects of the reproductive biology of the southern bluefin tuna *(Thunnus maccoyii)*. Fisheries Research 4: 297-315.

608. Thorogood J (1987) Age and growth rate determination of southern bluefin tuna, *Thunnus maccoyii*, using otolith banding. Journal of Fish Biology 30: 7-14.

609. Timohina OI, Romanov EV. Characteristics of ovogenesis and some data on maturation and spawning of skipjack tuna, *Katsuwonus pelamis* (Linnaeus, 1758), from the western part of the equatorial zone of the Indian Ocean. In: Anganuzzi AA, Stobberup KA, Webb NJ, editors; 1993. Indo-Pacific Tuna Development and Management Programme, Colombo, Sri Lanka. pp. 247-257.

610. Turner SC, Restrepo VR, Eklund AM (1991) A review of the growth of Atlantic bluefin tuna, *Thunnus thynnus*. Collective Volume of Scientific Papers, ICCAT 35: 271-293.

611. Uchiyama JH, Boggs CH (2006) Length-weight relationships of dolphinfish, *Coryphaena hippurus*, and wahoo, *Acanthocybium solandri:* seasonal effects of spawning and possible migration in the central north Pacific. Marine Fisheries Review 68: 19-29.

612. Uchiyama JH, Struhsaker P (1981) Age and growth of skipjack tuna, *Katsuwonus pelamis*, and yellowfin tuna, *Thunnus albacares*, as indicated by daily growth increments of sagittae. Fishery Bulletin 79: 151-162.

613. Udupa KS, Krishna B (1984) Age-and-growth equation of the Indian mackerel from purse-seine catches off Karnataka coast. Indian Journal of Fisheries 31: 61-67.

614. Ueyanagi S (1957) Spawning of the albacore in the western Pacific. Report of Nankai Regional Fisheries Research Laboratory 6: 113-124.

615. Valdovinos-Jacobo LA (2006) Edad, crecimiento y mortalidad de la sierra del golfo *Scomberomorus concolor* (Lockington, 1879) en el Golfo de California [Maestro en Ciencias]. Mexico: Instituto Politecnico Nacional. 70 p.

616. Valdovinos-Jacobo LA, Quiñónez-Velázquez C, Montemayor-López G (2006) Edad y crecimiento la sierra del Golfo *Scomberomorus concolor* (Lockington, 1879) en el Golfo de California. In: Espino-Barr E, Carrasco-Águila MA, Puente Gómez M, editors. III Foro Científico de Pesca Ribereña, Memorias. Puerto Vallarta, Jalisco: Sagarpa. pp. 39-40.

617. Valle-Gómez SV (1992) Caracterización de los cardúmenes de listado (*Katsuwonus pelamis*) y atún de aleta negra (*Thunnus atlanticus*) en aguas de Cuba. Collective Volume of Scientific Papers, ICCAT 39: 12-26.

618. van der Elst RP, Collette BB (1984) Game fishes of the east coast of southern Africa. 2. Biology and systematics of the queen mackerel *Scomberomorus plurilineatus*. 12 p.

619. Vasconcelos JA, Conolly PC (1980) A study of some biological aspects of the fishing of blackfin tuna (*Thunnus atlanticus*, Lesson) in the State of Rio Grande do norte Brazil. Collective Volume of Scientific Papers, ICCAT 9: 734-738.

620. Vasconcelos-Gesteira TC, Lobo-de Mesquita AL (1976) Época de reproduçao, tamanho e idade na primeira desova da Cavala e da serra, na costa do estado do Ceará (Brasil). Arquivos de Ciências do Mar 16: 83-86.

621. Velasco EM, Del Arbol J, Baro J, Sobrino I (2011) Age and growth of the Spanish chub mackerel *Scomber colias* off southern Spain: a comparison between samples from the NE Atlantic and the SW Mediterranean. Revista de Biologia Marina y Oceanografia 46: 27–34.

622. Vieira KR, Lins Oliveira JE, Barbalho MC, Garcia J (2005) Reproductive characteristics of blackfin tuna *Thunnus atlanticus* (Lesson, 1831) in northeast Brazil. Collective Volume of Scientific Papers, ICCAT 58: 1629-1634.

623. Vieira MHSR (1991) Saison de ponte et sex-ratio des albacores captures au Cap Vert. Collective Volume of Scientific Papers, ICCAT 36: 564-586.

624. Vijayaraghavan P (1955) Life-history and feeding habits of the spotted seer *Scomberomorus guttatus* (Bloch & Schneider). Indian Journal of Fisheries 2: 360 - 372.

625. Villamor B, Abaunza P, Celso-Fariña A (2004) Growth variability of mackerel (*Scomber scombrus*) off north and northwest Spain and a comparative review of the growth patterns in the northeast Atlantic. Fisheries Research 69: 107-121.

626. von Seckendorff RW, Zavala-Camin LA (1985) Reproduçao, crescimento e distribuiçao da cavalinha (*Scomber japonicus)* no sudeste e sul do Brasil. Boletim do Instituto de Pesca 12: 1-13.

627. Walker MG, Witthames PR, Bautista de los Santos JI (1994) Is the fecundity if the Atlantic mackerel (*Scomber scombrus*: Scombridae) determinate? Sarsia 79: 13-26.

628. Walsh M (1983) Investigations on the fecundity of North Sea mackerel. International Council for the Exploration of the Sea CM H:48.

629. Wang CY (1987) Studies on the fishery biology of striped bonito *Sarda orientalis* in the eastern waters of Taiwan. (I) Size composition, gonadosomatic indices and sex ratio. Bulletin of Taiwan Fisheries Research Institute 42: 67-76.

630. Wang Y, Liu Q (2006) Estimation of natural mortality using statistical analysis of fisheries catch-at-age data. Fisheries Research 78: 342-351.

631. Wankowski JWJ (1981) Estimated growth of surface-schooling skipjack tuna, *Katsuwonus pelamis*, and yellowfin tuna, *Thunnus albacares*, from the Papua New Guinea region. Fishery Bulletin 79: 517-532.

632. Warashina I, Hisada K (1972) Geographical distribution and body length composition of two tuna-like fishes, *Gasterochisma melampus* Richardson and *Allothunnus fallai* Serventy, taken by Japanese tuna longline fishery. Bulletin of the Far Seas Fisheries Research Laboratory 6: 51-75.

633. Ward TM, Rogers PJ (2007) Development and evaluation of egg-based stock assessment methods for blue mackerel *Scomber australasicus* in southern Australia. 250 p.

634. Watanabe C, Yatsu A (2006) Long-term changes in maturity at age of chub mackerel (*Scomber japonicus*) in relation to population declines in the waters off northeastern Japan. Fisheries Research 78: 323-332.

635. Watanabe T (1970) Morphology and ecology of early stages of life in Japa­nese common mackerel, Scomber japonicus Houttuyn, with special reference to fluctuation of population. Bulletin of the Tokai Regional Fisheries Research Laboratory 62: 1-283.

636. Watson JJ, Priede IG, Witthames PR, Owori-Wadunde A (1992) Batch fecundity of Atlantic mackerel, *Scomber scombrus* L. Journal of Fish Biology 40: 591-598.

637. Weber E (1980) An analysis of Atlantic bigeye tuna (*Thunnus obesus*) growth. Collective Volume of Scientific Papers, ICCAT 9: 303-307.

638. Westhaus-Ekau P, Ekau W (1982) Preliminary report of the investigations on cavala (*Scomber japonicus*) and chicharro (*Trachurus picturatus)* at the Department of Oceanography and Fisheries, Horta. Horta. 24 p.

639. Westman JR, Gilbert PW (1941) Notes on the age determination and growth of the Atlantic bluefin tuna, *Thunnus thynnus* (Linnaeus). Copeia 2: 70-72.

640. Wetherall JA, Michael Laurs R, Nishimoto RN, Yong MYY (1987) Growth variation and stock structure in north Pacific albacore. 10th North Pacific Albacore Workshop , 11-13 August 1987 , Far Seas Fisheries Research Laboratoty, Shimizu, Shizuoka, Japan.

641. Wheeler JFG, Ommaney FD (1953) Report on the Mauritius-Seychelles Fisheries Survey 1948-1949. London. 148 p.

642. White TF (1982) The Philippine tuna fishery and aspects of the population dynamics of tunas in Philippine waters. Colombo, Sri Lanka. 64 p.

643. Whitley GP (1964) Scombroid fishes of Australia and New Zealand. Proceedings of the Symposium on Scombridae Fishes Marine Biological Association of India Symposium Series. pp. 221-254.

644. Wild A (1986) Growth of yellowfin tuna, *Thunnus albacares*, in the eastern Pacific Ocean based on otolith increments. Bulletin of the Inter-American Tropical Tuna Commission, IATTC 18: 421-482.

645. Williams F (1964) The scombroid fishes of east Africa. Proceedings of the Symposium on Scombridae Fishes Marine Biological Association of India Symposium Series. pp. 107-167.

646. Williamson GR (1970) Little Tuna *Euthynnus affinis* in the Hong Kong area. Bulletin of the Japanese Society of Scientific Fisheries 36: 9-18.

647. Wilson MA (1981) The biology, ecology and exploitation of longtail tuna, *Thunnus tonggol* (Bleeker) in Oceania [M.Sc. Thesis]. Sydney, Australia: School of Biological Sciences, Macquarie University. 195 p.

648. Wolfe DC, Webb BF (1975) Slender Tuna (Allothunnus fallai Servently): First Record of Bulk Catches, Tasmania, 1974. Australian Journal of Marine and Freshwater Research 26: 213-221.

649. Wu CC (1987) Study on the biology of skipjack tuna, *Katsuwonus pelamis* in the eastern waters of Taiwan (1) Length frequency, group maturity and sex ratio. Bulletin of Taiwan Fisheries Research Institute 42: 33-50.

650. Wu CL, Kuo CL (1993) Maturity and fecundity of albacore, *Thunnus alalunga* (Bonnaterre), from the Indian Ocean. Journal of the Fisheries Society of Taiwan 20: 135-152.

651. Wu ZQ, Qiu SY, Yan SY (2000) Characters of reproductive biology of six pelagic fishes in Minnan-Taiwan Bank fishing ground. Marine Science Bulletin 19: 25-29.

652. Yabuta Y, Yukinawa M (1957) Age and growth of yellowfin tuna (*Neothunnus macropterus*) in Japanese waters by size frequencies. Report of Nankai Regional Fisheries Research Laboratory 5: 127-133.

653. Yabuta Y, Yukinawa M (1959) Growth and age of the yellowfin tuna (*Neothunnus macropterus*) in the equatorial Pacific. Report of Nankai Regional Fisheries Research Laboratory 11: 77-87.

654. Yabuta Y, Yukinawa M (1963) Growth and age of albacore. Report of Nankai Regional Fisheries Research Laboratory 17: 111-120.

655. Yabuta Y, Yukinawa M, Warashina Y (1960) Growth and age of yellowfin tuna. I. Age determination (Scale method). Report of Nankai Regional Fisheries Research Laboratory 12: 63-74.

656. Yamada T, Aoki I, Mitani I (1998) Spawning time, spawning frequency and fecundity of Japanese chub mackerel, *Scomber japonicus* in the waters around the Izy Islands, Japan. Fisheries Research 38: 83-89.

657. Yang RT (1970) Studies of age and growth of Atlantic albacore and a critical review on the stock structure. China Fisheries Monthly 213: 3-16.

658. Yang RT, Nose Y, Hiyama Y (1969) A comparative study on the age and growth of yellowfin tunas from the Pacific and Atlantic oceans. Bulletin of the Far Seas Fisheries Research Laboratory 2: 1-21.

659. Yao M (1981) Growth of skipjack tuna in the western Pacific Ocean. Bulletin of the Tohoku Regional Fisheries Research Laboratory 43: 71-82.

660. Yesaki M (1982) Thailand. Biological and environmental observation. Rome: FAO. 46 p.

661. Yesaki M (1989) Estimates of age and growth of kawakawa (*Euthynnus affinis)*, longtail tuna (*Thunnus tonggol*) and frigate tuna (*Auxis thazard*) from the Gulf of Thailand based on length data. FAO Indo-Pacific Tuna Development and Management Programme IPTP/89/GEN/17. Colombo, Sri Lanka. pp. 94–108.

662. Yesaki M, Carrara G (1994) Age, growth and natural mortality of kawakawa (*Euthynnus affinis*) from the western Indian Ocean. FAO Indo-Pacific Tuna Development and Management Programme Proceeding of the 5th Expert Cunsultation on Indian Ocean Tunas, Mahe, Seychelles, 4-8-October 1993. Colombo, Sri Lanka. pp. 62-66.

663. Yohannan TM (1979) The growth pattern of Indian Mackerel. Indian Journal of Fisheries 25: 207-216.

664. Yohannan TM, Ganga U, Prathibha Rohit, Pillai PP, Radhakrishnan Nair PN, et al. (2002) Stock assessment of mackerel in the Indian seas. In: Pillai NGK, Menon NG, Pillai PP, Ganga U, editors. Management of Scombroid Fisheries: Central Marine Fisheries Research Institute, Kochi. pp. 101-107.

665. Yohannan TM, Jayaprakash AA, Srinath M, Thiagarajan R, Livingston P, et al. (1992) Stock assessment of *Scomberomorus commerson* along the Indian coast. Indian Journal of Fisheries 39: 111 - 118.

666. Yorita T (1981) Maturity of the ovaries of bluefin tuna, *Thunnus thynnus* (Linnaeus) on the Japan Sea off western coast of Hokkaido. Hokkaido Prefecture Fishery Experimental Station Report 38: 211-221.

667. Yoshida HO (1980) Synopsis of biological data on bonitos of the genus Sarda. Rome: FAO. 50 p.

668. Yoshida HO, Nakamura EL (1965) Notes on schooling behavior, spawning, and morphology of Hawaiian frigate mackerels, *Auxis thazard* and *Auxis rochei*. Copeia 1: 111-114.

669. Yuen HSH (1995) Maturity and fecundity of bigeye tuna in the Pacific. Special Scientific Report: Fisheries 150: 1-30.

670. Yukami R, Ohshimo S, Yoda M, Hiyama Y (2009) Estimation of the spawning grounds of chub mackerel *Scomber japonicus* and spotted mackerel *Scomber australasicus* in the East China Sea based on catch statistics and biometric data. Fisheries Science 75: 167-174.

671. Yukinawa M, Yabuta Y (1967) Age and growth of the bluefin tuna, *Thunnus thynnus* (Linnaeus), in the north Pacific Ocean. Report of Nankai Regional Fisheries Research Laboratory 25: 1-18.

672. Zaboukas N, Megalofonou P (2007) Age estimation of the Atlantic bonito in the eastern Mediterranean Sea using dorsal spines and validation of the method. Scientia Marina 71: 691-698.

673. Zafar-Khan M (2004) Age and growth, mortality and stock assessment of *Euthynnus affinis* (Cantor) from Maharashtra waters. Indian Journal of Fisheries 51: 209-213.

674. Zengin M, Karakulak FS, Oray IK (2005) Investigations on bonitos (*Sarda sarda*, Bloch 1793) on the southern Black Sea coast of Turkey. Collective Volume of Scientific Papers, ICCAT 58: 510-516.

675. Zhao CY, Chen LF, Zang ZJ (1982) On the early development and the reproductive behaviour of *Auxis* in Dong Hai, China. Journal of Fisheries of China 6: 243-266.

676. Zhenbin L, Quanshui D, Youming Y, Gangchuan H (1991) Age, growth, and mortality of *Pneumatophorus japonicus* in Minnan-Taiwan Bank fishing ground. 671-678 p.

677. Zhu G, Dai X, Xu L, Zhou Y (2010) Reproductive biology of bigeye tuna, *Thunnus obesus*, (Scombridae) in the eastern and central tropical Pacific Ocean. Environmental Biology of Fishes 88: 253-260.

678. Zhu G, Xu L, Dai X, Liu W (2011) Growth and mortality rates of yellowfin tuna, *Thunnus albacares* (Perciformes: Scombridae), in the eastern and central Pacific Ocean. Zoologia 28: 199–206.

679. Zhu G, Xu L, Zhou Y, Chen X (2009) Growth and mortality rates of bigeye tuna *Thunnus obesus* (Perciformes: Scombridae) in the central Atlantic Ocean. Revista de Biología Tropical 57: 79–88.

680. Zhu G, Xu L, Zhou Y, Song L (2008) Reproductive biology of yellowfin tuna *T. albacares* in the west-central Indian Ocean. Journal of Ocean University of China (Oceanic and Coastal Sea Research) 7: 327-332.

681. Zhu G, Zhou Y, Xu L, Dai X (2009) Growth and mortality of bigeye tuna *Thunnus obesus* (Scombridae) in the eastern and central tropical Pacific Ocean. Environmental Biology of Fishes, 85: 127-137.

682. Zhu GP, Dai XJ, Song LM, Xu LX (2011) Size at sexual maturity of bigeye tuna *Thunnus obesus* (Perciformes: scombridae) in the Tropical waters: a comparative analysis. Turkish Journal of Fisheries and Aquatic Sciences 11: 149–156.

683. Zorica B, Sinovčić G (2008) Biometry , length-length and length-weight relationships of juveniles and adults of Atlantic bonito, *Sarda sarda*, in the eastern middle Adriatic Sea. Acta Adriatica 49: 65 - 72.

684. Zusser SG (1954) Biology and fishery for bonito in the Black Sea. Tr VNIRO 28: 160-174.
