# Supplementary figures and images for: The Conservation and Management of Tunas and Their Relatives: Setting Life History Research Priorities
Source: PLoS One. 2013 Aug 8;8(8):e70405. doi: 10.1371/journal.pone.0070405 (PMC3738557; doi:10.1371/journal.pone.0070405)

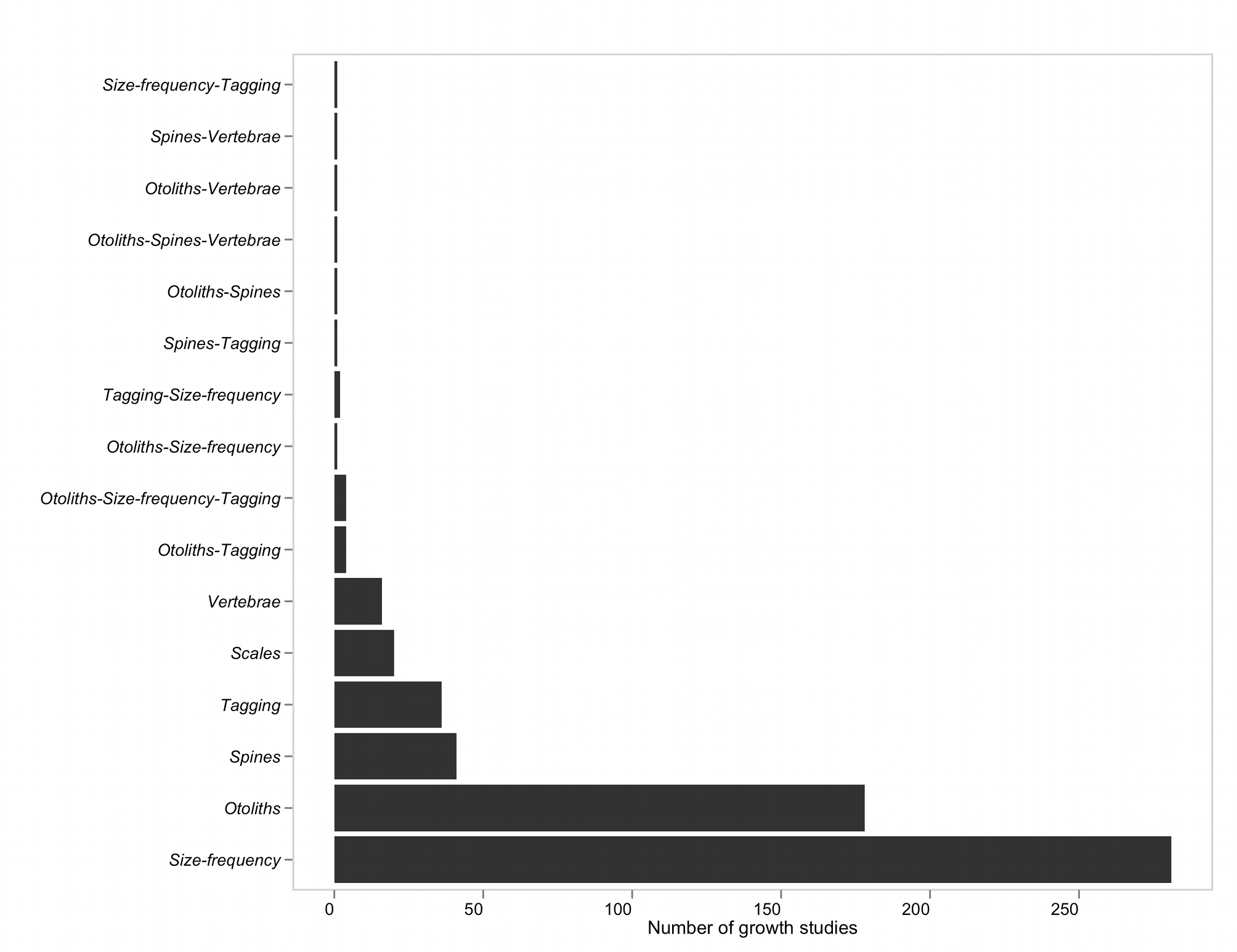

Supplement: Figure S1 — Number of studies to estimate age and growth by method type in scombrid species. Aging methods including direct methods such as calcified structures (vertebrae, spines, scales and otoliths) and indirect methods such as modal analysis of length frequencies and tagging studies, or by various combinations of several of these methods. (TIF) [file pone.0070405.s002.tif]

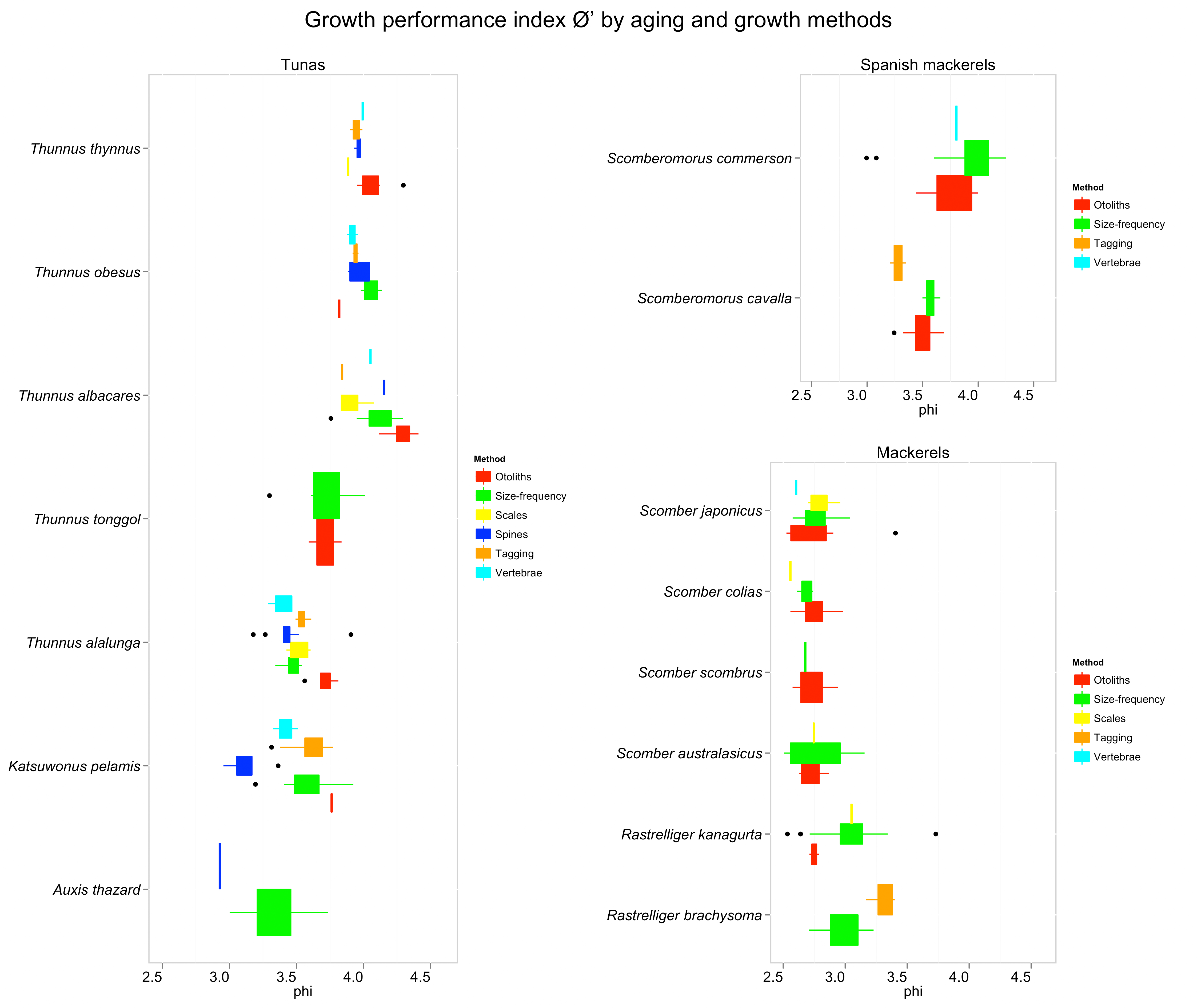

Supplement: Figure S2 — Illustration of the effect of different aging and growth techniques on the estimation of the growth performance index Ø′. Only species for which there are more than 15 von Bertalanffy growth curves are shown. (TIF) [file pone.0070405.s003.tif]

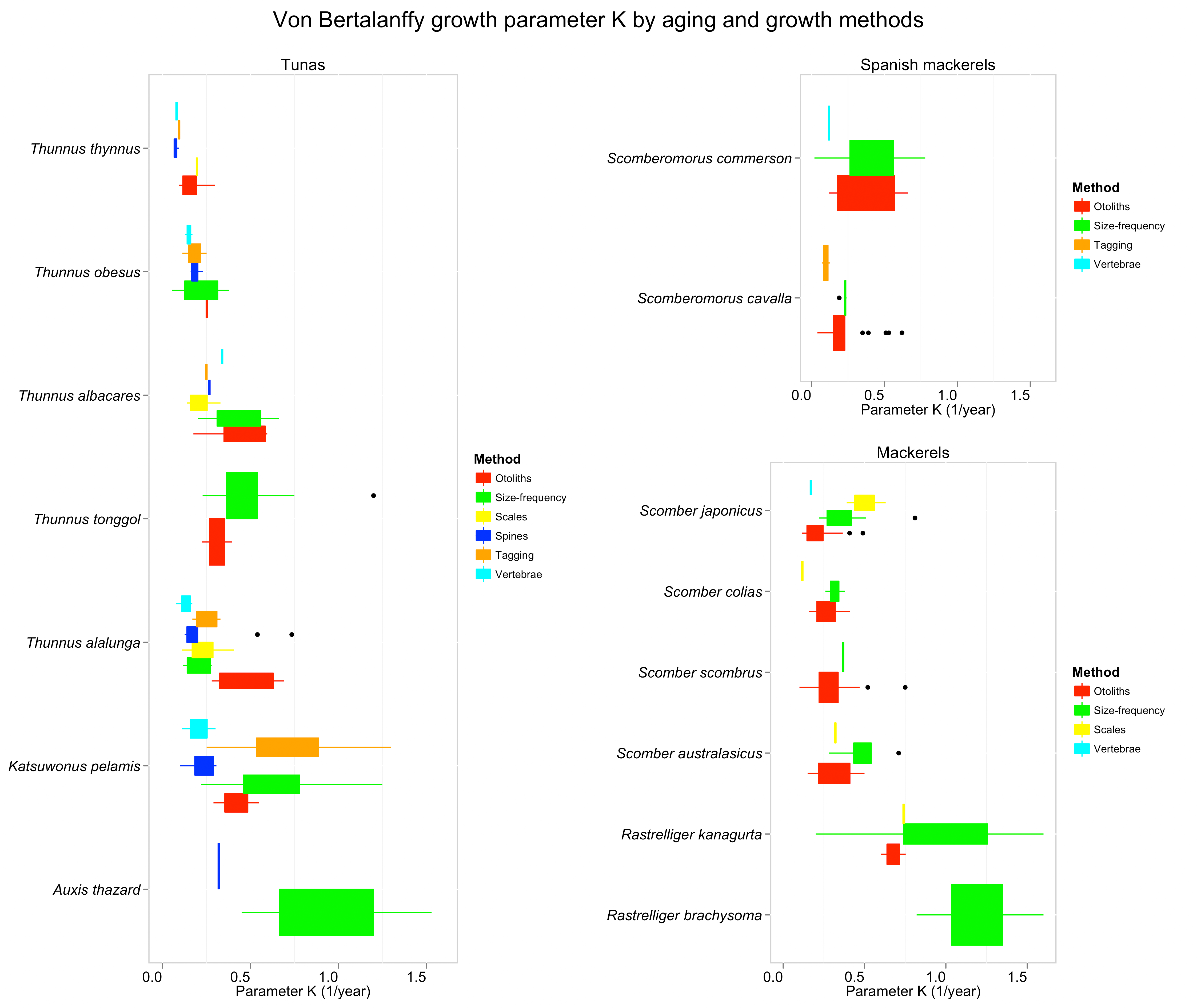

Supplement: Figure S3 — Illustration of the effect of different aging and growth techniques on the estimation of the von Bertalanffy growth parameter k (y−1). Only species for which there are more than 15 von Bertalanffy growth curves are shown. (TIF) [file pone.0070405.s004.tif]

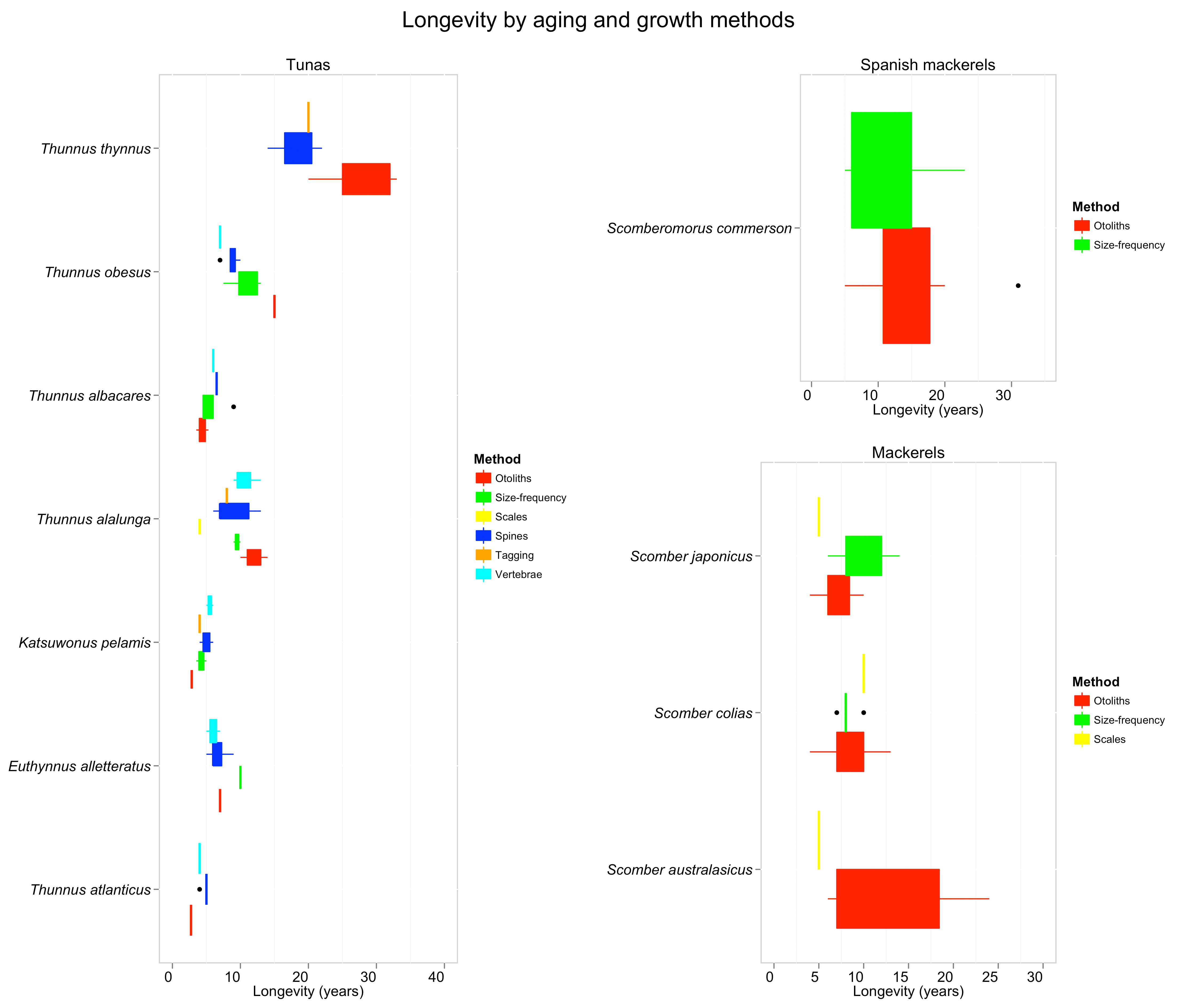

Supplement: Figure S4 — Illustration of the effect of different aging and growth techniques on the estimation of longevity Tmax (y). Only species for which there are more than 15 von Bertalanffy growth curves are shown. (TIF) [file pone.0070405.s005.tif]
