# Supplementary material for: The Conservation and Management of Tunas and Their Relatives: Setting Life History Research Priorities
Source: PLoS One. 2013 Aug 8;8(8):e70405. doi: 10.1371/journal.pone.0070405 (PMC3738557; doi:10.1371/journal.pone.0070405)
Supplement: Table S1 — List of scombrid species with a brief description of their fisheries. (DOC) [file pone.0070405.s006.doc]

**Table S1** List of scombrid species with a brief description of their fisheries.

| **Latin name** | | **Geographical distribution** | **Fisheries** |
| --- | --- | --- | --- |
| **Thunnus alalunga* | | Atlantic, Pacific and Indian oceans, including the Mediterranean Sea | Supports important commercial fisheries globally ranging from large-scale industrial fisheries to small-scale artisanal fisheries throughout their distributions. Traded in the international markets for canning and sashimi. Also caught by recreational fisheries worldwide . |
| **Thunnus albacares* | | Atlantic, Pacific and Indian oceans |  |
| **Thunnus maccoyii* | | Southern waters of the Atlantic, Pacific and Indian oceans |  |
| **Thunnus obesus* | | Atlantic, Pacific and Indian oceans |  |
| **Thunnus thynnus* | | Atlantic Ocean |  |
| **Thunnus orientalis* | | Pacific Ocean |  |
| **Katsuwonus pelamis* | | Atlantic, Pacific and Indian oceans |  |
| *Thunnus atlanticus* | | Western Atlantic Ocean | Caught by commercial fisheries throughout its range, mainly in Venezuela, Martinique, Guadeloupe, Cuba and Dominican Republic . Also important to the local economy in northeastern Brazil . Caught by recreational fisheries in Florida and Bahamas . |
| *Thunnus tonggol* | | Northern Indian Ocean, Indo-Pacific region, Western Pacific Ocean | Caught by small-scale commercial fisheries throughout its range such as in countries bordering the north Arabian Sea and the southeast Asian region . Caught by recreational fisheries in Australia . |
| *Euthynnus affinis* | | Indian Ocean, Indo-Pacific region | Caught by small-scale commercial fisheries throughout its range . The largest catches are reported in the southeast Asian region of Thailand and Malaysia . Also caught by recreational fisheries . |
| *Euthynnus alleteratus* | | Atlantic Ocean, including the Mediterranean and Black seas | Caught by small-scale commercial fisheries throughout its range, in the Mediterranean Sea in countries such as Spain and Turkey and in the Caribbean Sea in countries such as Venezuela . Also caught by recreational fisheries . |
| *Euthynnus lineatus* | | Eastern Pacific Ocean | Caught by small-scale commercial fisheries and as by-catch from other tuna fisheries . Also caught by recreational fisheries . |
| *Auxis rochei* | | Atlantic, Pacific and Indian oceans, including the Mediterranean Sea | Caught by small-scale commercial fisheries throughout its range. Some regions report the catches of *Auxis* sp. mixed, reflecting the confusion surrounding the identification of these species worldwide. It is believed that Atlantic catches are predominantly bullet tuna (*Auxis rochei*) with the highest catches occurring in the Mediterranean Sea and off the northwest African coast where they are commercially exploited by seasonal coastal fisheries . Also caught by recreational fisheries . |
| *Auxis thazard* | | Atlantic, Pacific and Indian oceans | Caught by small-scale commercial fisheries throughout its range. Some regions report the catches of *Auxis* sp. mixed, reflecting the confusion surrounding the identification of these species worldwide. In the Pacific and Indian Ocean the main commercial fisheries exist in Japan, the Philippines, India and the Maldives where most of the catches reported are predominantly frigate tuna (*Auxis thazard*) . Also caught by recreational fisheries . |
| *Allothunnus fallai* | | Southern waters of the Atlantic, Pacific and Indian oceans | There are no developed commercial fisheries for this species. It has no commercial value and it is caught incidentally as by-catch in the longline tuna fisheries in oceanic waters . |
| *Cybiosarda elegans* | | Western Pacific Ocean restricted to the southern coast of Papua Guinea and northern Australia | Caught by commercial fisheries in Australia and used as bait . |
| *Gymnosarda unicolor* | | Disjoint distribution in the Indian Ocean and Indo-Pacific region | Caught by small-scale commercial and recreational fisheries throughout the Indian Ocean and Indo-West Pacific region . Also caught by recreational fisheries . |
| *Orcynopsis unicolor* | | Eastern Atlantic Ocean including Mediterranean Sea | Caught by small-scale commercial fisheries throughout its range in countries such as Tunisia . |
| *Sarda australis* | | Southwest Pacific Ocean in south western Australia and northern New Zealand | Caught by small commercial fisheries and used as bait in Australia . Also caught by recreational fisheries . |
| *Sarda chiliensis* | | Eastern Pacific Ocean | Caught by small-scale commercial fisheries and as by-catch species in tuna purse seine fisheries in the eastern Pacific. The catches reported by IATTC combines the two species of bonito, *S. chiliensis* and *S. orienalis* . Also caught by recreational fisheries . |
| *Sarda orientalis* | | Indian and Pacific Oceans | Caught by small-scale commercial fisheries and as by-catch species in tuna purse seine fisheries in the eastern Pacific. The catches reported by IATTC combines the two species of bonito, *S. chiliensis* and *S. orientalis* . Small commercial fisheries also exist in India and Australia . Also caught by recreational fisheries . |
| *Sarda sarda* | | Atlantic Ocean including Mediterranean Sea | Caught by small-scale commercial fisheries throughout its range and an important commercial species in the Mediterranean and Black Seas . Also caught by recreational fisheries . |
| *Acanthocybium solandri* | | Atlantic, Pacific and Indian oceans, including the Mediterranean Sea | Caught by small-scale commercial fisheries throughout its range. In the Atlantic Ocean the most important known fisheries are found in the east coast of the USA, through the Gulf of Mexico, Caribbean Sea and northern South America . In the Pacific Ocean, the most important fisheries are found in Fiji, Samoa and Cook Islands . In the Indian Ocean, it is caught by commercial fisheries in India together with *S. commerson, S. lineonatus* and *S. guttatus* . Also caught by recreational fisheries . |
| *Scomberomorus brasiliensis* | | Western Atlantic Ocean | Caught by small-scale commercial fisheries in the western central Atlantic waters such as in Trinidad and also off the northwestern coast of Brazil . |
| *Scomberomorus cavalla* | | Western Atlantic Ocean | Sustains one of the most important commercial and recreational fisheries of *Scomberomorus* species in the Atlantic ocean, principally in the southeastern coast of the United States , Gulf of Mexico and Brazil . |
| *Scomberomorus commerson* | | Indian Ocean and Western Pacific Ocean. Recently found in the Mediterranean Sea along the northern African countries. | The most important commercial species of *Scomberomorus* in the Indian Ocean and the Indo-West Pacific region. It is taken throughout its range by small-scale commercial fisheries, sustaining important fisheries in the Arabian Sea , India and Australia . Also caught by recreational fisheries . |
| *Scomberomorus concolor* | | Eastern Central Pacific Ocean. Current distribution is restricted to the northern part of the Gulf of California. | Caught together with *Scomberomorus sierra* by small-scale commerical fisheries in the Gulf of California . |
| *Scomberomorus guttatus* | | Northern Indian Ocean and Indo-Pacific region | Caught by small-scale commercial fisheries throughout its range. Particularly, it sustains an important commercial fishery in India together with *S. commerson, S. lineonatus* and *Acanthocybium solandri* . |
| *Scomberomorus koreanus* | | Northern Indian Ocean and northwestern Pacific Ocean | Caught by small-scale commercial fisheries throughout its range. Particularly, it is caught by small commercial fisheries in India, together with *S. commerson, guttatus* and *lineolatus*, but it forms a negligible portion of the fishery and in China, Korea and Japan together with *S. niphonius* |
| *Scomberomorus lineolatus* | | Northern Indian Ocean and Indo-Pacific region | Caught by small-scale fisheries throughout its range. Particularly, it sustains an important commercial fishery in India together with *S. commerson, S. guttatus* and *Acanthocybium solandri* . |
| *Scomberomorus maculatus* | | Northwestern Atlantic Ocean | Sustains one of the most important commercial and recreational fisheries of *Scomberomorus* species in the Atlantic ocean, principally in the Southeastern Coast of the United States and the Gulf of Mexico . |
| *Scomberomorus multiradiatus* | | Restricted to the Gulf of Papua and Timor Sea in the Indo-Pacific | Caught by small-scale fisheries, particularly as by-catch within its range . |
| *Scomberomorus munroi* | | Indo-Pacific region restricted to northern Australia and Papua New Guinea | Caught by commercial and recreational fisheries in Australia together with *S.commerson, S. queenslandicus* and *S. semifasciatus* . |
| *Scomberomorus niphonius* | | Northwest Pacific Ocean | Caught by commercial fisheries throughout its range, in particular in Japan, South Korea and China . Also caught by recreational fisheries . |
| *Scomberomorus plurilineatus* | | Western Indian Ocean along the Eastern African Coast | Caught by small-scale commercial fisheries throughout its range although the magnitude of the catches is uncertain . It is an important component of the catches by the recreational fisheries in South Africa . |
| *Scomberomorus queenslandicus* | | Indo-Pacific region restricted to northern Australia and Papua New Guinea | Caught by commercial and recreational fisheries in Australia together with *S.commerson, S. munroi* and *S. semifasciatus .* |
| *Scomberomorus regalis* | | Western Atlantic Ocean | Caught by small-scale commercial and recreational fisheries throughout its range in particular in the Caribbean countries such as Jamaica and Puerto Rico . |
| *Scomberomorus semifasciatus* | | Indo-Pacific region restricted to northern Australia and Papua New Guinea | Caught by commercial and recreational fisheries in northern Australia . |
| *Scomberomorus sierra* | | Eastern Pacific Ocean | Caught by small-scale commercial fisheries throughout its range. Particularly it has a well developed commercial fishery in Mexico . Also caught by recreational fisheries . |
| *Scomberomorus sinensis* | | Northwestern Pacific Ocean | Caught by small-scale commercial fisheries throughout its range in particular in Japan, South Korea and China but catch statistics are commonly mixed with other *Scomberomorus* species . Caught by artisanal fleets in the Mekong River of Cambodia . Also caught by recreational fisheries . |
| *Scomberomorus tritor* | | Eastern Atlantic Ocean including the Mediterranean Sea | Caught by small-scale fisheries throughout the eastern Atlantic in particular Senegal and Gambia and caught as bay-catch in the Mediterranean Sea . |
| *Grammatorcynus bicarinatus* | | Southwestern Pacific restricted to the northern coast of Australia | Caught by minor small-scale commercial fisheries but catch statistics data do not exist . Only one known direct fishery for bait exists in Queensland, Australia. Also caught by recreational fisheries . |
| *Grammatorcynus bilineatus* | | Northern Indian Ocean and Indo-Pacific region with a disjoint distribution | Caught by minor small-scale commercial fisheries at least in some parts of its range, for example in Fiji and Andaman Islands but catch statistics data do not exist . Also caught by recreational fisheries . |
| *Rastrelliger brachysoma* | | Indo-Pacific region | Caught by small-scale commercial fisheries throughout its range such as in Malaysia and Philippines . In many parts of its range landings are primarily reported in combination with mixed *Rastrelliger* spp. |
| *Rastrelliger faughni* | | Indo-Pacific region | Caught by minor small-scale commercial fisheries throughout its range and it is the least abundant among the *Rastrelliger* sp. In many parts of its range landings are primarily reported in combination with mixed *Rastrelliger* spp . |
| *Rastrelliger kanagurta* | | Indian Ocean, Indo-Pacific region and Western Pacific Ocean | Caught by small-scale commercial fisheries throughout its range such as in India , Philippines and Egypt . In many parts of its range landings are primarily reported in combination with mixed *Rastrelliger* spp. |
| *Scomber australasicus* | | Western Pacific Ocean and northwestern Indian Ocean | Caught by commercial fisheries throughout its range such as in New Zealand , Australia , Taiwan , Japan and Gulf of Suez . |
| *Scomber japonicus* | | Northwest Pacific Ocean and Eastern Pacific Ocean | Caught by commercial fisheries throughout its range in particular in China , Japan , Chile and California . |
| *Scomber scombrus* | | Northwest Atlantic Ocean and Northeast Atlantic Ocean including the Mediterranean Sea | Caught by commercial fisheries throughout its range, particularly in northwestern Europe , Canada and USA . |
| *Scomber colias* | | Eastern Atlantic Ocean including Mediterranean Sea | Caught by commercial fisheries throughout its range such as in Spain and the Hellenic Seas . |
| *Gasterochisma melampus* | Southern waters of the Atlantic, Pacific and Indian oceans | | There are no developed commercial fisheries for this species. It has no commercial value and it is caught incidentally as by-catch in the longline tuna fisheries in oceanic waters . |

* Commonly known as principal market tuna species.

**References (Table S1)**

1. Majkowski J (2007) Global fishery resources of tuna and tuna-like species. FAO Fisheries Technical Paper 483: 1-54.

2. Miyake MP, Guillotreau P, Sun C, Ishimura G (2010) Recent developments in the tuna industry. Stocks, fisheries, management, processing, trade and markets. FAO Fisheries and Aquaculture Technical Paper 536: 1-125.

3. IGFA (2010) Database of International Game Fish Association angling records until 2010. Fort Lauderdale, United States: IGFA.

4. Báez-Hidalgo M, Bécquer U (1994) Fecundidad del bonito Katsowonus pelamis (Linnaeus) y albacora Thunnus atlanticus (Lesson) en Cuba. Revista de Investigaciones Marinas 15: 218-222.

5. Doray M, Reynal L, Carpentier A (2002) Les pêcheries de poissons pélagiques hauturiers aux Petites Antilles en 2001. Supplement to the Report of the First meeting of the WECAFC Ad-hoc Working Group on the Development of Sustainable Moored Fish Aggregating Device Fishing in the Lesser Antilles. FAO Fisheries Report 683: 55-68.

6. Freire KM, Lessa F, R, Lins-Oliveira JE (2005) Fishery and biology of Blackfin tuna (Thunnus atlanticus) off Northeastern Brazil. Gulf and Caribbean Research 17: 15-24.

7. Collette BB (2002) Scombridae. In: Carpenter k, editor. The living marine resources of the western central Atlantic Volume 3: Bony fishes part 2 (Opistognathidae to Molidae), sea turtles and marine mammals FAO Species Identification Guide for Fishery Purposes and American Society of Ichthyologists and Herpetologists Special Publication No 5. Rome, Italy: Food and Agriculture organization of the United Nations (FAO). pp. 1375-2127.

8. Yesaki M (1994) A review of the biology and fisheries of the longtail tuna (*Thunnus tonggol*) in the Indo-Pacific region. FAO Fisheries Technical Paper 336: 370-387.

9. IOTC (2006) Executive summary of the status of fisheries resources. Victoria, Seychelles.

10. Abdussamad EM, Koya KPS, Ghosh S, Joshi KK, Manojkumar B, et al. (2012) Fishery, biology and population characteristics of longtail tuna, Thunnus tonggol (Bleeker, 1851) caught along the Indian coast. Indian Journal of Fisheries 59: 7-16.

11. Peters-Mason A, Roberts S (2009) Tongol tuna Thunnus tonggol,Thailand, Indonesia, Malaysia and Iran. Monterey Bay Aquarium: Seafood Watch Report. 1-64 p.

12. Griffiths S, Pepperell J, Tonks M, Sawynok W, Olyott L, et al. (2010) Biology, Fisheries and Status of Longtail Tuna (Thunnus Tonggol), with Special Reference to Recreational Fisheries in Australian Waters. Final Report FRDC Project 2008/058.

13. Chiou WD, Lee LK (2004) Migration of kawakawa Euthynnus affinis in the waters near Taiwan. Fisheries Science 70: 746-757.

14. Zafar-Khan M (2004) Age and growth, mortality and stock assessment of *Euthynnus affinis* (Cantor) from Maharashtra waters. Indian Journal of Fisheries 51: 209-213.

15. Al-zibdah M, Odat N (2007) Fishery status, growth, reproduction, biology and feeding habit of two scombrid fish from the Gulf of Aqaba, Red Sea. Lebanese Science Journal 8: 3-20.

16. Taghavi Motlagh SA, Hashemi SA, Kochanian P (2010) Population biology and assessment of kawakawa (Euthynnus affinis) in coastal waters of the Persian Gulf and Sea of Oman (Hormozgan Province). Iranian Journal of Fisheries Sciences 9: 315–326.

17. Kahraman AE, Oray IK (2001) The determination of age and growth parameters of atlantic little Tunny Euthynnus alleteratus (Rafinesque, 1810) in turkish waters. Collective Volume of Scientific Papers, ICCAT 52: 719-732.

18. Ramírez-Arredondo I (1993) Aspectos reproductivos de la carachana pintada, Euthynnus alletteratus (Pisces:Scombridae) de los alrededores de la Isla de Picua, Estado Sucre, Venezuela. Boletín del Instituto Oceanográfico de Venezuela 32: 69-78.

19. IATTC (2008) The fishery for tunas and billfishes in the eastern Pacific Ocean in 2007. La Jolla, California, USA.

20. Di Natale A, Srour A, Hattour A, Keskin C, Idrissi MH, et al. (2009) Regional study on small tunas in the Mediterranean including the Black Sea. Studies and Reviews. General Fisheries Commission for the Mediterranean. No 35. Rome: Food and Agricultural Organization (FAO). 132 p.

21. Yesaki M, Arce F (1993) A review of the Auxis fisheries of the Philippines and some aspects of the biology of frigate (A. thazard) and bullet (A. rochei) tunas in the Indo-Pacific region. In: Shomura RS, Majkowski J, Langi S, editors. Interactions of Pacific tuna fisheries Proceedings of the first FAO Expert Consultation on Interactions of Pacific Tuna Fisheries 3–11 December 1991 Noumea, New Caledonia Volume 2: papers on biology and fisheries FAO Fisheries Technical Paper No 336, Vol2. Rome: FAO. pp. 409-439.

22. FAO (2008) FISHSTAT Plus- Universal software for fishery statistical time series. Food and Agriculture Organization of the United Nations, Rome.

23. Ghosh S, Sivadas M, Abdussamad EM, Rohit P, Koya KPS, et al. (2012) Fishery, population dynamics and stock structure of frigate tuna Auxis thazard (Lacepede , 1800) exploited from Indian waters. Indian Journal of Fisheries 59: 95-100.

24. Warashina I, Hisada K (1972) Geographical distribution and body length composition of two tuna-like fishes, Gasterochisma melampus Richardson and Allothunnus fallai Serventy, taken by Japanese tuna longline fishery. Bulletin of Far Sea Fisheries Research Laboratory 6: 51-75.

25. Scandol J, Rowling K, Graham K (2008) Status of Fisheries Resources in NSW 2006/07. Cronulla: NSW Department of Primary Industries. 334 p.

26. Collette B, Carpenter KE, Nelson R, Fox W (2011) Cybiosarda elegans. IUCN 2012 IUCN Red List of Threatened Species Version 20122 <wwwiucnredlistorg> Downloaded on 07 January 2013.

27. Collette BB (1998) Scombridae. In: Carpenter KE, Niem VH, editors. The living marine resources of the western central Pacific Volume 6 Bony fishes part 4 (Labridae to Latimeriidae), estuarine crocodiles, sea turtles, sea snakes and marine mammals. Rome, Italy: Food and Agriculture organization of the United Nations (FAO). pp. 3381-4218.

28. Joshi KK, Abdussamad EM, Koya KPS, Sivadas M, Kuriakose S, et al. (2012) Fishery, biology and dynamics of dogtooth tuna, Gymnosarda unicolor (Rüppell , 1838) exploited from Indian seas. Indian Journal of Fisheries 59: 75–79.

29. Hattour A (2000) Contribution à l’étude des poissons pélagiques des eaux tunisiennes [Thèse de Doctorat]. Tunis: Université d’El Manar II. 327 p.

30. Sivadas M, Abdussamad EM, Jasmine S, Rohit P, Koya KPS, et al. (2012) Assessment of the fishery and stock of striped bonito, Sarda orientalis (Temminck and Schlegel, 1844) along Kerala coast with a general description of its fishery from Indian coast. Indian Journal of Fisheries 59: 57–61.

31. Oxenford HA, Murray PA, Luckhust BE (2003) The biology of wahoo (Acanthocybium solandri) in the western central Atlantic. Gulf and Caribbean Research 15: 33-39.

32. Zischke MT (2012) A review of the biology, stock structure, fisheries and status of wahoo (*Acanthocybium solandri*), with reference to the Pacific Ocean. Fisheries Research 119: 13-22.

33. Ghosh S, Pillai NGK, Dhokia HK (2009) Fishery, population dynamics and stock assessment of the spotted seer in gill net fishery at Verabal. Indian Journal of Fisheries 56: 157-161.

34. Siddeek MSM (1995) Review of fisheries biology of Scomberomorus and Acanthocybium species in the Western Indian Ocean (FAO Area 51). Working Group on Pelagics, Gulfs Committee for Fisheries Management and Development Working Paper WGP 95/2. pp. 32.

35. Gold JR, Jobity AMC, Saillant E, Renshaw MA (2010) Population structure of carite (*Scomberomorus brasiliensis*) in waters offshore of Trinidad and northern Venezuela. Fisheries Research 103: 30-39.

36. Batista VS, Fabré NN (2001) Temporal and spatial patterns on Serra, Scomberomorus Brasiliensis (Teleostei scombridae), catches from the fisheries on the Maranhào Coast, Brazil. Brazilian Journal of Biology 61: 541-546.

37. Sutter IFC, Williams RO, Godcharles MF (1991) Growth and mortality of king mackerel Scomberomorus cavalla tagged in the Southeastern United States. Fishery Bulletin 89: 733-737.

38. Arreguín-Sánchez F, Cabrera MA, Aguilar FA (1995) Population dynamics of the king mackerel (*Scomberomorus cavalla*) of the Campeche Bank, Mexico. Scientia Marina 59: 637-645.

39. de Nóbrega MF, Lessa RP (2009) Age and growth of the king mackerel (*Scomberomorus Cavalla*) off the northeastern coast of Brazil. Brazilian Journal of Oceanography 57: 273-285.

40. Ben-Meriem S, Al-Marzouqi A, Al-Mamry J (2006) Fisheries exploitation pattern of narrow-barred Spanish mackerel, *Scomberomorus commerson*, in Oman and potential management options. Journal of Applied Ichthyology 22: 218-224.

41. Pillai NGK, Pillai PP, Said-Koya KP, Sathianandan TV (1996) Assessment of the stock of kingseer, *Scomberomorus commerson* (Lacepede), along the west coast of India. In: Anganuzzi AA, Stobberup KA, Webb NJ, editors. FAO Indo-Pacific Tuna Development and Management Programme Collective volume of working documents presented at the Expert Consultation on Indian Ocean Tunas, 25-29 September 1995. Colombo, Sri Lanka. pp. 299-311.

42. Tobin AJ, Mapleston A (2004) Exploitation dynamics and biological characteristics of the Queensland east coast Spanish mackerel fishery. Fisheries Research and Development Corporation, Final Report 2001/019.

43. Montemayor-López G, Cisneros-Mata MA (2000) La sierra del Golfo de California. México: Secretaría de Medio Ambiente, Recursos Naturales y Pesca - Instituto Nacional de la Pesca.

44. Kasim HC, Muthiah NG, Pillai K, Yohannan TM, Manojkumar B, et al. (2002) Stock assessment of seerfishes in the Indian Seas. In: Pillai NGK, Menon NG, Pillai PP, Ganga U, editors. Management of scombroid fisheries. Kochi, India: Central Marine Fisheries Research Institute. pp. 108-124.

45. Seikai National Fisheries Research Institute (2001) Biological and ecological characteristics of valuable fisheries resources from the East China Sea and the Yellow sea –comparison between the Chinese and Japanese knowledge (in Japanese). Seikai National Fisheries Research Institute, Nagasaki.

46. Ansley H, Gregory R, Meserve N, Waugh G (2006) Review of the fishery management plan for Spanish Mackerel (*Scomberomorus maculatus*). Prepared by Spanish Mackerel Plan Review Team. 1-13 p.

47. Medina-Quej A, Domínguez-Viveros M (1997) Edad y crecimiento del Scomberomorus maculatus (Scombriformes: Scombridae) en Quintana Roo, México. Revista de Biología Tropical 45: 1155-1161.

48. Collette BB (2001) Family Scombridae. In: Carpenter KE, Niem VH, editors. FAO species identification guide for fishery purposes The living marine resources of the Western Central Pacific Volume 6 Bony fishes part 4 (Labridae to Latimeriidae), estuarine crocodiles, sea turtles, sea snakes and marine mammals. Rome, Italy: Food and Agricultural Organization (FAO). pp. 3381-4218.

49. Begg GA, Cameron DS, Sawynok W (1997) Movements and stock structure of school mackerel (*Scomberomorus queenslandicus*) and spotted mackerel (S. munroi) in Australian east-coast waters. Marine Freshwater Research 48: 295-301.

50. Obata Y, Yamakazi H, Iwamoto A, Hamasaki K, Kitada S (2008) Evaluation of Stocking Effectiveness of the Japanese Spanish Mackerel in the Eastern Seto Inland Sea, Japan. Reviews in Fisheries Science 16: 235-242.

51. Xianshi J (2008) Biological Assessment of Ecologically Important Areas for Fish and Invertebrate Taxonomic Groups of the Yellow Sea Ecoregion. Biological Assessment Report of the Yellow Sea Ecoregion

52. Collette BB, Nauen CE (1983) FAO Species Catalogue. Vol. 2. Scombrids of the world: an annotated and illustrated catalogue of tunas, mackerels, bonitos and related species known to date. FAO Fisheries Synopsis 125: 137.

53. Chale-Matsau JR, Govender A, Beckley LE (1999) Age and growth of the queen mackerel *Scomberomorus plurilineatus* from KwaZulu-Natal, South Africa. Fisheries Research 44: 121-127.

54. Cooper A (2003) A preliminary study of the fishery for cero Mackerel (Scomberomorus regalis, Bloch) in Jamaican Waters. Proceedings Gulf and Caribbean Fisheries Institute. Mona, Jamaica. pp. 149-155.

55. Figuerola-Fernández M, Torres-Ruiz W. Madurez sexual y estacionalidad reproductiva del carite (Scomberomorus cavalla) y la sierra (S. regalis) en Puerto Rico; 2003; San Andrés, Colombia. pp. 250-261.

56. Welch DJ, Buckworth RC, Ovenden JR, Newman SJ, Broderick D, et al. (2009) Determination of management units for grey mackerel fisheries in northern Australia. Townsville, Queensland, Australia: Report. James Cook University.

57. Aguirre-Villaseñor H, Morales-Bojórquez E, Morán-Angulo RE, Madrid-Vera J, Valdez-pineda MC (2006) Indicadores biolóogicos de la pesquería de sierra (Scomberomorus sierra) al sur del Golfo de California, México. Ciencias Marinas 32: 471-484.

58. Ni I-H, Kwok K-Y (1999) Marine fish fauna in Hong Kong waters. Zoological Studies 38: 130–152.

59. Samb B, Mendy AN (2004) Dynamique du réseau trophique de l’écosystème sénégambien en 1990. In: Palomares MLD, Pauly D, editors. West African marine ecosystems: models and fisheries impactsFisheries Centre Research Reports 12(7). Vancouver: Fisheries Centre, UBC. pp. 57-70.

60. Collette B, Fox W, Nelson R (2011) Grammatorcynus bicarinatus. IUCN 2012 IUCN Red List of Threatened Species Version 20122 <wwwiucnredlistorg> Downloaded on 07 January 2013.

61. Phaik-Ean C (2000) Status of the Kembong Fishery on the West Coast of Peninsular Malaysia Fishcode management, supplement to the report of a workshop on the fishery and management of a short mackerel (Rastrelliger spp) on the West Coast of Peninsular Malaysia GCP/INT/648/NOR. Rome: FAO. pp. 1-20.

62. Rohit P, Gupta AC (2004) Fishery, biology and stock of the Indian mackerel Rastrelliger kanagurta off Mangalore-Malpe in Karnataka, India. Journal of the Marine Biological Association of India 46: 185-191.

63. Trinidad AC, Pomeroy RS, Corpuz PV, Aguero M (1993) Bioeconomics of the Philippine small pelagic fishery. ICLARM Technical Report 38: 74.

64. BOBP (1987) Investigations on the mackerel and scad resources of the Malacca Straits. Colombo, Sri Lanka. 1-155 p.

65. Abdussamad EM, Pillai NGK, Kasim HM, Mohamed OMMJH, Jeyabalan K (2010) Fishery, biology and population characteristics of the Indian mackerel, Rastrelliger kanagurta (Cuvier) exploited along the Tuticorin coast. Indian Journal of Fisheries 57: 17–21.

66. Mehanna SF (2001) Population dynamics and fisheries management of Indian mackerel *Rastrelliger kanagurta* in the Gulf of Suez, Egypt. Journal of King Abdulaziz University - Marine Sciences 12: 217-229.

67. Morrison M, Taylor P, Marriott P, Sutton C (2001) An assessment of information on blue mackerel (*Scomber australasicus*) stocks. New Zealand Fisheries Assessment Report 2001/44. 26 p.

68. Ward PJ, Timmiss T, Wise B (2001) A review of biology and fisheries for mackerel. Canberra: Bureau of Rural Sciences. 120 p.

69. Bolaños MA, Tzeng WN (1994) Estimation of growth parameters of two species of Mackerel, Scomber japonicus and S. australasicus, in the Coastal Waters of Taiwan. Journal of The Fisheries Society of Taiwan 21: 313-321.

70. Yukami R, Asano K, Yoda M, Ooshimo S, Tanaka H (2009) Stock assessment and evaluation for East China Sea stock of spotted mackerel (fiscal year 2008). Marine fisheries stock assessment and evaluation for Japanese waters (fiscal year 2008/2009): Fisheries Agency and Fisheries Research Agency of Japan. pp. 219-242.

71. Mehanna SF (2004) Maximum sustainable yield of the round herring, Etrumeusteres and slimy mackerel, Scomber japonicus in the Gulf of Suez. Egyptian Journal of Aquatic Research 30: 322-325.

72. Hiyama Y, Yoda M, Ohshimo S (2002) Stock size fluctuations in chub mackerel ( Scomber japonicus ) in the East China Sea and the Japan/East Sea. Fisheries Oceanography 11: 347-353.

73. Watanabe C, Yatsu A (2006) Long-term changes in maturity at age of chub mackerel (*Scomber japonicus*) in relation to population declines in the waters off northeastern Japan. Fisheries Research 78: 323-332.

74. Canales CR (2006) Investigación, evaluación de stock y CTP Caballa 2005-Informe Final. Valparaiso, Chile: Instituto de Fomento Pesquero. 40 p.

75. Dorval E, Hill KT, Lo NCH, McDaniel JD (2007) Pacific Mackerel (Scomber japonicus) Assessment for U.S. management in the 2007-2008 fishing season. Pacific Fishery Management Council, June 2007 Briefing Book, Agenda Item G1b, Attachement 1. pp. 78.

76. ICES (2009) Report of the working group on the assessment of mackerel, horse mackerel, sardine and anchovy (WGMHMSA).

77. DFO (2008) Assessment of the Atlantic mackerel stock for the Northwest Atlantic (subareas 3 and 4) in 2007 Quebec, Canada: DFO Canadian Science Advisory Secretariat Science Advisory Report 2008/041. 15 p.

78. Velasco EM, Del Arbol J, Baro J, Sobrino I (2011) Age and growth of the Spanish chub mackerel *Scomber colias* off southern Spain: a comparison between samples from the NE Atlantic and the SW Mediterranean. Revista de Biologia Marina y Oceanografia 46: 27–34.

79. Kiparissis S, Tserpes G, Tsimenidis N (2000) Aspects on the demography of Chub Mackerel (Scomber japonicus Houttuyn, 1782) in the Hellenic Seas. Belgian Journal of Zoology 130: 3-7.
