# Supplementary material for: The Conservation and Management of Tunas and Their Relatives: Setting Life History Research Priorities
Source: PLoS One. 2013 Aug 8;8(8):e70405. doi: 10.1371/journal.pone.0070405 (PMC3738557; doi:10.1371/journal.pone.0070405)
Supplement: Table S2 — Criteria to construct the Venn Diagram of life history research priorities in scombrid species. We differentiated between life history data-poor and data-rich species (see definition in main text), between species targeted and not-targeted by commercial fisheries (see Table S1), and between species listed as Threatened, Near Threatened and Data Deficient from those listed as Least Concern in the IUCN Red List [10]. IUCN Red List categories: CR - Critically Endangered, EN - Endangered, VU - Vulnerable, NT - Near Threatened, LC - Least Concern and DD - Data Deficient. (DOC) [file pone.0070405.s007.doc]

**Table S2** Criteria to construct the Venn Diagram of life history research priorities in scombrid species. We differentiated between life history data-poor and data-rich species (see definition in main text), between species targeted and not-targeted by commercial fisheries (see Table S1), and between species listed as Threatened, Near Threatened and Data Deficient from those listed as Least Concern in the IUCN Red List [10]. IUCN Red List categories: CR - Critically Endangered, EN - Endangered, VU - Vulnerable, NT - Near Threatened, LC - Least Concern and DD - Data Deficient.

| **Taxonomic**  **group** | **Latin name** | **Life history research** | **Fisheries** | **IUCN**  **Status** |
| --- | --- | --- | --- | --- |
| Tunas | *Allothunnus fallai* | Data-poor | Not-targeted | LC |
|  | *Auxis rochei* | Data-rich | Targeted | LC |
|  | *Auxis thazard* | Data-poor | Targeted | LC |
|  | *Euthynnus affinis* | Data-rich | Targeted | LC |
|  | *Euthynnus alletteratus* | Data-poor | Targeted | LC |
|  | *Euthynnus lineatus* | Data-rich | Targeted | LC |
|  | *Katsuwonus pelamis* | Data-rich | Targeted | LC |
|  | *Thunnus alalunga* | Data-rich | Targeted | NT |
|  | *Thunnus albacares* | Data-rich | Targeted | NT |
|  | *Thunnus atlanticus* | Data-poor | Targeted | LC |
|  | *Thunnus maccoyii* | Data-rich | Targeted | CR |
|  | *Thunnus obesus* | Data-rich | Targeted | VU |
|  | *Thunnus orientalis* | Data-poor | Targeted | LC |
|  | *Thunnus thynnus* | Data-rich | Targeted | EN |
|  | *Thunnus tonggol* | Data-poor | Targeted | DD |
| Bonitos | *Cybiosarda elegans* | Data-poor | Targeted | LC |
|  | *Gymnosarda unicolor* | Data-poor | Targeted | LC |
|  | *Orcynopsis unicolor* | Data-poor | Targeted | LC |
|  | *Sarda australis* | Data-poor | Targeted | LC |
|  | *Sarda chiliensis* | Data-poor | Targeted | LC |
|  | *Sarda orientalis* | Data-poor | Targeted | LC |
|  | *Sarda sarda* | Data-rich | Targeted | LC |
| Spanish mackerels | *Acanthocybium solandri* | Data-rich | Targeted | LC |
|  | *Grammatorcynus bicarinatus* | Data-poor | Targeted | LC |
|  | *Grammatorcynus bilineatus* | Data-poor | Targeted | LC |
|  | *Scomberomorus brasiliensis* | Data-poor | Targeted | LC |
|  | *Scomberomorus cavalla* | Data-rich | Targeted | LC |
|  | *Scomberomorus commerson* | Data-rich | Targeted | NT |
|  | *Scomberomorus concolor* | Data-poor | Targeted | VU |
|  | *Scomberomorus guttatus* | Data-poor | Targeted | DD |
|  | *Scomberomorus koreanus* | Data-poor | Targeted | LC |
|  | *Scomberomorus lineolatus* | Data-poor | Targeted | LC |
|  | *Scomberomorus maculatus* | Data-rich | Targeted | LC |
|  | *Scomberomorus multiradiatus* | Data-poor | Targeted | LC |
|  | *Scomberomorus munroi* | Data-poor | Targeted | VU |
|  | *Scomberomorus niphonius* | Data-poor | Targeted | DD |
|  | *Scomberomorus plurilineatus* | Data-poor | Targeted | DD |
|  | *Scomberomorus queenslandicus* | Data-poor | Targeted | LC |
|  | *Scomberomorus regalis* | Data-poor | Targeted | LC |
|  | *Scomberomorus semifasciatus* | Data-poor | Targeted | LC |
|  | *Scomberomorus sierra* | Data-poor | Targeted | LC |
|  | *Scomberomorus sinensis* | Data-poor | Targeted | DD |
|  | *Scomberomorus tritor* | Data-poor | Targeted | LC |
| Mackerels | *Rastrelliger brachysoma* | Data-poor | Targeted | DD |
|  | *Rastrelliger faughni* | Data-poor | Targeted | DD |
|  | *Rastrelliger kanagurta* | Data-poor | Targeted | DD |
|  | *Scomber australasicus* | Data-rich | Targeted | LC |
|  | *Scomber colias* | Data-poor | Targeted | LC |
|  | *Scomber japonicus* | Data-rich | Targeted | LC |
|  | *Scomber scombrus* | Data-rich | Targeted | LC |
| Gasterochisma | *Gasterochisma melampus* | Data-poor | Not-targeted | LC |
